# The two-component system TtrRS boosts *Vibrio parahaemolyticus* colonization by exploiting sulfur compounds in host gut

**DOI:** 10.1371/journal.ppat.1012410

**Published:** 2024-07-22

**Authors:** Xiaojun Zhong, Fuwen Liu, Tianqi Liang, Ranran Lu, Mengting Shi, Xiujuan Zhou, Menghua Yang

**Affiliations:** College of Animal Science and Technology, College of Veterinary Medicine, Zhejiang A & F University, Key Laboratory of Applied Technology on Green-Eco-Healthy Animal Husbandry of Zhejiang Province, Zhejiang Provincial Engineering Laboratory for Animal Health Inspection & Internet Technology, Hangzhou, China; University of Rochester Medical Center, UNITED STATES OF AMERICA

## Abstract

One of the greatest challenges encountered by enteric pathogens is responding to rapid changes of nutrient availability in host. However, the mechanisms by which pathogens sense gastrointestinal signals and exploit available host nutrients for proliferation remain largely unknown. Here, we identified a two-component system in *Vibrio parahaemolyticus*, TtrRS, which senses environmental tetrathionate and subsequently activates the transcription of the *ttrRS*-*ttrBCA-tsdBA* gene cluster to promote *V. parahaemolyticus* colonization of adult mice. We demonstrated that TsdBA confers the ability of thiosulfate oxidation to produce tetrathionate which is sensed by TtrRS. TtrRS autoregulates and directly activates the transcription of the *ttrBCA* and *tsdBA* gene clusters. Activated TtrBCA promotes bacterial growth under micro-aerobic conditions by inducing the reduction of both tetrathionate and thiosulfate. TtrBCA and TsdBA activation by TtrRS is important for *V. parahaemolyticus* to colonize adult mice. Therefore, TtrRS and their target genes constitute a tetrathionate-responsive genetic circuit to exploit the host available sulfur compounds, which further contributes to the intestinal colonization of *V. parahaemolyticus*.

## Author summary

Access to host nutrients is regarded as a critical step for the pathogen infection. However, the human gastrointestinal tract is colonized by an enormous number of microbes, which limits pathogens’ access to nutrient supplies. Sulfur is one of the most abundant elements on Earth and is also essential to all living organisms. The mechanisms by which bacteria, especially intestinal pathogens, regulate the bioavailability of utilizable sulfur in the host are poorly understood. Here, we characterized a tetrathionate-responsive genetic circuit, which was involved in the utilization of sulfur compounds including tetrathionate and thiosulfate in *V. parahaemolyticus*, and thus contributed to the bacterial intestinal colonization. Our study provides new insight into the ability of bacterial pathogens to sense gastrointestinal signals and exploit host nutrients for their colonization, which contributes to a better understanding of the pathogenesis of enteric pathogens.

## Introduction

*Vibrio parahaemolyticus*, the microorganism responsible for seafood-derived food poisoning, is a significant cause of acute gastroenteritis worldwide [1]. It commonly thrives in warm climates within marine or estuarine environments and causes disease through the consumption of raw or undercooked contaminated seafood [1,2]. From persisting in aquatic environments to active colonization of the human gastrointestinal tract, *V. parahaemolyticus* cells are exposed to a variety of environmental changes, such as pH, osmolarity, oxygen, and nutrient sources. After colonized in host gut, *V. parahaemolyticus* further invades the cecal mucosa and causes severe inflammation accompanied by dramatic mucosal damage [3]. Pathogenic bacteria must effectively sense external signals and regulate intracellular pathways to acclimatize to an appropriate niche during the infection [4,5]. Although the regulatory mechanism of virulence gene expression in *V. parahaemolyticus* has been intensely investigated [6–10], the mechanism that facilitates *V. parahaemolyticus* colonization in the host gut remains incompletely understood.

To survive and thrive in host niches, pathogenic bacteria have evolved diverse regulators including two-component systems (TCSs), to swiftly respond to various stress conditions [11,12]. TCSs, typically comprised of a membrane-bound sensor histidine kinase (HK) and a cognate cytoplasmic response regulator (RR), are the most abundant multistep signaling pathways in prokaryotes [12]. The genome of *V. parahaemolyticus* encodes at least 50 TCSs, and certain TCSs have been shown to respond to external signals or cues. For example, VbrK, the HK of VbrK/VbrR TCS, directly binds to β-lactam antibiotics, leading to the expression of a β-lactamase and resistance to β-lactam antibiotics [13]. Under iron-limiting conditions, PeuRS functions in concert with extracellular alkaline pH and enterobactin for the induction of PeuA, which is responsible for enterobactin utilization [14]. Recently, several TCSs have been identified to assist *V. parahaemolyticus* in innate immune regulation at the early stage of infection in THP-1 cell-derived macrophages [15]. So far, the mechanisms by which *V. parahaemolyticus* senses and responds to the gastrointestinal signals by TCSs for their proliferation remain unclear.

In this study, a TCS, homology to TtrRS of *Salmonella typhimurium*, was found to contribute to *V. parahaemolyticus* colonization in host gut. Previous study indicated that TtrRS is required for the transcription of tetrathionate reductase TtrBCA in *S*. *typhimurium* [16]. In the intestinal lumen, gut microbiota produces large quantities of hydrogen sulfide (H_2_S) which could be converted to thiosulfate (S_2_O_3_^2-^) by the cecal mucosa [17,18]. *S*. *typhimurium* could use type III secretion systems (T3SSs) to trigger acute intestinal inflammation, which oxidizes endogenous thiosulfate to generate tetrathionate (S_4_O_6_^2-^) [19]. *S*. *typhimurium* has the ability to respire with tetrathionate as an electron acceptor by TtrBCA, and thus outgrow the intestinal microbiota lacking this capacity [19]. We found that *S*. *typhimurium* hosts thiosulfate reductase but lacks a thiosulfate oxidation system, whereas *V. parahaemolyticus* genome encodes both the thiosulfate reductase and oxidase. Here, we demonstrated that the TtrRS target genes *tsdBA* confers the ability to oxidize thiosulfate to *V. parahaemolyticus*, which produces tetrathionate, a gastrointestinal signal for TtrRS, and further activates the TCS. TtrRS subsequently directly regulates the transcription of the *ttrRS*-*ttrBCA*-*tsdBA* gene cluster and thus forms a tetrathionate-responsive genetic circuit. We further found that TtrRS mediated genetic circuit contributes to the sulfur utilization and intestinal colonization of *V. parahaemolyticus*. Together, our study revealed a biochemical pathway by which bacteria detect environmental sulfur and regulate their colonization accordingly.

## Results

### 1 TtrRS is important for *V. parahaemolyticus* to colonize in the intestine of adult mice

**Fig 1:**
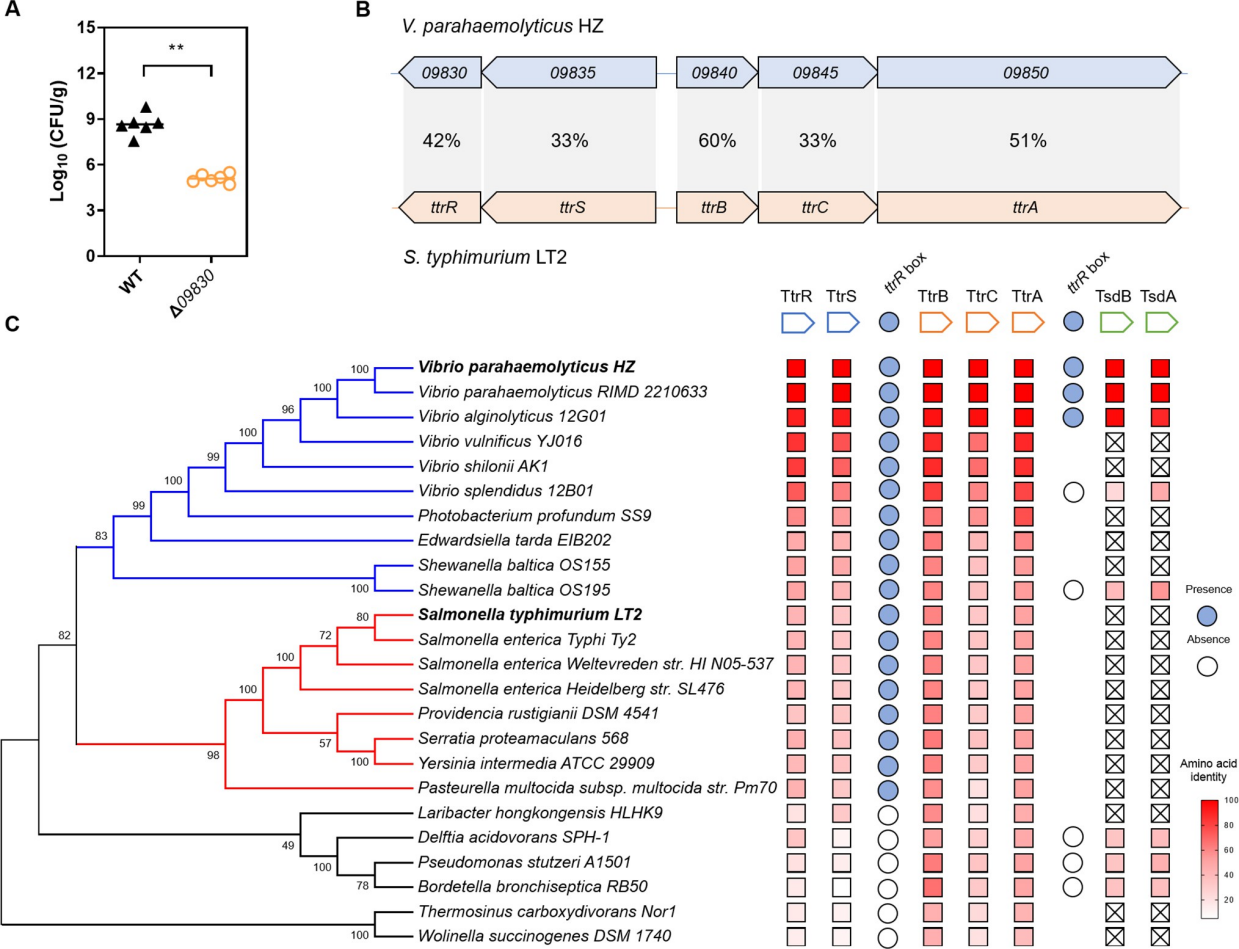
TtrRS increases the host colonization of *V. parahaemolyticus* and the *ttr* gene cluster is widely distributed in *Proteobacteria*. (**A**) Deletion of *09830* decreased the colonization of *V. parahaemolyticus*. Colonization (CFU) of *V. parahaemolyticus* was measured from feces in the streptomycin-treated adult mouse model at 48 h post-infection. Groups containing six mice each were intragastrically administered with the indicated strains at a dose of 2.0 × 10^9^ CFU/mouse. The Mann-Whitney test was used for statistical analysis, and asterisks indicate significant differences (**, *P*  < 0.01). (**B**) Homology analyses of amino acid sequences encoded by *09830–09850* genes using BLAST of NCBI. The identities of the amino acid sequences of each protein are shown between *V. parahaemolyticus* strain HZ and *S*. *typhimurium* strain LT2. (**C**) Phylogenetic analysis of Ttr homologs in prokaryotes. The phylogenetic tree was constructed with the MEGA7 software using the neighbor-joining method. The *ttrRS*-*ttrBCA*-*tsdBA* (*09830*–*09860*) gene cluster in *V. parahaemolyticus* and other bacteria strains were used for homology analyses based on amino acid sequences using BLAST. The *ttrR* box (GTGG-N_4_-CCAC) is denoted by filled circle for presence and empty circle for absence in the putative promoter region of *ttrB* and *tsdBA*.

To identify RRs required for *V. parahaemolyticus* colonization of the host gut, we constructed gene deletion mutants of the 28 predicted RRs and examined the abilities of these mutants to colonize the intestine of streptomycin-treated adult mice [20]. Six mutants exhibited a significant defect in intestinal colonization compared with that of the wild-type (WT) strain (Figs 1A and S1A). All identified mutant strains showed similar growth rates *in vitro* to that of the WT strain except Δ*01695*, which had an obvious growth defect. Further homology analysis revealed that the amino acid sequences of the 09830 protein shared 42% identity with the TtrR protein of *S*. *typhimurium*, while the adjacent gene, *09835*, shared 33% identity with the TtrS amino acid sequence (Fig 1B). The 09835 was predicted to be a typical sensor kinase harboring a conserved C-terminal catalytic and ATP-binding (CA) domain and a dimerization and histidine phosphotransfer (DHp) domain, while 09830 is composed of a DNA binding (HTH) domain and a receiver (REC) domain (S1B and S1C Fig). A similar domain architecture is commonly found in most TCSs [21], which further suggests that they constitute a homologous TtrRS TCS in *V. parahaemolyticus*.

Previous studies showed that TtrRS is required for the transcription of the *ttrBCA* operon in *S*. *typhimurium*, which forms the *ttrRS-ttrBCA* (*ttr*) gene cluster [16]. In *V. parahaemolyticus*, we found that the proteins encoded by *09840–09850*, located downstream of the *09830–09835* genes, show 33%∼60% sequence identity to the TtrBCA proteins in *S*. *typhimurium*, respectively (Fig 1B), suggesting that *V. parahaemolyticus* also possesses the *ttr* gene cluster. To estimate the generality of prokaryotes whose genomes encode the *ttr* gene cluster, we screened the National Center for Biotechnology Information (NCBI) database for orthologues of TtrRS and TtrBCA, which were further subjected to a protein sequence alignment and a phylogenetic analysis. The results showed that the *ttr* gene cluster exists widely among the *Proteobacteria*, including *Vibrio*, *Shewanella*, and *Salmonella* species (Fig 1C). We further found that TtrRS and TtrBCA are conserved in several *Vibrio* species, such as *V. parahaemolyticus*, *V. alginolyticus*, and *V. vulnificus*, whereas *V. cholerae*, *V. fischeri*, and *V. harveyi* do not possess these orthologues (Fig 1C). However, the function of the *ttr* gene cluster has not been well studied, and the molecular mechanism by which TtrRS regulates *ttrBCA* or contributes to bacterial pathogenicity also remains unclear.

### 2 TtrRS activates *ttrBCA* transcription and works as an important regulator in *V. parahaemolyticus*

**Fig 2:**
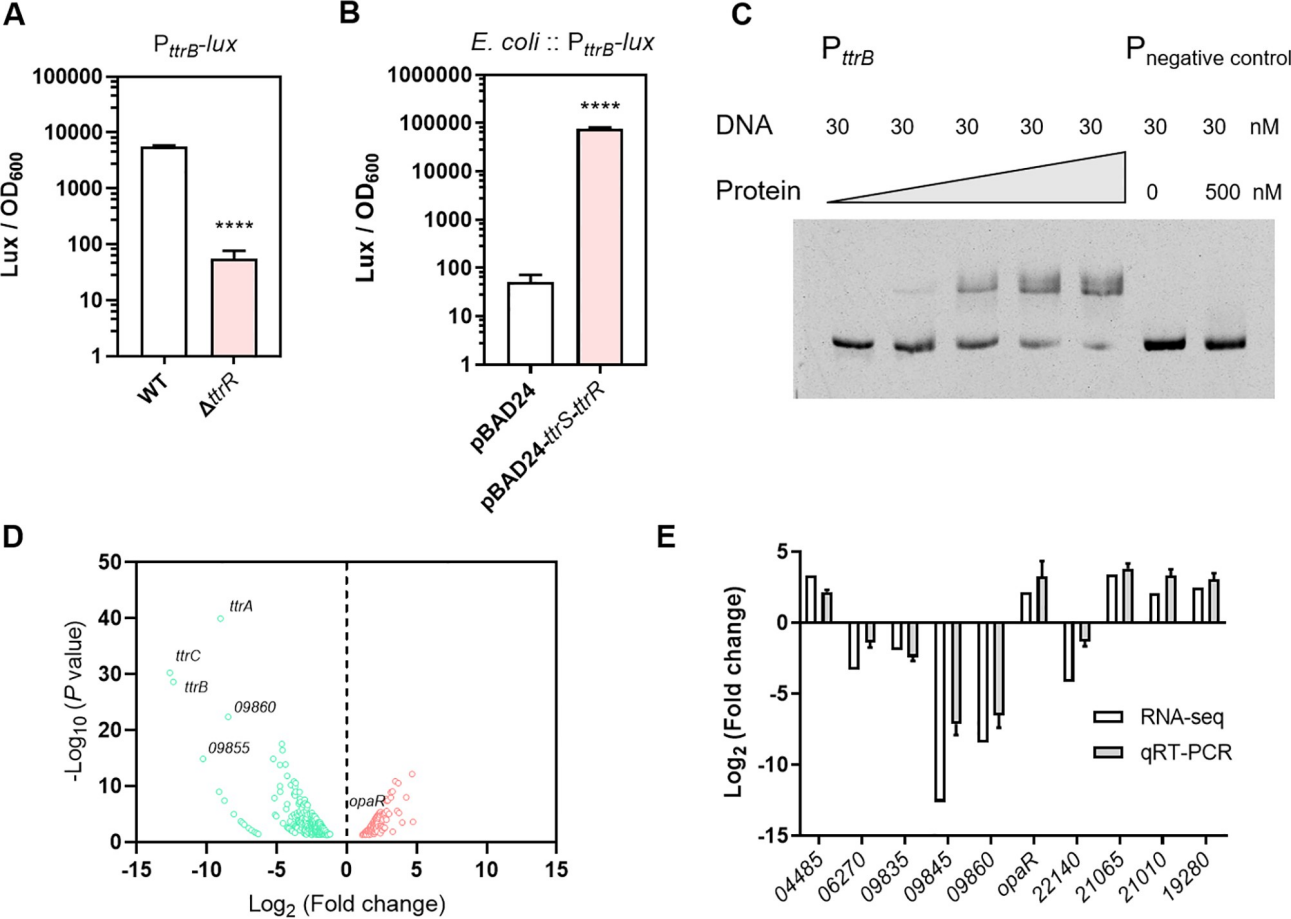
Identification of TtrSR-regulated genes. (**A**) The expression level of *ttrBCA* was assessed by measuring luminescence in P_*ttrB*_*-lux* transcriptional fusion strains. *V. parahaemolyticus* containing promoter-*lux* transcriptional fusion plasmids were grown in MLB at 37°C. (**B**) The expression level of *ttrBCA* was assessed by measuring luminescence in *E*. *coli* harboring P_*ttrB*_*-lux* reporter. The *E*. *coli* strains were grown in LB at 37°C, which also contain a pBAD24 vector control or pBAD24-*ttrS*-*ttrR*. Luminescence expression was calculated as the luminescence per unit of OD_600_. The unpaired two-tailed Student’s *t*-test was used for statistical analysis (****, *P* < 0.0001). (**C**) EMSA showing that TtrR binds to the promoter region of *ttrB*. Each reaction mixture contains DNA probe (30 nM) and TtrR protein (0 to 500 nM), and 16S rRNA served as negative control. (**D**) Volcano plot of the differentially expressed genes was analyzed between the Δ*ttrR* and WT strains by RNA-seq. The *x*-axis displays the value of log_2_ (Fold change), and the *y*-axis represents the value of -log_10_(*P* value). Red dots represent up-regulated genes, while green dots represent down-regulated genes. (**E**) Validation of gene regulation by qRT-PCR. Ten genes, including 5 upregulated genes and 5 downregulated genes identified by RNA-seq analysis, were randomly selected to perform qRT-PCR. Each sample was run in triplicate, and the housekeeping gene 16S rRNA was detected as a control.

To evaluate whether TtrRS regulates the expression of *ttrBCA* in *V. parahaemolyticus*, the promoter region of the *ttrBCA* operon was ligated with bioluminescence reporter pBBR-*lux*, which was further introduced into WT and Δ*ttrR* strains. The *V. parahaemolyticus* strains harboring the P_*ttrB*_*-lux* were cultured in fresh MLB under aerobic conditions. The results showed that the promoter activity of P_*ttrB*_*-lux* was significantly decreased (100.7-fold change) in the Δ*ttrR* strain compared with that in the WT strain (Fig 2A). By overproducing TtrRS in *Escherichia coli* from plasmid pBAD24 and measuring the activity of P_*ttrB*_*-lux* in this strain, we found that the promoter activity of P_*ttrB*_*-lux* was significantly increased (1518.1-fold change) in the *E*. *coli* containing TtrRS (Fig 2B). To determine whether this regulation is direct, electrophoretic mobility shift assays (EMSAs) were performed using the recombinant TtrR protein and the promoter region of *ttrBCA*. We found that TtrR efficiently bound to the promoter fragments of *ttrBCA*, but not the control fragment (Fig 2C). These results demonstrated that TtrR directly activates the transcription of the *ttrBCA*.

To quickly identify other potential target genes of TtrSR, we performed RNA sequencing (RNA-seq) analyses to compare the transcriptomics of WT with the Δ*ttrR* mutant in MLB broth under aerobic conditions. We found that 248 genes were significantly up-regulated and 383 genes were significantly down-regulated in the Δ*ttrR* strain relative to WT (Fig 2D and S1 Table). We randomly selected ten genes to perform qRT-PCR for further verification, and the results were consistent with the transcriptomic data (Fig 2E). To gain insight into the pathways that are regulated by TtrRS, GO categories and KEGG analysis were performed based on the differentially expressed genes. We found that several GO categories were significantly enriched in the Δ*ttrR* mutant, including transporter activity and transcription regulator activity (S2A Fig). KEGG analysis showed these genes were found to be enriched in oxidative phosphorylation, phosphotransferase system, two-component system, and sulfur metabolism (S2B Fig). In addition, we found the transcription level of *opaR*, the master quorum sensing (QS) regulator [22], was significantly increased in the Δ*ttrR* strain (Fig 2D and 2E). These results revealed that the TtrRS-regulated genes are involved in various physiological processes, and TtrRS works as an important regulator.

### 3 TtrRS exhibits positive autoregulation and precisely controls the transcription of *ttrRS*-*ttrBCA-09855-09860* gene cluster

**Fig 3:**
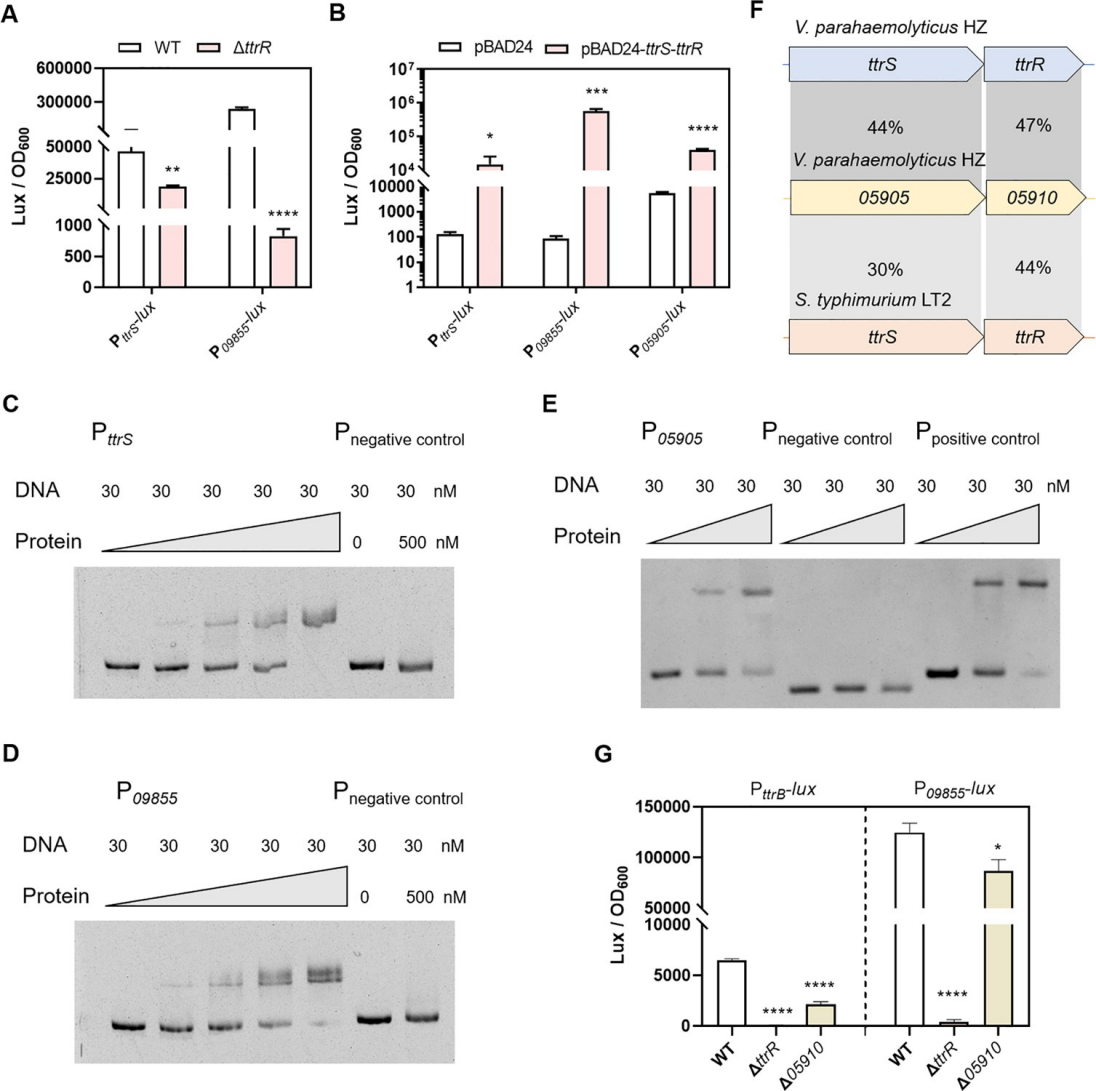
TtrRS regulates the transcription of *ttrRS*-*ttrBCA-09855-09860* gene cluster. (**A**) The expression level of *ttrRS* and *09855–09860* was assessed by measuring luminescence in P_*ttrS*_*-lux* and P_*09855*_*-lux* transcriptional fusion strains, respectively. *V. parahaemolyticus* containing promoter-*lux* transcriptional fusion plasmids were grown in MLB at 37°C. (**B**) The expression level of *ttrSR*, *09855–09860*, and *05905–05910* was assessed by measuring luminescence in *E*. *coli* harboring P_*ttrS*_*-lux*, P_*09855*_*-lux*, and P_*05905*_*-lux* reporter, respectively. The *E*. *coli* strains were grown in LB at 37°C, which also contain a pBAD24 vector control or pBAD24-*ttrS*-*ttrR*. (**C-D**) EMSA showing that TtrR binds to the promoter region of *ttrS* and *09855*, respectively. Each reaction mixture contains DNA probe (30 nM) and TtrR protein (0 to 500 nM), and 16S rRNA served as negative control. (**E**) EMSA showing that TtrR binds to the promoter region of *05905*. Each reaction mixture contains DNA probe (30 nM) and TtrR protein (0, 200, 400 nM). The 16S rRNA served as negative control, and P_*09855*_ served as positive control. (**F**) Homology analyses of amino acid sequences encoded by *05905*–*05910* genes using BLAST. The identities of the amino acid sequences of each protein are shown between *V. parahaemolyticus* strain HZ and *S*. *typhimurium* strain LT2. (**G**) The 05910 influences the transcription of TtrRS target genes. *V. parahaemolyticus* containing promoter-*lux* transcriptional fusion plasmids were grown in MLB at 37°C. Luminescence expression was calculated as the luminescence per unit of OD_600_. The unpaired two-tailed Student’s *t*-test was used for statistical analysis (*, *P* < 0.05; **, *P* < 0.01; ***, *P* < 0.001; ****, *P* < 0.0001).

TtrRS transcriptomic analysis showed that the most significantly downregulated operons were *ttrBCA*, as well as *09855–09860* (Fig 2D). We found that these two operons, together with *ttrRS*, are encoded adjacently in *V. parahaemolyticus* genomes (Fig 1C). Intriguingly, *ttrS* also showed a 3.8-fold reduction in Δ*ttrR* strain compared to the WT (S1 Table and Fig 2E). These results suggested that TtrRS specifically regulates the transcription of the *ttrRS-ttrBCA-09855-09860* gene cluster. To further investigate how TtrRS regulates the *ttrRS* and *09855–09860* operons, we constructed the promoter-*lux* fusions P_*ttrS*_*-lux* and P_*09855*_*-lux* and measured their activity in the WT and Δ*ttrR* strains cultured in MLB under the aerobic conditions. We found that the promoter activity of these fusions was all significantly decreased (2.5- and 293.3-fold change, respectively) in the Δ*ttrR* strain as compared to the WT strain (Fig 3A). By overproducing TtrRS in *E*. *coli* from plasmid pBAD24 and measuring the activity of two promoter-*lux* fusions in this strain, we found that the promoter activity of these fusions was all significantly increased (113.2- and 6845.1-fold change, respectively) in the *E*. *coli* containing TtrRS (Fig 3B), which indicated that TtrRS is essential to activate all these gene promoters. To determine whether this regulation is direct, EMSAs were performed using the recombinant TtrR protein and the promoter regions of *ttrRS* and *09855–09860*. We found that TtrR efficiently bound to the promoter fragments of all two operons, but not the control fragment (Fig 3C and 3D). These results demonstrated that TtrR has positive autoregulation and directly activates the transcription of the *09855–09860* operon.

In addition, the transcriptomic data showed that the expression of a putative TCS *05905–05910* was significantly downregulated in the Δ*ttrR* strain (S1 Table). To confirm the regulation of TtrRS to this putative TCS, we constructed the promoter-*lux* fusions P_*05905*_*-lux* and introduced it into *E*. *coli* strains. The result showed that the promoter activity of P_*05905*_*-lux* was significantly increased (7.1-fold change) in the *E*. *coli* containing TtrRS (Fig 3B). By using EMSAs, we further found that TtrR could directly regulate the expression of *05905–05910* (Fig 3E). We then attempt to explore the potential relationship between the two TCSs. Intriguingly, although the function of 05905–05910 has remained uncharacterized, homology analysis showed that their amino acid sequences shared 44% and 47% identity with 09835 and 09830, while 30% and 44% identity with TtrS and TtrR of *S*. *typhimurium* (Fig 3F). We assessed whether 05905–05910 affects the transcription of *ttrBCA* and *tsdBA* by measuring the activity of promoter-*lux* fusion in the deletion strains. Notably, 05905–05910 increased the transcription (3.0- and 1.4-fold change, respectively) of *ttrBCA* and *09855–09860*, while TtrRS is absolutely required for the transcription of the two operons (Fig 3G). Taken together, the data suggested that 05905–05910 TCS may assist in the regulation of TtrRS and thus TtrRS could more precisely control the transcription of the *ttrRS-ttrBCA-09855-09860* gene cluster.

### 4 TtrR activates target gene expression by directly binding to the *ttrR* box

**Fig 4:**
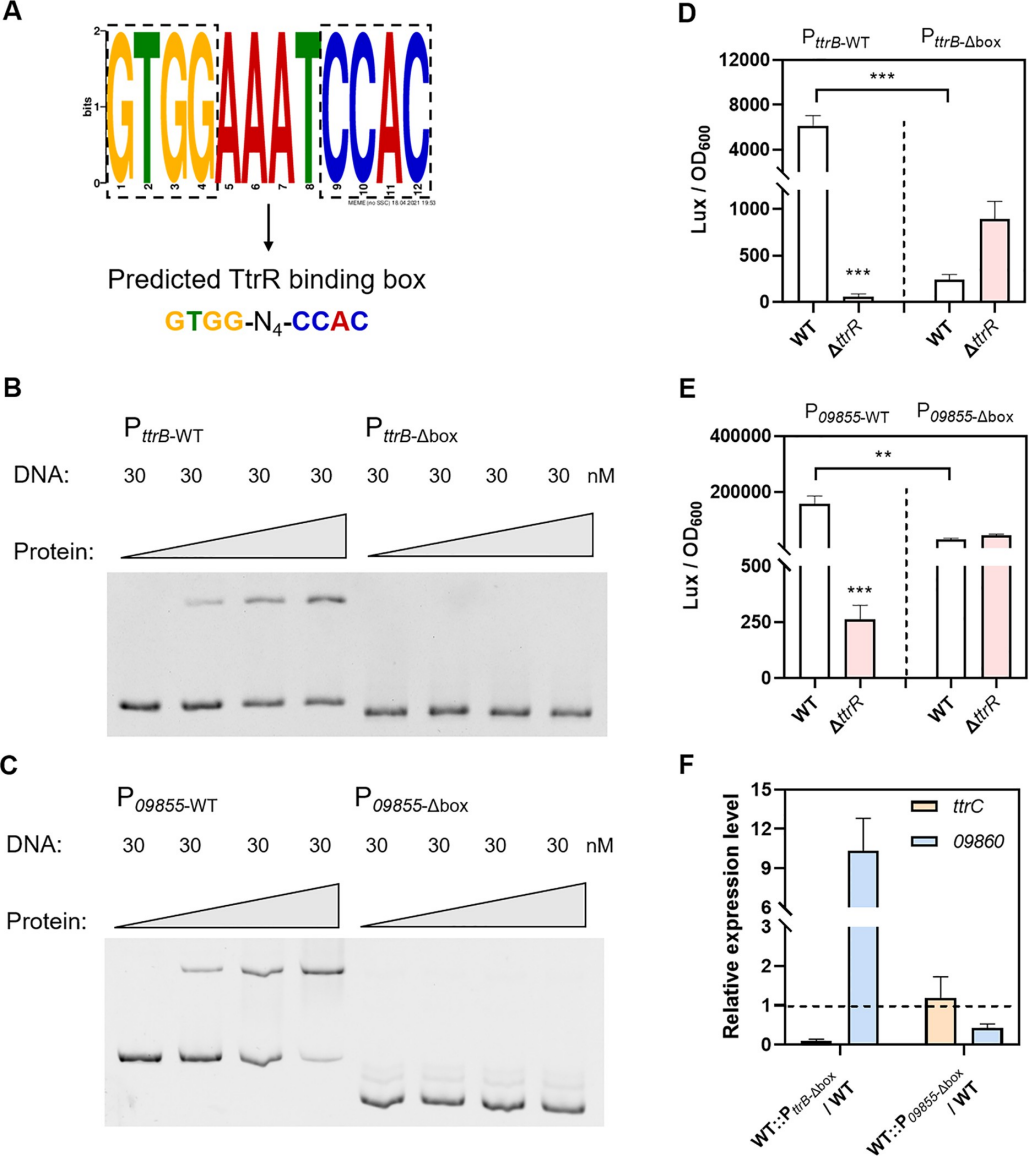
Verification of the TtrR binding sites. (**A**) WebLogo generated from the alignment of binding sequences to show the TtrR binding box. N stands for any nucleotide. (**B-C**) Validation of TtrR binding to the box in the promoter regions by EMSA. The triangle indicates the protein concentration gradient, which was 0 nM, 200 nM, 300 nM, and 400 nM. (**D-E**) Validation of TtrR binding to the box in the promoter regions by bioluminescence reporter assay. *V. parahaemolyticus* containing promoter-*lux* transcriptional fusion plasmids were grown in MLB at 37°C. Luminescence expression was calculated as the luminescence per unit of OD_600_. (**F**) mRNA level of *ttrC* and *09860* in WT and box deleted strain was determined by using qRT-PCR. The results are expressed as means ± SD from three independent experiments. The unpaired two-tailed Student’s *t*-test was used for statistical analysis (**, *P* < 0.01; ***, *P* < 0.001).

To identify the consensus TtrR binding motif, we used MEME Suite to analyze the promoter regions of *ttrRS*, *ttrBCA*, and *tsdBA*. As shown in Figs 4A and S3, a typical inverted repeat (GTGG-N_4_-CCAC) which was named as *ttrR* box was identified immediately upstream of the -35 box in the three promoter regions. To verify the *ttrR* box experimentally, the promoter variants harboring the *ttrR* box deletion were constructed and analyzed using EMSAs. The results showed that the promoters without the *ttrR* box were unable to form complexes with TtrR (Fig 4B and 4C). To validate the necessity of the *ttrR* box for gene regulation, the *ttrR* box deletion was made in the previous promoter-*lux* fusions and expression studies were performed in WT and Δ*ttrR* strains cultured in MLB under the aerobic conditions. As shown in Fig 4D and 4E, deletion of the *ttrR* box made the promoter activity significantly decreased (25.2- and 5.0-fold change, respectively) in the WT strain and eliminated the positive regulation of the target genes by TtrR. Meanwhile, we observed that the promoter activity of the promoter-*lux* fusions was increased in the Δ*ttrR* strain when the *ttrR* box was deleted from the regulatory regions, which may be due to the alteration of the promoters’ structure. We also deleted the *ttrR* box from the *ttrBCA* or *09855-09860* promoters in the WT strain respectively and further confirmed that the expression level of *ttrC* or *09860* in the *ttrR* box-deleted strain was significantly decreased (10.0- and 2.3-fold change, respectively) compared to that of the WT strain by using qRT-PCR assay (Fig 4F). These results demonstrated that TtrR activates target gene expression by directly binding to the *ttrR* box in *V. parahaemolyticus*. In addition, bioinformatics analysis revealed that the *ttrR* box often cooccurrences with the *ttr* gene cluster across bacterial genomes (Fig 1C), which suggested that the *ttrR* box possesses high conservatism during species evolution.

### 5 TtrRS activates target gene expression by sensing tetrathionate

**Fig 5:**
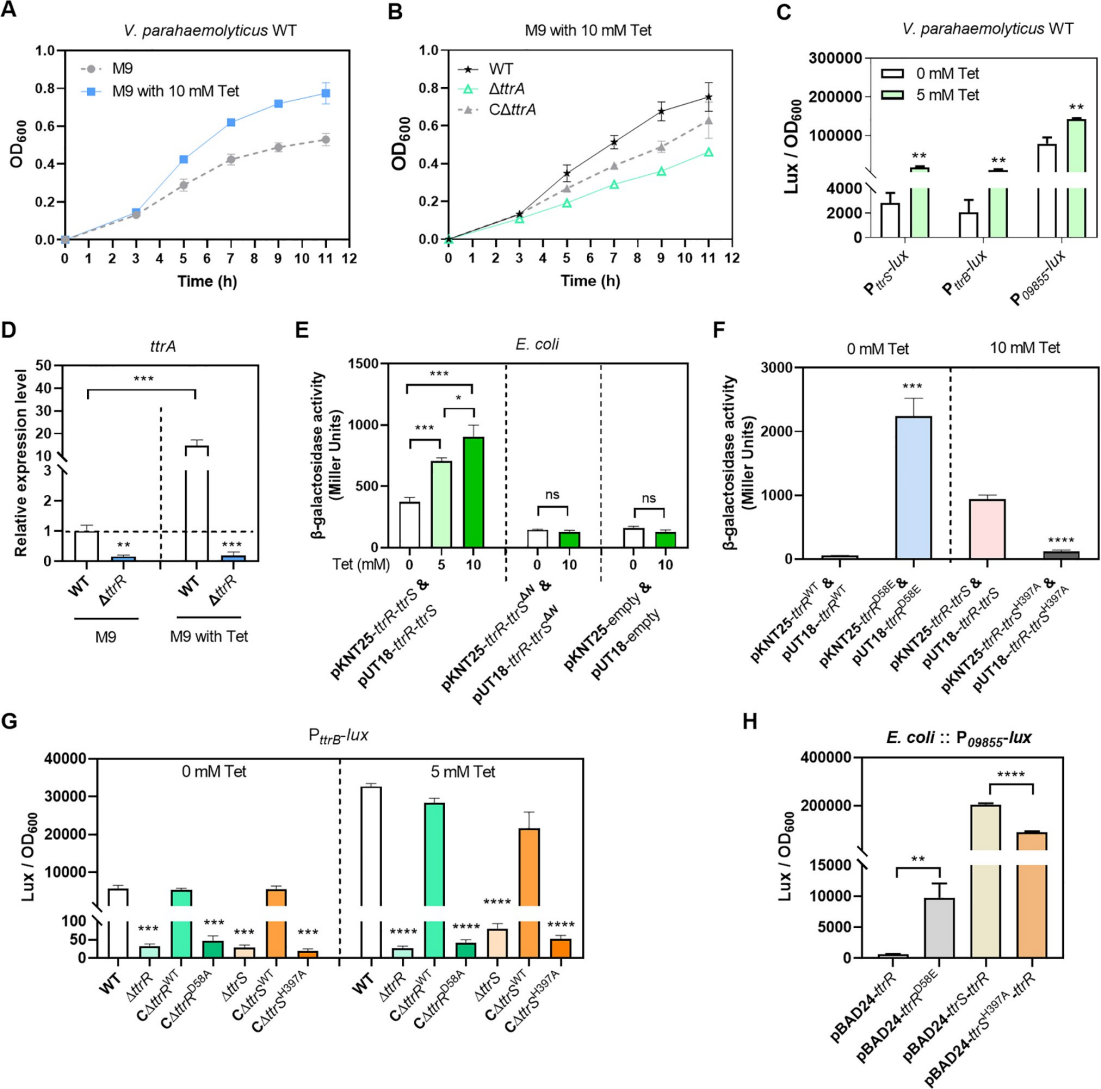
Tetrathionate triggers TtrRS phosphorylation and increases the expression of target genes. (**A**) Tetrathionate supports the growth of *V. parahaemolyticus*. (**B**) Deletion of *ttrA* reduces bacterial growth in the presence of tetrathionate. The strains were grown in M9 in the absence or in the presence of tetrathionate at 37°C under micro-aerobic conditions. Bacteria cell density was measured and reported as the value of OD_600_. (**C**) The effect of tetrathionate on transcription of TtrRS target genes. *V. parahaemolyticus* containing promoter-*lux* transcriptional fusion plasmids were grown in M9 in the absence or the presence of tetrathionate at 37°C. Luminescence expression was calculated as the luminescence per unit of OD_600_. (**D**) Tetrathionate induces the transcription of TtrRS target genes. mRNA level of *ttrA* in WT and Δ*ttrR* strains was determined by using qRT-PCR. The results are expressed as means ± SD from three independent experiments. (**E-F**) Tetrathionate and phosphorylation promote TtrR dimerization. The recombinant pKNT25 and pUT18 plasmids were co-transformed into *E*. *coli* BTH101. The strains were grown in M9 in the absence or the presence of tetrathionate at 30°C for 8 h. The β-galactosidase activity was measured and reported as Miller Units. (**G**) Mutation of TtrR Asp58 or TtrS His397 abolished the transcription of TtrRS target genes. The Asp58 of TtrR or His397 of TtrS was substituted with alanine on the genome of *V. parahaemolyticus*. The strains containing P_*ttrB*_*-lux* plasmid were grown in M9 in the absence or the presence of tetrathionate at 37°C. (**H**) Mutation of phosphorylation sites influences the TtrRS activity. The *E*. *coli* strain contains both a P_*09855*_*-lux* and a recombinant pBAD24 plasmid. Luminescence expression was calculated as the luminescence per unit of OD_600_. The unpaired two-tailed Student’s *t*-test was used for statistical analysis (ns, *P* > 0.05; *, *P* < 0.05; **, *P* < 0.01; ***, *P* < 0.001; ****, *P* < 0.0001).

In *S*. *typhimurium*, TtrBCA confers the ability to respire with tetrathionate as an electron acceptor [19]. Here, we found that tetrathionate supported the growth of *V. parahaemolyticus* under the micro-aerobic environment (Fig 5A), while deletion of *ttrA* significantly reduced the bacterial growth, and this deficiency was restored by complementation (Fig 5B). Because the *ttr* gene cluster plays a critical role in tetrathionate utilization, we reasoned that the TtrS is a tetrathionate sensor and that the phosphorylated TtrR activates *ttrBCA* transcription. To test this hypothesis, we performed the qRT-PCR and promoter-*lux* fusions assays to analyze the expression of the target genes when the WT strain was grown in the culture with or without tetrathionate under the micro-aerobic conditions. Here we observed that tetrathionate induces the expression of target genes in *V. parahaemolyticus* WT strain (Fig 5C and 5D). Then, we tested whether tetrathionate has the ability to induce the homodimerization of TtrR which is a common regulatory theme among TCSs [11,12]. By applying the bacterial two-hybrid system, we found that the homodimerization of TtrR was controlled by tetrathionate in a dose-dependent manner, while the deletion of the N-terminal domain of TtrS abolished the homodimerization of TtrR (Fig 5E). These indicated that tetrathionate could induce the homodimerization of TtrR via TtrS to activate the *ttrRS-ttrBCA-09855-09860* gene cluster.

Previous studies reported that the phosphorylated histidine (His) of HKs is located within a conserved sequence motif on the α1-helix of the DHp domain, while the phosphorylated aspartate (Asp) of RRs is located in the loop following the β3-sheet of the REC domain [11,21]. Here we identified that His397 of TtrS and Asp58 of TtrR were highly conserved residues and within the proper site of DHp and REC domain, respectively, by using multiple sequence alignment and secondary structure prediction programs (S4A and S4B Fig). We confirmed that the homodimerization of TtrR was controlled by Asp58 of TtrR and His397 of TtrS in the presence of tetrathionate using the bacterial two-hybrid system (Fig 5F). To further validate the signal transduction of TtrRS and assess its effect on gene regulation, we substituted the conserved phosphorylation site (His397 of TtrS, and Asp58 of TtrR) with alanine on the genome of *V. parahaemolyticus*, and measured the activity of the promoter-*lux* fusion in these strains cultured in M9 broth under the micro-aerobic conditions. We showed that deletion of *ttrR* or *ttrS* on the genome abolished the activity of the *lux* fusion, and complementation of Δ*ttrR* with a WT *ttrR* gene or Δ*ttrS* with a WT *ttrS* gene could both restore the activity of the *lux* fusion (Fig 5G). However, D58A or H397A substitutions on the genome completely eliminated the activity of the *lux* fusion (Fig 5G). Similar phenotypes were observed in the *E*. *coli* strain, which contains the promoter-*lux* fusion and a pBAD24 plasmid overproducing TtrRS or their variants (Figs 5H and S4C). Our results indicated that tetrathionate and its triggered phosphorylation between TtrS and TtrR are essential for the TtrRS regulation to transcription of the target genes.

### 6 TtrRS regulates sulfur metabolism

**Fig 6:**
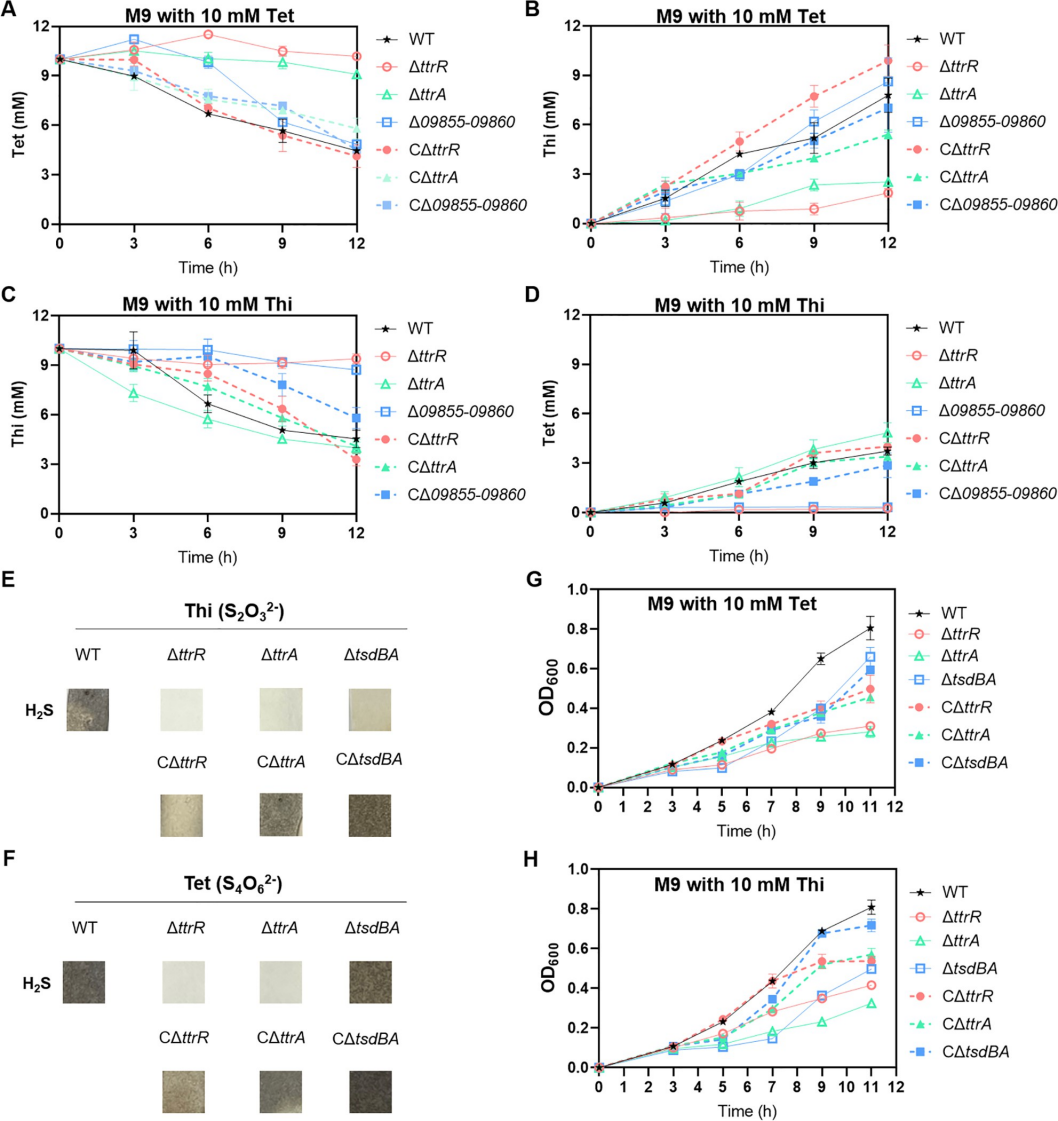
TtrSR and their target genes participate in sulfur metabolism in *V. parahaemolyticus*. (**A-B**) Comparison of tetrathionate reduction and thiosulfate generation in the cultures of WT and the relevant mutants. The strains were grown in a modified M9 minimal medium containing sodium tetrathionate under micro-aerobic conditions. The complemented strains harbor a recombinant pBAD24 carrying the relevant deleted gene, while other strains have an empty pBAD24 plasmid. Experiments were repeated at least three times. (**C-D**) Comparison of thiosulfate oxidation and tetrathionate generation in the cultures of WT and the relevant mutants. The strains were grown in a modified M9 minimal medium containing sodium thiosulfate under micro-aerobic conditions. (**E-F**) H_2_S generation by WT and the relevant mutants. The strains were grown in a modified M9 minimal medium containing sodium thiosulfate or sodium tetrathionate. H_2_S generation was monitored by lead acetate strips. (**G-H**) Growth curves of WT and the relevant mutants. The strains were grown in M9 in the presence of tetrathionate or thiosulfate at 37°C under micro-aerobic conditions. Bacteria cell density was measured and reported as the value of OD_600_.

We found that *09855–09860* encodes a putative cytochrome *c*, and homology analysis revealed that their amino acid sequences shared 40% and 61% identity with the TsdB and TsdA proteins of *Shewanella oneidensis* (S5A Fig), respectively, which function primarily as a thiosulfate dehydrogenase and carries out thiosulfate oxidation [23]. Bioinformatics analysis revealed that the *tsdBA* operon was not always cooccurrence with the *ttr* gene cluster across bacterial genomes (Fig 1C). Although the above result showed that TtrBCA promotes *V. parahaemolyticus* growth by controlling tetrathionate utilization (Fig 5B), the mechanism by which the bacteria perform tetrathionate reduction or thiosulfate oxidation needs further investigation. To investigate the functions of TtrRS target genes, *ttrBCA* and *09855–09860*, on sulfur metabolism, we grew *V. parahaemolyticus* in an M9 medium with tetrathionate or thiosulfate and monitored the level of the sulfur compounds by using high-performance liquid chromatography (HPLC). As expected, when grown in an M9 medium containing 10 mM sodium tetrathionate under micro-aerobic conditions, the WT strain was able to consume tetrathionate and produce thiosulfate simultaneously, whereas the Δ*ttrR* in which *ttrBCA* is not activated and Δ*ttrA* strains failed to consume tetrathionate (Fig 6A and 6B). Genetic complementation of these mutants restored the ability to reduce tetrathionate (Fig 6A and 6B), confirming that *ttrBCA* is essential for tetrathionate reduction. Furthermore, we found that thiosulfate metabolism contributes to the growth phenotype of WT strain under micro-aerobic conditions (S5B Fig). Our HPLC results further showed that both the WT and Δ*ttrA* were able to consume thiosulfate and produce tetrathionate, however, the Δ*ttrR* strain in which *09855–09860* is not activated and Δ*09855–09860* strains failed to consume thiosulfate (Fig 6C and 6D). Genetic complementation of these mutants restored the ability to oxidize thiosulfate (Fig 6C and 6D), demonstrating that *09855–09860* is responsible for thiosulfate oxidation functioning as TsdBA of *S*. *oneidensis* [23], and thus they were redesignated as *tsdBA* in *V. parahaemolyticus*.

Thiosulfate serves as a respiratory electron acceptor for diverse microorganisms, which releases H_2_S as one of the end products [24,25]. Here we monitored H_2_S generation by *V. parahaemolyticus* WT and the relative mutant strains during micro-aerobic culture in M9 medium with thiosulfate. We found that the H_2_S level produced by Δ*ttrR*, Δ*ttrA*, and Δ*tsdBA* strains were all decreased compared with that of the WT strain, especially the Δ*ttrA* strain, which failed to produce H_2_S to levels detectable by using lead acetate paper strips (Fig 6E). In addition, complementation restored all the strains’ ability to produce H_2_S (Fig 6E). Similar results were observed when the strains were cultured in M9 medium with tetrathionate except Δ*tsdBA* (Fig 6F), which retains the ability of sulfur compound reduction. We next investigated whether the sulfur compounds utilization supports the growth of *V. parahaemolyticus*, and the results showed that both the Δ*ttrR*, Δ*ttrA*, and Δ*tsdBA* strains displayed statistically significant growth defects as compared to the WT (Fig 6G and 6H). Collectively, these results strongly suggest that in *V. parahaemolyticus*, TtrBCA is essential for the reduction of tetrathionate and thiosulfate, and TsdBA is required for the oxidation of thiosulfate. TtrRS controls the reduction of tetrathionate and thiosulfate via regulating the expression of *ttrBCA* and controls the oxidation of thiosulfate via regulating the expression of *tsdBA* in *V. parahaemolyticus*.

### 7 TtrRS and their target genes constitute a tetrathionate-responsive genetic circuit that contributes to intestinal colonization of *V. parahaemolyticus*

**Fig 7:**
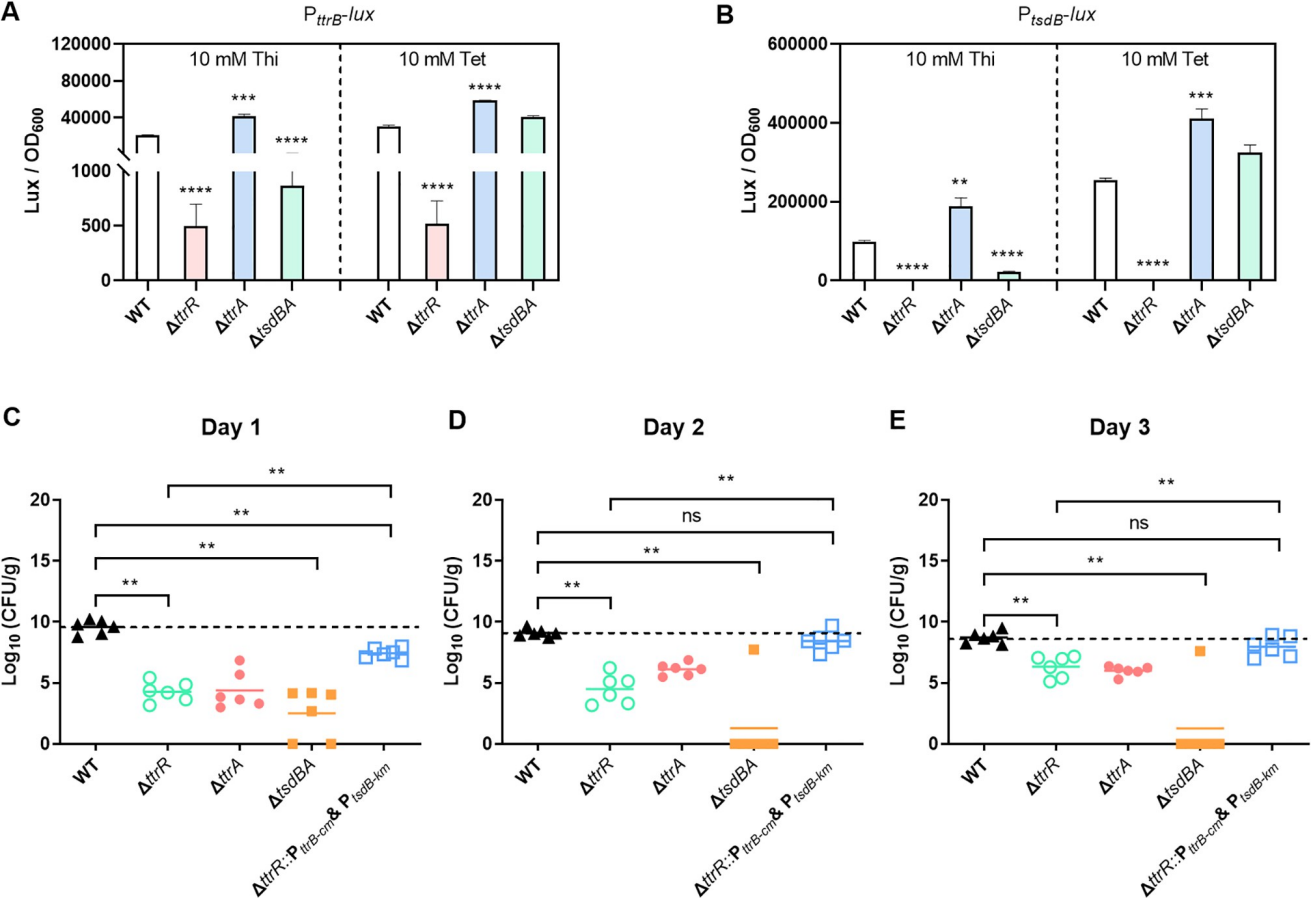
Target genes influence TtrRS regulation and *V. parahaemolyticus* colonization. (**A-B**) Deletion of *ttrA* or *tsdBA* affects the transcription of TtrRS target genes. *V. parahaemolyticus* containing promoter-*lux* transcriptional fusion plasmids were grown in a modified M9 minimal medium, which contained sodium thiosulfate or sodium tetrathionate at 37°C. Luminescence expression was calculated as the luminescence per unit of OD_600_. (**C-E**) TtrSR and their target genes contribute to intestinal colonization of *V. parahaemolyticus*. Colonization (CFU) of *V. parahaemolyticus* was measured from feces in the streptomycin-treated adult mouse model. Groups containing six mice each were intragastrically administered with the indicated strains at a dose of 2.0 × 10^9^ CFU/mouse. The unpaired two-tailed Student’s *t*-test (A-B) or Mann-Whitney test (C-E) was used for statistical analysis (ns, *P* > 0.05; **, *P* < 0.01; ***, *P* < 0.001; ****, *P* < 0.0001).

As the target genes of TtrRS were found to be involved in sulfur metabolism (Fig 6), we then assessed if these genes affect the regulation of TtrRS by measuring the activity of promoter-*lux* fusion in appropriate deletion strains cultured in M9 broth under the micro-aerobic conditions. The activity of *lux* fusion was significantly increased (1.9- and 1.6-fold change, respectively) when *ttrA* was deleted from the genome of the WT strain (Fig 7A and 7B). Similar results were observed in Fig 4F, which showed that the expression level of *tsdA* (*09860*) was significantly increased (10.3-fold change) when *ttrR* box was deleted from the *ttrBCA* promoter in the WT strain. This was probably caused by the increasing tetrathionate in the absence of TtrA which could reduce tetrathionate. However, in the presence of exogenous thiosulfate, the activity of *lux* fusion in Δ*tsdBA* was significantly decreased (23.8- and 4.5-fold change, respectively) compared with that of the WT strain (Fig 7A and 7B), which indicated that the oxidation of thiosulfate by TsdBA plays an essential role in TtrRS activating *ttrBCA* or *tsdBA* expression. Considering the positive autoregulation of TtrRS, these findings strongly suggest that TtrRS and their target genes constitute a genetic circuit to respond to environmental sulfur.

To investigate whether TtrRS and their target genes participate in the virulence of *V. parahaemolyticus*, the streptomycin-treated mouse model was utilized and the colonization was examined by measuring the bacterial loads in fecal samples. As shown in Fig 7C–7E, the bacterial loads in the samples from the Δ*ttrR*, Δ*ttrA*, and Δ*tsdBA* strains-infected mice were all significantly reduced compared to that of the WT strain, which suggested that these mutants were less fit in the gut environment. To determine whether the defect of colonization of Δ*ttrR* was caused by its failing to activate TtrBCA and TsdBA, we then replaced the *ttrBCA* or *tsdBA* promoters in Δ*ttrR* strain with a constitutively expressed promoter, respectively, which were confirmed by using qRT-PCR (S6A Fig). We then monitored tetrathionate reduction, and the results showed that Δ*ttrR* strain with P_*ttrB-cm*_ restored the ability to consume tetrathionate and produce H_2_S (S6B Fig). These results suggested that the replaced promoters, P_*ttrB-cm*_ and P_*tsdB-km*_, were out of TtrRS control and could sustainably express the *ttrBCA* or *tsdBA* in Δ*ttrR* strain. Meanwhile, we found that Δ*ttrR* strain with the exogenous promoters P_*ttrB-cm*_ and P_*tsdB-km*_ restored the ability to colonize in the gut of the mice (Fig 7C–7E). In addition, results from additional mouse infection assays using the *ttrR* box-deleted strains showed that the bacterial loads in the samples from the *ttrR* box-deleted strain-infected mice were significantly reduced compared to that with the WT strain (S6C Fig). Taken together, these findings suggest that the TtrRS genetic circuit involved in sulfur metabolism is critical for the host colonization of *V. parahaemolyticus*.

## Discussion

**Fig 8:**
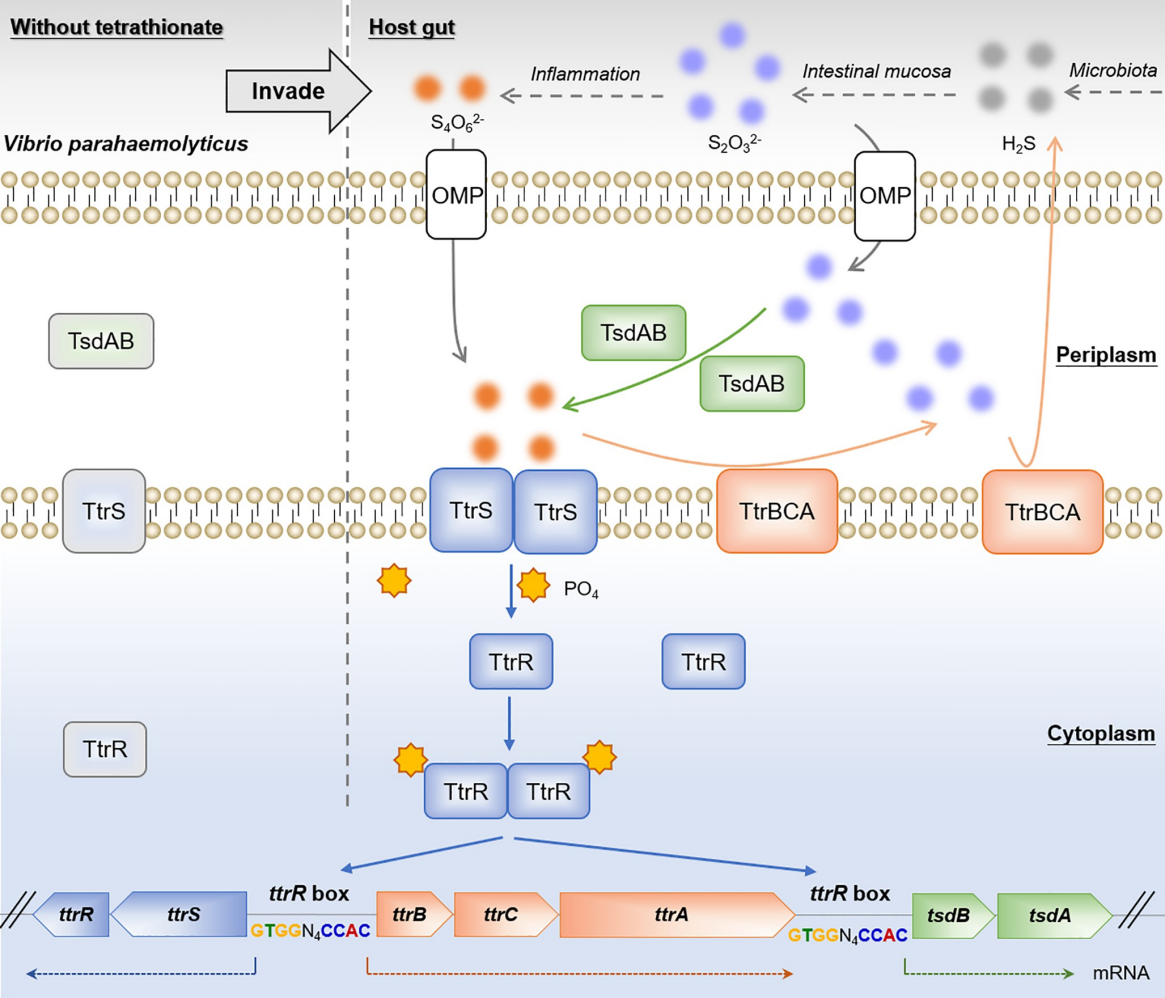
Proposed model of TtrRS-mediated tetrathionate-responsive genetic circuit that contributes to intestinal colonization of *V. parahaemolyticus*. Gut microbiota produces large quantities of H_2_S, while the intestinal mucosa could convert it to thiosulfate (S_2_O_3_^2-^) [17, 18]. Once inside the host gut, *V. parahaemolyticus* reacts with thiosulfate to form tetrathionate (S_4_O_6_^2-^) by TsdBA. *V. parahaemolyticus* then senses the presence of tetrathionate through TtrS, which, in turn, transphosphorylates TtrR; phosphorylated TtrR is an active dimer form and therefore binds to the *ttrR* box of the promoter region of *ttrRS*-*ttrBCA-tsdBA* gene cluster and upregulates their transcription, which enhances TsdBA to oxidize thiosulfate to tetrathionate that further activates TtrRS. Meanwhile, the increased TtrBCA functions to conduce the reduction of both tetrathionate and thiosulfate, which promotes bacterial growth and colonization in the host gut. Therefore, TtrRS and their target genes constituted a tetrathionate-responsive genetic circuit to sufficiently exploit the host available sulfur compounds, which further contributes to the intestinal colonization of *V. parahaemolyticus*.

One of the greatest challenges encountered by pathogenic bacteria is responding to rapid changes of nutrient availability in the host [4]. TCSs represent a major mechanism through which bacterial cells sense and utilize available nutrient sources associated with particular niches [11]. In this study, we identified a TCS, TtrRS, which senses environmental tetrathionate and subsequently activates the transcription of the *ttrRS*-*ttrBCA-tsdBA* gene cluster. We demonstrated that *tsdBA* confers the ability to oxidize thiosulfate to continuously activate the TCS TtrRS, while *ttrBCA* confers the ability to reduce sulfur, which both promotes the bacterial growth under the sulfur condition and contributes to intestinal colonization of *V. parahaemolyticus*. On the basis of these data, we propose a model in which TtrRS constitutes a tetrathionate-responsive genetic circuit with their target genes and plays a critical role in the regulation of intestinal colonization (Fig 8).

*V. parahaemolyticus* is a leading cause of acute gastroenteritis, which is often accompanied by self-limited watery diarrhea, abdominal pain, nausea, and vomiting [1]. As an intestinal pathogen, successful colonization in the host intestine is important for *V. parahaemolyticus* at the initial infection stage. *V. parahaemolyticus* relies on hemagglutinin, enolase, and a type VI secretion system (T6SS) to adhere to host tissues, and then the bacterial cells produce different types of toxins important for the invasion and proliferation during the infection process, such as T3SS1 and T3SS2 [3,6,26–30]. Although these virulence factors have improved the understanding of the mechanism of *V. parahaemolyticus* infection, little is known about how the bacterium survives within the host gastrointestinal tract. In this study, we found that the deletion of *ttrR*, a RR gene of the TCS TtrRS, significantly attenuated the intestinal colonization of *V. parahaemolyticus* in the streptomycin-treated adult mouse model (Fig 1). Similar results were observed in the pandemic *V. parahaemolyticus* strain RIMD 2210633 (S7 Fig), an O3:K6 serotype associated with worldwide outbreaks of food-borne gastroenteritis [1,31]. These results suggested that TtrRS plays an important role in helping *V. parahaemolyticus* persist and colonize in the host gut. RNA-seq analyses identified 631 genes in the Δ*ttrR* strain with significantly different transcription levels compared with those in the WT strain, which helped elucidate the underlying regulatory mechanism of TtrRS in *V. parahaemolyticus*. For instance, we observed that TtrRS regulates the transcription of the master QS regulator OpaR. The QS is a cell-to-cell communication system by which bacteria sense changes in local cell density to coordinate with each other, which regulates hundreds of genes in *Vibrio*, including the virulence factors T3SS and T6SS of *V. parahaemolyticus* [8,32]. Our transcriptomic data showed that many genes related to the T3SS and T6SS have altered expression (S1 Table). However, the *ttrR* box is not present in the promoter region of these genes (S8 Fig), suggesting an indirect regulation by TtrRS possibly via OpaR. Previous studies indicated that sulfate and thiosulfate are taken up by membrane transporters called sulfate permeases, which include ATP-binding cassette (ABC)-type transporters [33]. Here we observed that the expression of many membrane transporters, such as multiple porins, RND pumps, as well as ABC-type transporters, were apparently changed in the transcriptomic data (S1 Table). Considering the *ttrR* box is not present in the promoter region of these genes (S8 Fig), it is possible that the transport of sulfur compounds was also indirectly regulated by TtrRS. These findings suggested that TtrRS works as an important regulator and facilitates the host colonization of *V. parahaemolyticus*.

Of these 631 genes in the transcriptomic dataset, we found that the most significantly downregulated operons were *ttrBCA* and *tsdBA* (Fig 2). *S*. *typhimurium* uses T3SSs to trigger acute intestinal inflammation, which oxidizes endogenous thiosulfate to form the new respiratory electron acceptor, tetrathionate [19,34]. TtrBCA confers the ability to respire with tetrathionate as an electron acceptor and allows *S*. *typhimurium* to compete with gut microbes lacking this capacity [19,34]. Therefore, TtrBCA was considered as a tetrathionate reductase. Here, we demonstrated that TtrBCA could catalyze the reduction of tetrathionate in *V. parahaemolyticus*, which is similar to its roles in *S*. *typhimurium*. Notably, a thiosulfate reductase encoded by the *phsABC* operon has been well characterized in *S*. *typhimurium*, which could reduce thiosulfate to sulfite plus H_2_S [35,36]. Although thiosulfate is more abundant in host gut than tetrathionate, the role that thiosulfate or thiosulfate reductases including PhsABC plays in the infection of *S*. *typhimurium* or other pathogenic bacteria remains unclear. We found that the homologous protein of PhsABC was not encoded in the *V. parahaemolyticus* genome. Intriguingly, by using HPLC and lead acetate detection, we demonstrated that TtrBCA could sequentially catalyze the reduction of tetrathionate and thiosulfate and finally releases H_2_S into the surroundings, which provides *V. parahaemolyticus* with a growth and colonization advantage in the gut. The results suggested that TtrBCA is not only a tetrathionate reductase but also a thiosulfate reductase in *V. parahaemolyticus*, and the thiosulfate reductase contributes to the bacterial pathogenicity. The thiosulfate dehydrogenase (Tsd) system containing TsdA and TsdB is a widespread and well-studied system, which capable of catalyzing thiosulfate oxidation [23,25,37]. Both TsdA and TsdB are functional di-heme cytochrome *c* subunits, and they function as the catalytic subunit and the electron acceptor partner respectively [25,38]. Nevertheless, their potential roles in bacterial pathogenicity are not clear. Here, we demonstrated that TsdBA confers on *V. parahaemolyticus* the ability to oxidize thiosulfate to tetrathionate and further contributes to the bacterial colonization in host gut (Figs 6 and 7). However, bioinformatics analysis revealed that the *S*. *typhimurium* genome does not encode TsdBA homologs and the *tsdBA* operon was not always cooccurrence with the *ttr* gene cluster across bacterial genomes (Fig 1C). These results suggested that *tsdBA*-mediated bacterial colonization is species-specific. Our results not only dissect the biochemical mechanism of TtrBCA involved in sulfur reduction but also indicate the potential significance of thiosulfate oxidation by TsdBA in mammalian infection.

To date, studies about the regulators govern cellular responses to sulfide levels are mainly involved in the protein persulfidation by sulfide [25,39,40]. How bacteria, especially intestinal pathogens, regulate the bioavailability of utilizable sulfur in the host is poorly understood. In the present study, we demonstrated that TtrRS could sense external tetrathionate and positively regulate the transcription of the *ttrBCA* and *tsdBA* by directly binding to the *ttrR* box, which further activates the bacterial ability to utilize the environmental sulfur compounds. By using the Pfam website and BLAST, we found that the phosphonate-bd domain, which shows similarity to the *E*. *coli* PhnD, is present at the N-terminal of TtrS. PhnD is homologous to sulfate- and phosphate-binding proteins and is involved in active transport of alkylphosphonates across the inner membrane [41,42]. Given the important role of N-terminal domain of TtrS in the homodimerization of TtrR (Fig 5E), we reasoned that TtrS relies on the phosphonate-bd domain to sense the environmental tetrathionate and further triggered phosphorylation between TtrS and TtrR. It’s worth noting that the deletion of the N-terminal domain of TtrS or the mutation of the conserved phosphorylation site of TtrS might affect the protein stability which may result in low protein abundance and thus fail to promote the homodimerization of TtrR. We also found that the two TCSs, TtrRS and 05905–05910, show coregulation to the same target genes (Fig 3). TtrRS and 05905–05910 are phylogenetically very close to each other, with a 44% identity between HKs, and 47% identity between RRs. Therefore, 05905 has a similar modular architecture to TtrS with a phosphonate-bd domain linked to a conserved catalytic core, and both the TtrR and 05910 have similar DNA binding domains. It has been proposed that the functional cross-talk between two TCSs is possible [43]. For instance, the TCSs NarX/NarL and NarQ/NarP have cross-phosphotransfer in *E*. *coli* [44], and NarL and NarP could bind to the same sites of target genes but with different affinities [45]. Considering the direct regulation of *05905–05910* by TtrRS (Fig 3E), these findings suggest that a potential cross-talk between the two TCSs may enable *V. parahaemolyticus* to integrate information from different sources of the host gut and more precisely control the transcription of *ttrRS-ttrBCA-tsdBA* gene cluster.

Previous studies reported that tetrathionate causes a slight induction of *ttrBCA* operon under aerobic conditions, but its major inductive effect occurs anaerobically, while the global anaerobic metabolism regulator FNR mutation eliminated the anaerobic induction by tetrathionate [46]. *S*. *typhimurium* strain carrying an *fnr* mutation was unable to produce tetrathionate reductase activity or respire tetrathionate [16]. Therefore, the expression of an active tetrathionate reduction system is repressed by O_2_ and requires FNR in *S*. *typhimurium*. Similar results were observed in *Shewanella baltica*, which showed that FNR plays a role in TtrSR activation [47]. However, here our results showed that tetrathionate could cause a significant induction of *ttrBCA* operon via TtrRS under both aerobic and micro-aerobic conditions. In addition, we found that TtrBCA retains the ability to catalyze the reduction of tetrathionate and thiosulfate under aerobic conditions and finally release H_2_S by using lead acetate detection. It seems that the promoter activity of the *ttr* gene cluster is free from the anaerobic-dependent regulation by FNR in *V. parahaemolyticus*. Given that *S*. *typhimurium* relies on the host intestinal inflammation to produce tetrathionate and activate the TtrRS, it makes sense that TtrBCA carries out tetrathionate reduction requires the anaerobic environments and oxygen sensor regulator FNR in *S*. *typhimurium*. In contrast, our results showed that TtrRS executes the regulation of the target genes regardless of whether the oxygen or exogenous tetrathionate is present or not (Figs 2A, 3A and 5G). We speculate that this is attributed to the TsdBA system, which endows with *V. parahaemolyticus* a powerful ability to form tetrathionate and activate the TtrRS under different conditions. Oxygen deficiency is a rather common phenomenon in marine waters, while an active sulfur cycle exists in suboxic zones of marine waters [24]. Thus, TtrRS and its target genes may also assist *V. parahaemolyticus* in proliferation within marine environments. In addition, our results demonstrated that TtrRS has a positive autoregulation (Fig 3). A common property of bacterial regulatory circuits upon detecting external signals is autoregulation, which enhances sensitivity to input signals [48]. These findings further suggested that *V. parahaemolyticus* has a tetrathionate-responsive genetic circuit that could swiftly respond to the environmental thiosulfate or tetrathionate. Such a system increases the ability of *V. parahaemolyticus* to respond to rapid changes of sulfur availability during the early steps of infection and optimizes the bacterial colonization in the host gut.

In conclusion, this study unveils a model that describes a novel regulatory and metabolic pathway in enteric pathogens. For optimal growth during infection, HK TtrS sense tetrathionate and phosphorylate the RR TtrR, which further activates the sulfur utilization including reduction and oxidation by constituting a tetrathionate-responsive genetic circuit. Therefore, *V. parahaemolyticus* could adapt to niche-specific nutrients and increase colonization in the host gut. These findings promote a better understanding of the mechanism by which pathogens explore the host sources and correlations between bacterial metabolism and virulence. However, further investigations should determine how the pathogen balances the sulfur reduction and oxidation by TtrRS during the infection, and a comprehensive study should be applied to identify the roles of the potential cross-talk between the two TCSs.

## Materials and methods

### Ethics statement

All animal experiments were approved by the Laboratory Animal Welfare and Ethics Committee of Zhejiang A&F University, China (approval number ZAFUAC2022007). The Chinese National Laboratory Animal Guideline for Ethical Review of Animal Welfare adhered to animal care and protocol.

### Bacterial strains, plasmids, and culture conditions

The bacterial strains and plasmids used in this study are shown in S2 Table. All *V. parahaemolyticus* strains were derived from the HZ strain, a clinical isolate from the Zhejiang Provincial Center for Disease Control and Prevention, Zhejiang, China [49,50]. The *V. parahaemolyticus* strain was streptomycin-resistant and grown at 37°C in modified Luria-Bertani (MLB, 10 g/L tryptone, 5 g/L yeast extract, 30 g/L NaCl, pH 7.4) broth. *E*. *coli* strains DH5α, CC118λpir, BL21 (DE3), and BTH101 were grown in Luria-Bertani (LB, 10 g/L tryptone, 5 g/L yeast extract, 10 g/L NaCl, pH 7.4) broth at 37°C. When required, 50 μg/mL streptomycin, 50 μg/mL kanamycin, 5 μg/mL chloromycetin, 10% sucrose, or 0.02% arabinose were added to the culture medium. For micro-aerobic growth, the cultures were purged with nitrogen gas and incubated statically at 37°C.

Growth of *V. parahaemolyticus* strains was measured by recording the values of optical density at 600 nm (OD_600_) in triplicate. Briefly, the strains grown in the logarithmic phase were diluted to OD_600_ of 0.01 in modified M9 minimal medium (22 mM KH_2_PO_4_, 90 mM Na_2_HPO_4_, 8.5 mM NaCl, 20 mM NH_4_Cl, 1% Casein hydrolysate, 0.2 mM MgCl_2_, 0.01 mM CaCl_2_, pH 7.4), which contains sodium thiosulfate or sodium tetrathionate (Sigma-Aldrich) at indicated final concentrations. Then the medium was incubated statically at 37°C under the micro-aerobic conditions and the value of OD_600_ was determined at 2-h intervals using the microplate reader (Bio-Tek).

### Bioinformatics analysis

Amino acid sequences were obtained from the NCBI database (http://blast.ncbi.nlm.nih.gov/) and the MicrobesOnline website (http://www.microbesonline.org/). The 28 RRs were predicted by the P2CS (http://www.p2cs.org/) prokaryote database. The Basic Local Alignment Search Tool (BLAST, available from the NCBI website) was used to analyze the similarity between amino acid sequences. The MEGA7 software was applied to perform multiple sequence alignments and further construct a phylogenetic tree based on the amino acid sequences of TtrA using the neighbor-joining method. Three-dimensional structures and domains of 09835 and 09830 were predicted by the SWISS-MODEL online server (https://www.swissmodel.expasy.org/) and the SMART database [51–54], respectively. The BProm program (SoftBerry) was used to predict the promoter region of each target gene. The MEME Suite (https://meme-suite.org/meme/) was used to discover the consensus TtrR binding motif in the promoter region [55,56].

### Recombinant DNA techniques

In-frame markerless deletion strains were constructed by homologous recombination using suicide vector pDS132 as described previously [57]. The recombinant pDS132 plasmid harboring the upstream and downstream homology arms of the target gene was introduced into *E*. *coli* DH5α for amplification and further transferred into *V. parahaemolyticus* by *E*. *coli* CC118λpir using a conjugation method. The recombinant plasmid contains a chloromycetin resistance cassette and a sucrose sensitivity gene *sacB*, which could exchange genetic fragments twice with *V. parahaemolyticus* genomes by intermolecular recombination. Putative deletion mutants were screened on the plates with sucrose and streptomycin, and the positive clone was verified by PCR and sequencing. The CΔ*ttrR*^WT^ and CΔ*ttrR*^D58A^ strains were constructed based on the Δ*ttrR* strain by replenishing *ttrR* or *ttrR*^D58A^ to the gene native locus, while the CΔ*ttrS*^WT^ and CΔ*ttrS*^H397A^ strains were constructed based on the Δ*ttrS* strain by replenishing *ttrS* or *ttrS*^H397A^ to the gene native locus. The mutant strains were further verified by sequencing.

The pBBR-*lux* plasmid harboring bioluminescence *luxCDABE* was used to identify the activity of the promoter of the target gene. The predicted promoter region of each target gene was amplified by PCR using specific primers, while the promoter sequence with *ttrR* box deletion was obtained by overlap extension PCR. These PCR products were subcloned into the fluorescent reporter pBBR-*lux*, respectively. The recombinant plasmid was transferred into *E*. *coli* DH5α for amplification, which was verified by sequencing and used for bioluminescence detection.

The pBAD24 plasmid containing an arabinose inducible promoter was used to overexpress the proteins and corresponding mutants. The pKNT25 and pUT18 plasmids were used for the bacterial two-hybrid assay [58]. The pET28a plasmid was used to express the recombinant TtrR protein. The open reading frames of the target genes were cloned using PCR with specific primers and further inserted into these plasmids either individually or together, which were transferred into *E*. *coli* for amplification and verified by sequencing. The restriction and DNA-modifying enzymes were purchased from APExBIO or New England BioLabs and performed according to the supplier’s instructions. The primers used for PCR amplification were designed by Clone Manager 9 and listed in S3 Table.

### RNA extraction, qRT- PCR, and transcriptome sequencing

The *V. parahaemolyticus* strains in the logarithmic phase were harvested by centrifugation and washed three times with phosphate-buffered saline (PBS). Total RNA was extracted using the TRIzol reagent (Vazyme, China) according to the supplier’s instructions. The RNA purity was assessed using the Nanodrop2000 (Thermo Fisher Scientific). The genomic DNA was removed from the RNA and cDNA was synthesized using HiScript II 1st Strand cDNA Synthesis Kit (with gDNA wiper, Vazyme). qRT-PCR analysis was performed using the Mx3000P PCR detection system (Agilent) and ChamQ universal SYBR qPCR master mix (Vazyme), while the housekeeping gene 16S rRNA was used as an internal control [59]. The threshold cycle (2^−ΔΔCT^) method was applied to quantify the relative mRNA levels [60]. The primers used for qRT-PCR analysis are listed in S3 Table, and each sample procedure was repeated three times.

Transcriptomic profiles of *V. parahaemolyticus* were analyzed using RNA sequencing (RNA-seq). Each sample in RNA-seq assay was repeated twice. The sequencing library was constructed by TruSeq Stranded Total RNA Library Prep Kit and sequenced using the Illumina HiSeq 2000 platform. The extracted RNA samples were fragmented to yield fragments in the range of 60–200 nt, which were used for the synthesis of first-strand cDNA by random primers. After the second-strand cDNA was synthesized, the Illumina-specific adaptors were added to the cDNA. After filtering out low-quantity sequences, the clean reads were aligned to the *V. parahaemolyticus* genome. Differential gene expression analysis was performed using DESeq2 (version 1.6.3). All genes with *q* values of < 0.05 and estimated fold changes of ≥ 2 were declared significant. To determine the functions of the differentially expressed genes, KEGG enrichment and GO categories were carried out using Goatools and KOBAS [61].

### Bioluminescence reporter assay

The recombinant plasmid pBBR-*lux* was introduced into *V. parahaemolyticus* or *E*. *coli* strains using a conjugation method. The *E*. *coli* strains used for bioluminescence detection harbor additional pBAD24 plasmid to overexpress the indicated proteins. The *V. parahaemolyticus* or *E*. *coli* strains harboring the bioluminescence plasmid were cultured in fresh MLB, LB, or modified M9 with sodium thiosulfate or sodium tetrathionate at 37°C for 5–7 h. Luminescence intensity and bacterial growth (OD_600_) were measured using a Bio-Tek Synergy HT spectrophotometer. Luminescence expression was reported as luminescence units/OD_600_. Each sample procedure was repeated at least three times.

### Electrophoretic mobility shift assay

Electrophoretic mobility shift assay (EMSA) was performed to analyze the binding of TtrR to the DNA probe [62]. Briefly, the recombinant plasmid pET28a-*ttrR* was transformed into *E*. *coli* BL21(DE3) competent cells, and the expression of TtrR was induced with the addition of 0.1 mM Isopropyl-β-D-thiogalactopyranoside (IPTG, Sigma-Aldrich) at 16°C for 10 h. The bacterial cells were collected via centrifugation and lysed by sonication, and the supernatant was applied to a Ni-nitrilotriacetic acid spin column (GE Healthcare) following the manufacturer’s instructions. After purification, the sample was dialyzed overnight at 4°C. The recombinant protein was further analyzed by SDS-PAGE. The promoter sequences were obtained by PCR amplification and the sequences with *ttrR* box deletion were obtained by overlap extension PCR. The PCR products were purified using a gel extraction kit (Vazyme), which were used as DNA probes. EMSAs were performed by the addition of increasing amounts of TtrR protein (0 to 500 nM) to the DNA probe (30 nM) in binding buffer (10 mM Tris, EDTA 1 mM, 1 mM dithiothreitol, 50 mM KCl, 50 mM MgCl_2_, 10% glycerol), followed by a 30 min incubation at 37°C. The reaction mixtures were subjected to electrophoresis on a 6% polyacrylamide gel in 0.5 × TBE buffer (44.5 mM Tris base, 44.5 mM boric acid, 1 mM EDTA, pH 7.4) on ice at 200 V for 45 min. The gel was stained in 0.5 × TBE buffer containing 1 × SYBR gold nucleic acid stain (Invitrogen) for 20 min and the image was recorded.

### Bacterial two-hybrid assay

The bacterial two-hybrid assay was performed to test the interaction between TtrR proteins according to the manufacturer’s instructions [58]. The TtrR proteins were fused with the T25 and T18 fragments respectively by the recombinant plasmids pKNT25 and pUT18, which were co-transformed into *E*. *coli* BTH101 component cells. The T25 and T18 fragments are not active when physically separated, whereas the homodimerization of TtrR proteins results in functional complementation between the two fragments and cAMP synthesis. The efficiency of complementation between fusion proteins can be further quantified by assaying the β-galactosidase enzymatic activities, which are positively regulated by cAMP. The *E*. *coli* BTH101 strains harboring the plasmids were cultured statically in modified M9 containing 0.5 mM IPTG with sodium tetrathionate or not at 30°C for 8 h. The cultures then were assayed for β-galactosidase activity using o-nitrophenol-β-galactoside (ONPG) as a substrate. The β-galactosidase activity was determined by monitoring color development at 420 nm, which was normalized to the values of OD_600_ and presented as Miller units [63]. Each sample procedure was repeated at least three times.

### High-performance liquid chromatography analysis of sulfur compounds

High-performance liquid chromatography (HPLC) was applied to measure the concentrations of sulfur compounds including thiosulfate and tetrathionate in the cultures of *V. parahaemolyticus* strains. The bacterial cells were grown in a modified M9 minimal medium at 37°C under micro-aerobic conditions, and the medium contained sodium thiosulfate or sodium tetrathionate at indicated final concentrations. The samples were collected at 3-h intervals for 12h, which was centrifugated at 14,000 g for 5 min. Then the supernatant fluid was added with acetonitrile to remove proteins, which was further centrifugated and filtered by 0.22 μm mixed cellulose ester membranes. The concentrations of thiosulfate and tetrathionate in the samples were determined using SHIMADZU LC-16 system (Shimadzu, Japan). Standard chemicals including Na_2_S_2_O_3_ and Na_2_S_4_O_6_ were used to prepare the calibration curves for quantification of thiosulfate and tetrathionate.

### H_2_S detection

A lead acetate detection method was used to monitor the H_2_S generation in *V. parahaemolyticus* strains [23]. The modified M9 minimal medium was inoculated with *V. parahaemolyticus* strains to an OD_600_ value of 0.01. Then the lead acetate test paper (Aladdin, China) was affixed to the inner wall of the cultural tube, above the level of the liquid culture. After the cultures were incubated overnight at 37°C, and the paper strips were removed from cultural tubes and the image was recorded.

### Mouse infection assay

Mouse infection studies were performed as previously reported with the following modifications [20]. Specific pathogen-free (SPF) ICR mice (four weeks old, female) were purchased from Hangzhou Medical College and housed in standard cages under SPF conditions. A 1% (wt/vol) solution of streptomycin and 0.8% (wt/vol) sucrose were added to the drinking water for the remainder of the experiment. After pretreated with streptomycin for 3 days, the mice were fasted for 4 h and prepared for inoculation. Firstly, the mice were intragastrically administered 100 μL 10% (wt/vol) NaHCO_3_ to neutralize the stomach acid; and then 5 min later, the mice were intragastrically administered again with 100 μL of *V. parahaemolyticus* strain at a dose of 2.0 × 10^9^ CFU/mouse. The food was returned 2 h postinfection. Fecal pellets were collected from each mouse at the indicated time points, which were serially diluted with PBS and plated onto MLB plates with 50 μg/mL streptomycin to enumerate CFU.

### Statistical analysis

Data were presented as mean ± standard deviation (SD). Statistical analyses were conducted using the unpaired two-tailed Student’s *t*-test or Mann-Whitney test as indicated with the GraphPad software package. For all tests, the differences were considered statistically significant when *P* < 0.05 (*), *P* < 0.01 (**), *P* < 0.001 (***), and *P* < 0.0001 (****).

## Supporting information

S1 Fig Identification of TCSs that contribute to *V. parahaemolyticus* colonization. (**A**) The colonization of RR gene deletion strains. Colonization (CFU) of *V. parahaemolyticus* was measured from feces in the streptomycin-treated adult mouse model at 48 h postinfection. (**B**) Predicted three-dimensional structure and conserved domains of the 09835 protein. (**C**) Predicted three-dimensional structure and conserved domains of the 09830 protein.
(TIF)

S2 Fig TtrSR works as an important regulator. (**A**) GO analysis of the transcriptomic data. The *x*-axis displays the ratio of the number of differentially expressed genes and the number of all the unigenes in the GO terms, while the *y*-axis represents the top 30 enriched GO terms. (**B**) KEGG analysis of the transcriptomic data. The *x*-axis displays the ratio of the number of differentially expressed genes and the number of all the unigenes in the KEGG pathways, while the *y*-axis represents the top 20 enriched KEGG pathways.
(TIF)

S3 Fig Features of the promoter region of TtrRS target genes. The -10 box and -35 box were predicted by the BProm program (SoftBerry). The inverted repeats (IRs) were the TtrR binding box.
(TIF)

S4 Fig Identification of potential phosphorylation sites and the role of tetrathionate in TtrRS activation. (**A**) Protein sequence alignment of DHp domain of HK. (**B**) Protein sequence alignment of REC domain of RR. Amino acid sequences were obtained from the NCBI database. Green arrows below the alignment indicate β strands, and red bars indicate α helices. Asterisk denote potential phosphorylation sites. (**C**) Tetrathionate increases the transcription of TtrRS target genes. The *E*. *coli* containing promoter-*lux* transcriptional fusion plasmids and pBAD24-*ttrS*-*ttrR* were grown in M9 in the absence or in the presence of tetrathionate at 37°C. Luminescence expression was calculated as the luminescence per unit of OD_600_. The unpaired two-tailed Student’s *t*-test was used for statistical analysis (**, *P* < 0.01; ***, *P* < 0.001).
(TIF)

S5 Fig *V. parahaemolyticus* could utilize thiosulfate. (**A**) Homology analyses of amino acid sequences encoded by *09855–09860* genes using BLAST. The identities of the amino acid sequences of each protein are shown between *V. parahaemolyticus* strain HZ and *S*. *oneidensis* strain MR-1. (**B**) Thiosulfate supports the growth of *V. parahaemolyticus*. The strains were grown in M9 in the absence or in the presence of thiosulfate at 37°C under micro-aerobic conditions. Bacteria cell density was measured and reported as the value of OD_600_.
(TIF)

S6 Fig Biological characteristics of *V. parahaemolyticus* strains with the modified promoters. (**A**) mRNA level of *ttrC* and *tsdA* in WT and the relevant mutants was determined by using qRT-PCR. The results are expressed as means ± SD from three independent experiments. (**B**) H_2_S generation by WT and the relevant mutants. The strains were grown in a modified M9 minimal medium containing sodium tetrathionate. H_2_S generation was monitored by lead acetate strips. (**C**) Deletion of the *ttrR* box decreased the colonization of *V. parahaemolyticus*. Colonization (CFU) of *V. parahaemolyticus* was measured from feces in the streptomycin-treated adult mouse model at 48 h postinfection. Mann-Whitney test was used for statistical analysis (**, *P* < 0.01).
(TIF)

S7 Fig TtrRS and their target genes contribute to the intestinal colonization of *V. parahaemolyticus* strain RIMD 2210633. (**A**) The expression level of *ttrBCA* (*vp2011-2014*), and *tsdBA* (*vp2015-2016*) was assessed by measuring luminescence in P_*ttrB* (*vp2011*)_*-lux* and P_*tsdB* (*vp2015*)_*-lux* transcriptional fusion strains, respectively. The *V. parahaemolyticus* strains harboring the P_*ttrB* (*vp2011*)_*-lux* and P_*tsdB* (*vp2015*)_*-lux* were cultured in fresh MLB under aerobic conditions. Luminescence expression was calculated as the luminescence per unit of OD_600_. (**B**) Deletion of *ttrR* (*vp2009*) or its target genes decreased the colonization of *V. parahaemolyticus* strain RIMD 2210633. Colonization (CFU) of *V. parahaemolyticus* was measured from feces in the streptomycin-treated adult mouse model at 48 h post-infection. The unpaired two-tailed Student’s *t*-test (A) or Mann-Whitney test (B) was used for statistical analysis (*, *P* < 0.05; ***, *P* < 0.001; ****, *P* < 0.0001).
(TIF)

S8 Fig Distribution of the *ttrR* box in the genomes of *V. parahaemolyticus*. FIMO was used to scan the *V. parahaemolyticus* genomes for the *ttrR* box. The circular diagram depicts the location of *ttrR* box on the *V. parahaemolyticus* chromosome 1 and 2. Maps were established using the software Proksee (https://proksee.ca/).
(TIF)

S1 Table Differentially expressed genes in the Δ*ttrR* strain versus the WT strain. (DOCX)

S2 Table Bacterial strains and plasmids used in this study. (DOCX)

S3 Table Primers used in this study. (DOCX)
