# Supplementary figures and images for: The two-component system TtrRS boosts Vibrio parahaemolyticus colonization by exploiting sulfur compounds in host gut
Source: PLoS Pathog. 2024 Jul 22;20(7):e1012410. doi: 10.1371/journal.ppat.1012410 (PMC11293645; doi:10.1371/journal.ppat.1012410)

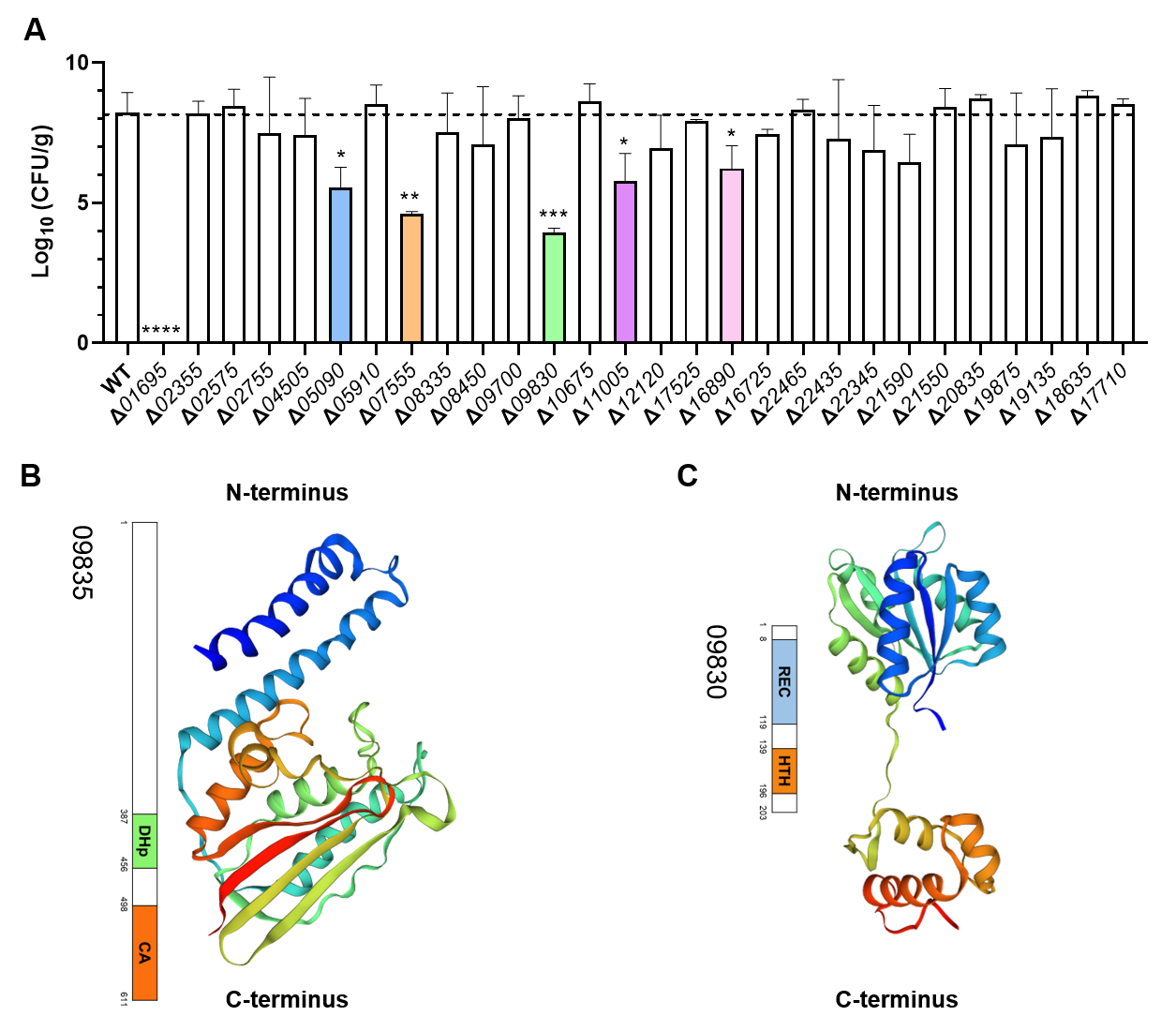

Supplement: S1 Fig — (A) The colonization of RR gene deletion strains. Colonization (CFU) of V. parahaemolyticus was measured from feces in the streptomycin-treated adult mouse model at 48 h postinfection. (B) Predicted three-dimensional structure and conserved domains of the 09835 protein. (C) Predicted three-dimensional structure and conserved domains of the 09830 protein. (TIF) [file ppat.1012410.s001.tif]

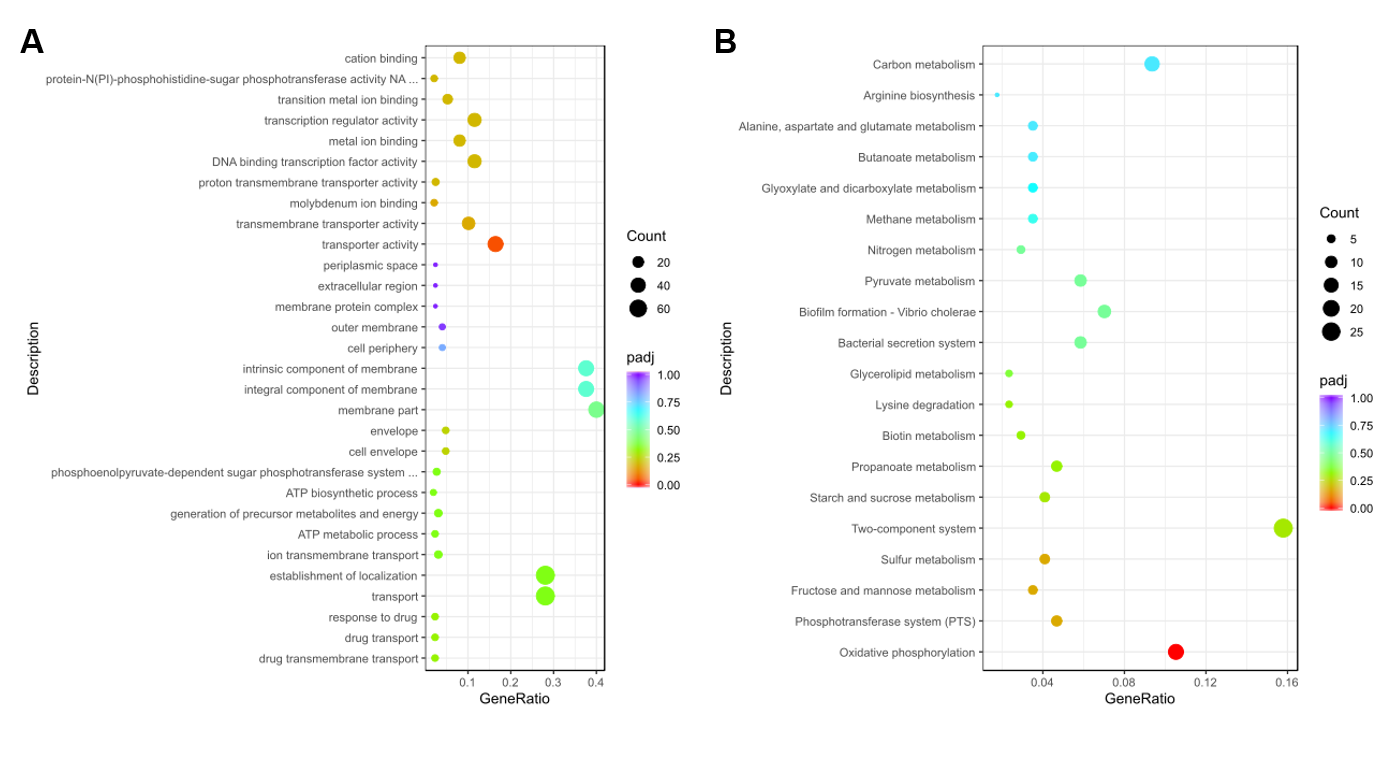

Supplement: S2 Fig — (A) GO analysis of the transcriptomic data. The x-axis displays the ratio of the number of differentially expressed genes and the number of all the unigenes in the GO terms, while the y-axis represents the top 30 enriched GO terms. (B) KEGG analysis of the transcriptomic data. The x-axis displays the ratio of the number of differentially expressed genes and the number of all the unigenes in the KEGG pathways, while the y-axis represents the top 20 enriched KEGG pathways. (TIF) [file ppat.1012410.s002.tif]

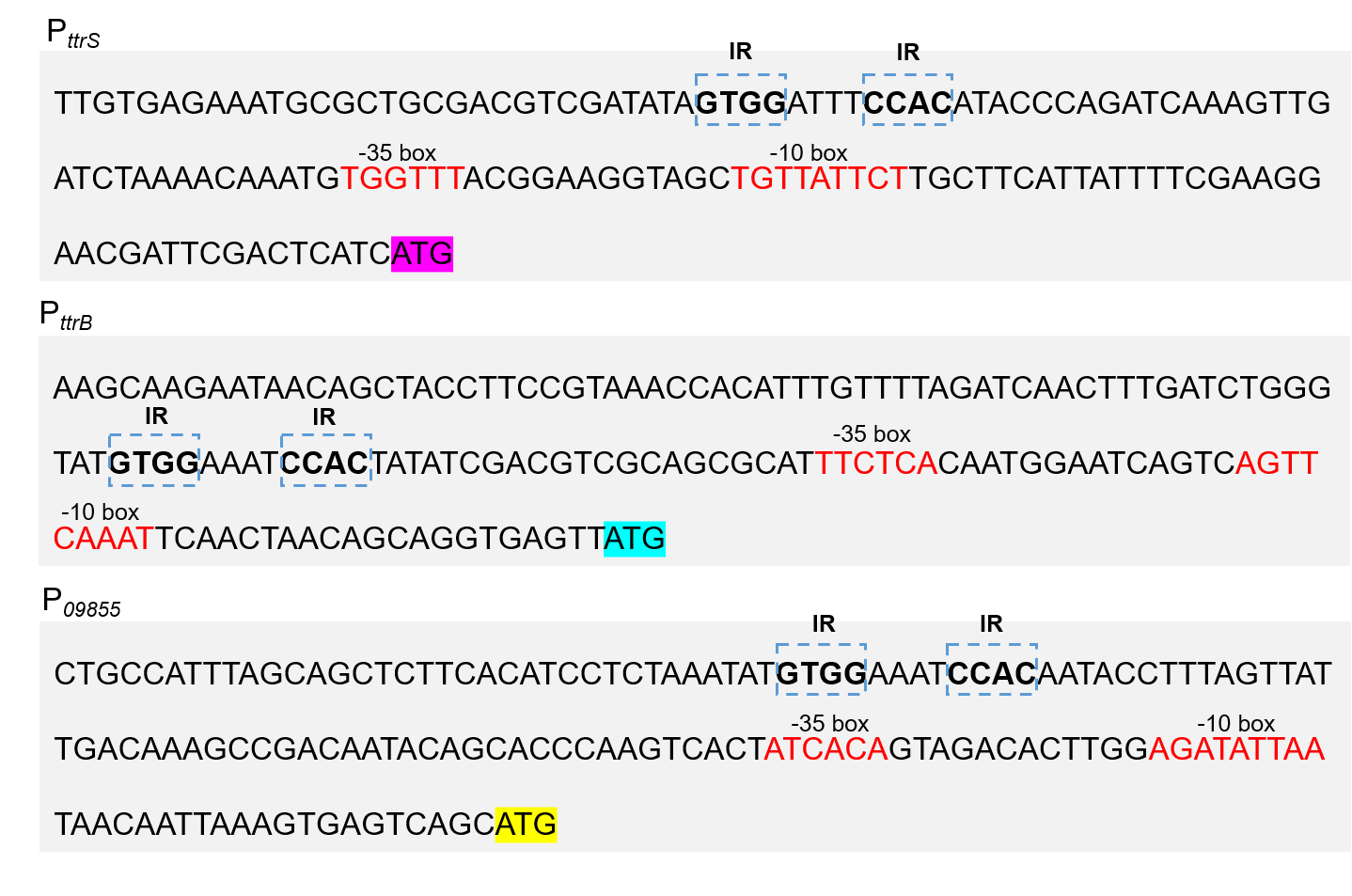

Supplement: S3 Fig — The -10 box and -35 box were predicted by the BProm program (SoftBerry). The inverted repeats (IRs) were the TtrR binding box. (TIF) [file ppat.1012410.s003.tif]

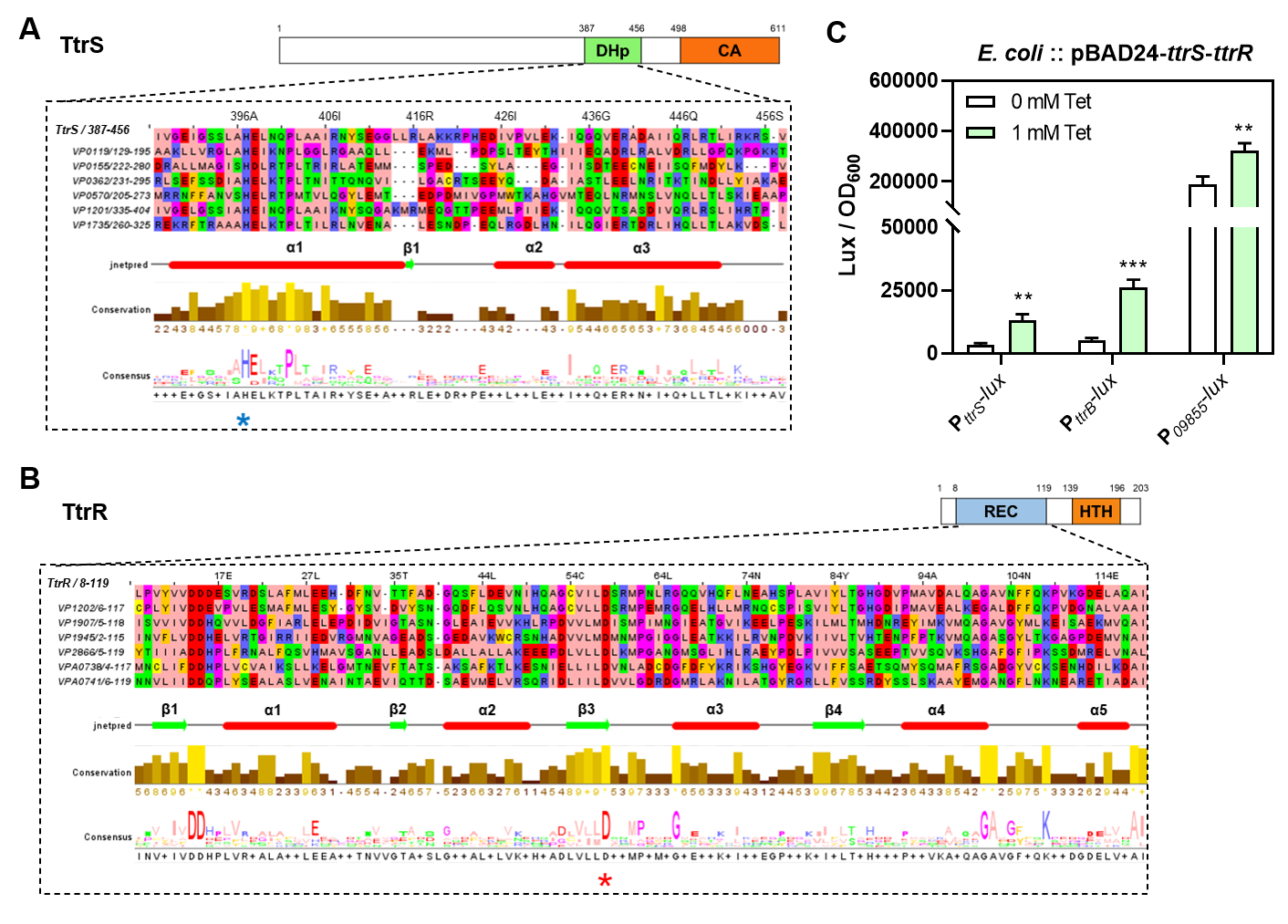

Supplement: S4 Fig — (A) Protein sequence alignment of DHp domain of HK. (B) Protein sequence alignment of REC domain of RR. Amino acid sequences were obtained from the NCBI database. Green arrows below the alignment indicate β strands, and red bars indicate α helices. Asterisk denote potential phosphorylation sites. (C) Tetrathionate increases the transcription of TtrRS target genes. The E. coli containing promoter-lux transcriptional fusion plasmids and pBAD24-ttrS-ttrR were grown in M9 in the absence or in the presence of tetrathionate at 37°C. Luminescence expression was calculated as the luminescence per unit of OD600. The unpaired two-tailed Student’s t-test was used for statistical analysis (**, P < 0.01; ***, P < 0.001). (TIF) [file ppat.1012410.s004.tif]

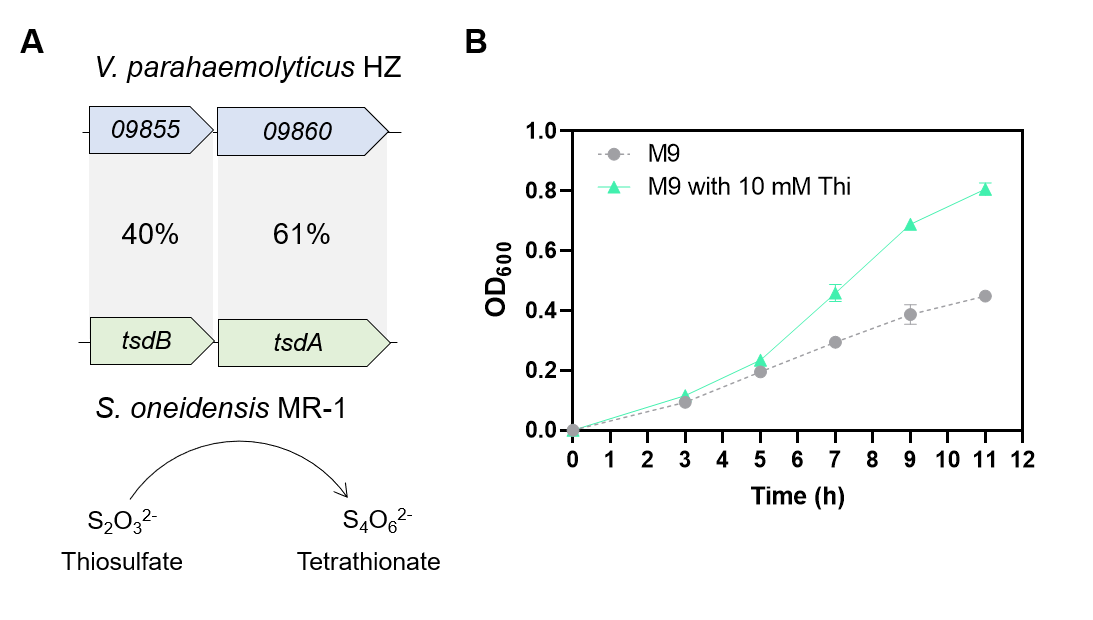

Supplement: S5 Fig — (A) Homology analyses of amino acid sequences encoded by 09855–09860 genes using BLAST. The identities of the amino acid sequences of each protein are shown between V. parahaemolyticus strain HZ and S. oneidensis strain MR-1. (B) Thiosulfate supports the growth of V. parahaemolyticus. The strains were grown in M9 in the absence or in the presence of thiosulfate at 37°C under micro-aerobic conditions. Bacteria cell density was measured and reported as the value of OD600. (TIF) [file ppat.1012410.s005.tif]

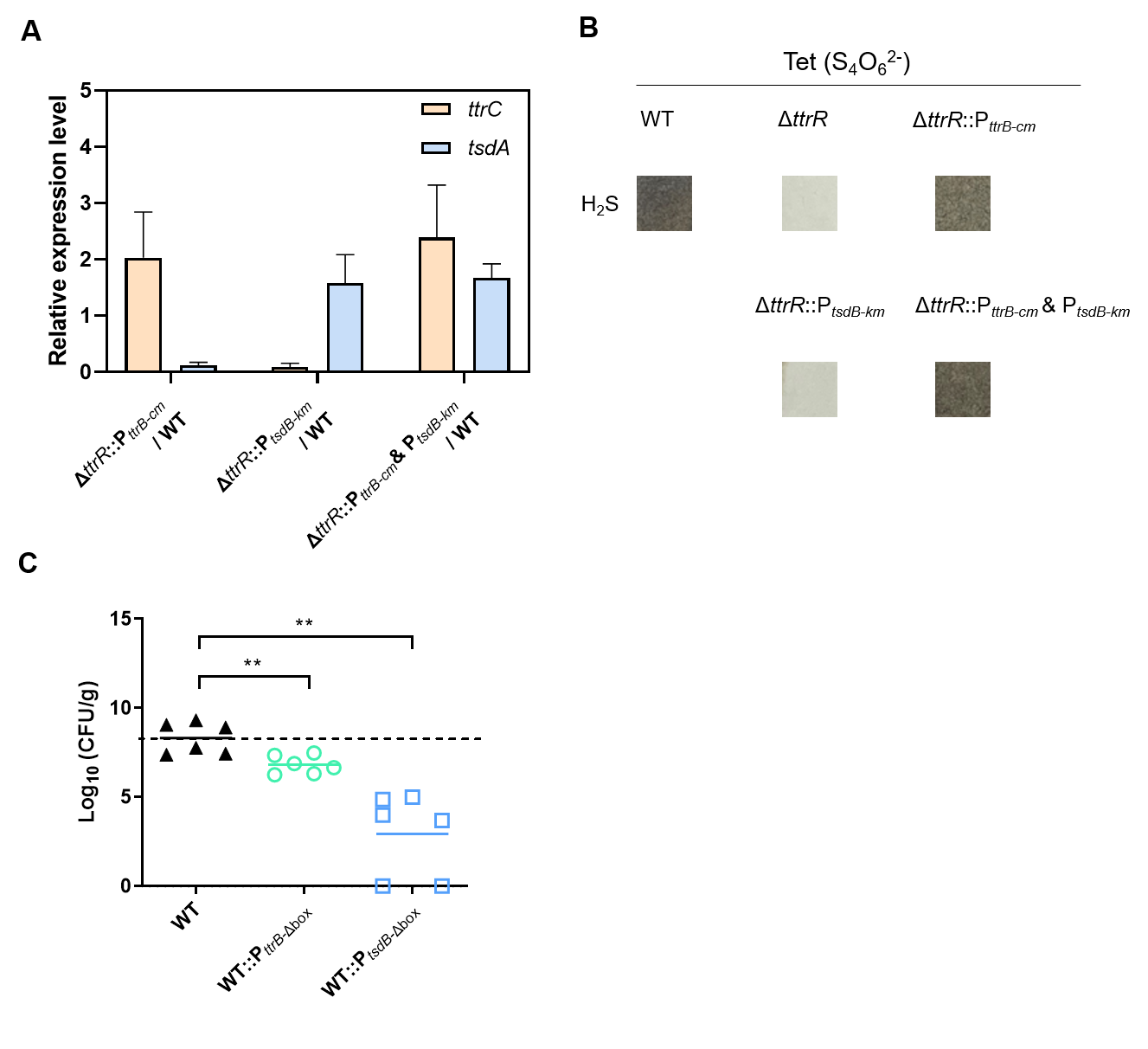

Supplement: S6 Fig — (A) mRNA level of ttrC and tsdA in WT and the relevant mutants was determined by using qRT-PCR. The results are expressed as means ± SD from three independent experiments. (B) H2S generation by WT and the relevant mutants. The strains were grown in a modified M9 minimal medium containing sodium tetrathionate. H2S generation was monitored by lead acetate strips. (C) Deletion of the ttrR box decreased the colonization of V. parahaemolyticus. Colonization (CFU) of V. parahaemolyticus was measured from feces in the streptomycin-treated adult mouse model at 48 h postinfection. Mann-Whitney test was used for statistical analysis (**, P < 0.01). (TIF) [file ppat.1012410.s006.tif]

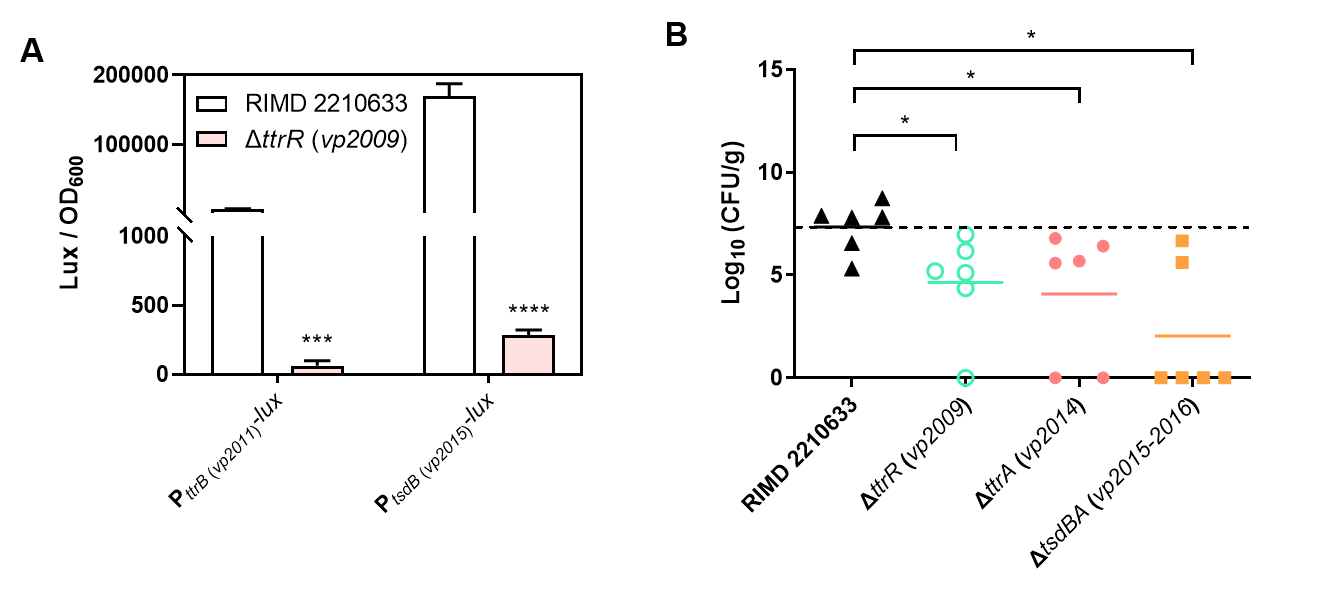

Supplement: S7 Fig — (A) The expression level of ttrBCA (vp2011-2014), and tsdBA (vp2015-2016) was assessed by measuring luminescence in PttrB (vp2011)-lux and PtsdB (vp2015)-lux transcriptional fusion strains, respectively. The V. parahaemolyticus strains harboring the PttrB (vp2011)-lux and PtsdB (vp2015)-lux were cultured in fresh MLB under aerobic conditions. Luminescence expression was calculated as the luminescence per unit of OD600. (B) Deletion of ttrR (vp2009) or its target genes decreased the colonization of V. parahaemolyticus strain RIMD 2210633. Colonization (CFU) of V. parahaemolyticus was measured from feces in the streptomycin-treated adult mouse model at 48 h post-infection. The unpaired two-tailed Student’s t-test (A) or Mann-Whitney test (B) was used for statistical analysis (*, P < 0.05; ***, P < 0.001; ****, P < 0.0001). (TIF) [file ppat.1012410.s007.tif]

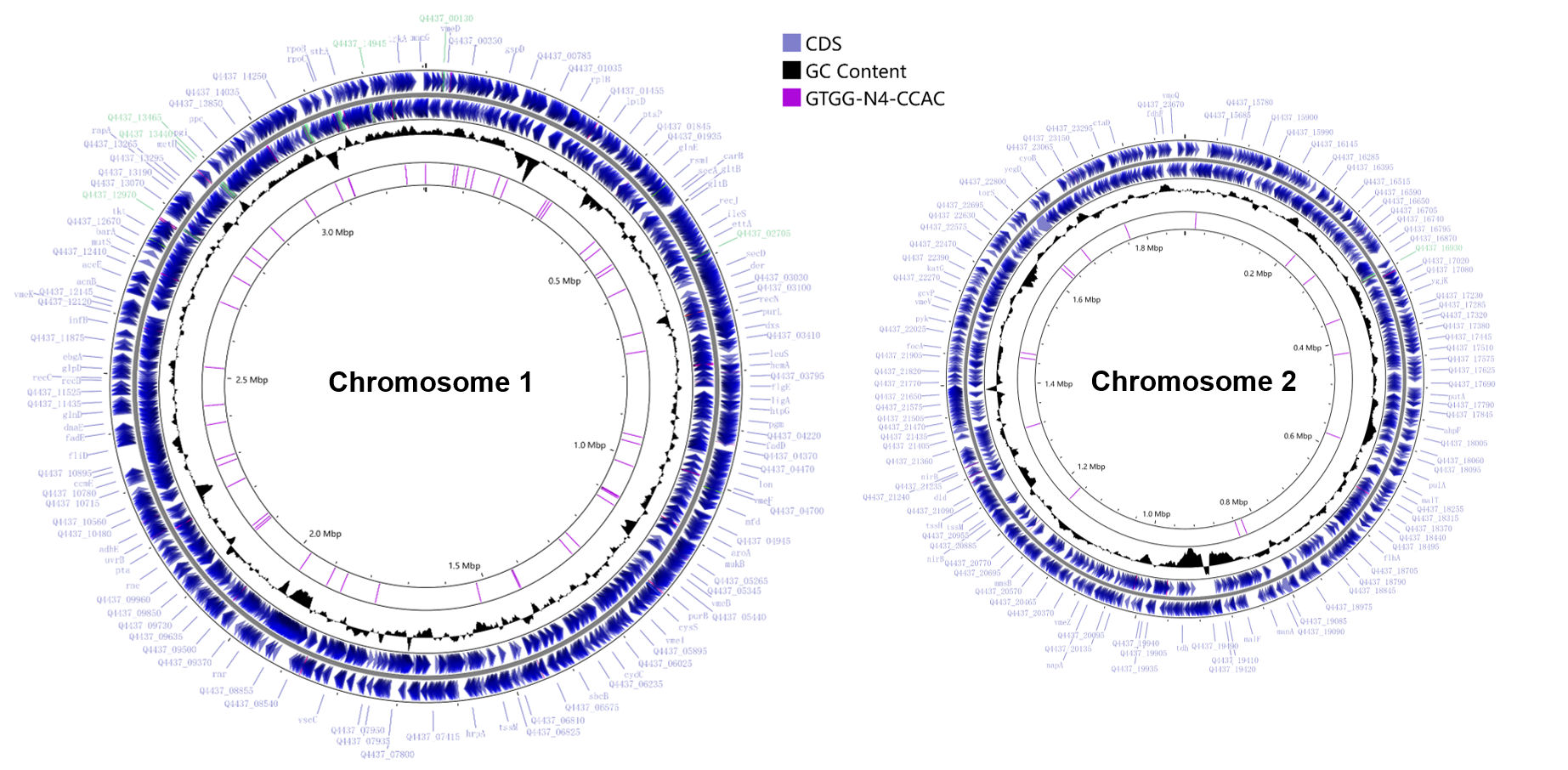

Supplement: S8 Fig — FIMO was used to scan the V. parahaemolyticus genomes for the ttrR box. The circular diagram depicts the location of ttrR box on the V. parahaemolyticus chromosome 1 and 2. Maps were established using the software Proksee (https://proksee.ca/). (TIF) [file ppat.1012410.s008.tif]
