# Supplementary material for: The two-component system TtrRS boosts Vibrio parahaemolyticus colonization by exploiting sulfur compounds in host gut
Source: PLoS Pathog. 2024 Jul 22;20(7):e1012410. doi: 10.1371/journal.ppat.1012410 (PMC11293645; doi:10.1371/journal.ppat.1012410)
Supplement: S1 Table — (DOCX) [file ppat.1012410.s009.docx]

**Table S1 Differentially expressed genes in the Δ*ttrR* strain versus WT strain**

| **Locus_tag** | **Function or description** | **Log2 Fold Change** | ***p*-value** | ***q*-value** |
| --- | --- | --- | --- | --- |
| Q4437_00030 | Hsp20 family protein | -4.616731404 | 3.11E-21 | 2.74E-18 |
| Q4437_00055 | TMEM165/GDT1 family protein | -2.300081944 | 1.84E-06 | 6.94E-05 |
| Q4437_00165 | TetR/AcrR family transcriptional regulator | -1.770353455 | 0.000178092 | 0.002902148 |
| Q4437_00255 | serine/threonine-protein kinase | -2.072116286 | 0.000116842 | 0.002063427 |
| Q4437_00270 | multidrug transporter subunit MdtJ | -2.257494753 | 3.71E-05 | 0.000819491 |
| Q4437_00300 | xanthosine phosphorylase | -1.817986533 | 0.000881508 | 0.010440557 |
| Q4437_00335 | class I SAM-dependent methyltransferase | -1.380778048 | 0.007107623 | 0.049730831 |
| Q4437_00375 | BON domain-containing protein | 2.30668599 | 2.48E-06 | 9.06E-05 |
| Q4437_00385 | hypothetical protein | 4.747680473 | 7.53E-06 | 0.000232577 |
| Q4437_00405 | PA2169 family four-helix-bundle protein | 2.8497153 | 2.73E-06 | 9.71E-05 |
| Q4437_00440 | DNA recombination protein RmuC | -1.414791678 | 0.003716302 | 0.031192915 |
| Q4437_00600 | ribosome-associated heat shock protein Hsp15 | -1.832431049 | 0.000951973 | 0.011089606 |
| Q4437_00755 | hypothetical protein | 1.402967749 | 0.0019005 | 0.019026545 |
| Q4437_00760 | energy transducer TonB | 1.825613773 | 0.000256125 | 0.003885883 |
| Q4437_00770 | MotA/TolQ/ExbB proton channel family protein | 1.849288066 | 0.000537939 | 0.007089557 |
| Q4437_00775 | MotA/TolQ/ExbB proton channel family protein | 1.267059588 | 0.005295639 | 0.039294532 |
| Q4437_00780 | DUF3450 domain-containing protein | 1.415431996 | 0.003274718 | 0.028398484 |
| Q4437_00785 | TonB-dependent receptor | 1.241138041 | 0.003648301 | 0.030680473 |
| Q4437_00940 | acetyltransferase | -1.620530606 | 0.001437145 | 0.01532608 |
| Q4437_01150 | transcriptional regulator | -2.045318285 | 2.47E-05 | 0.000593743 |
| Q4437_01180 | ATP-dependent protease subunit HslV | -2.114661029 | 0.00013211 | 0.002296324 |
| Q4437_01200 | 50S ribosomal protein L31 | 1.171479719 | 0.005075928 | 0.038306552 |
| Q4437_01470 | inorganic diphosphatase | 1.26048515 | 0.003454311 | 0.029559742 |
| Q4437_01675 | pyruvate kinase PykF | -1.14597497 | 0.006448834 | 0.046145224 |
| Q4437_01680 | DeoR family transcriptional regulator | -3.982848344 | 2.24E-13 | 5.81E-11 |
| Q4437_01705 | glycerol dehydrogenase | -2.25154725 | 5.61E-07 | 2.53E-05 |
| Q4437_01710 | dihydroxyacetone kinase subunit DhaK | -1.651492498 | 0.000175338 | 0.002894901 |
| Q4437_01715 | dihydroxyacetone kinase subunit DhaL | -1.355959218 | 0.005548996 | 0.040905872 |
| Q4437_01720 | phosphoenolpyruvate--protein phosphotransferase | -1.85489266 | 2.60E-05 | 0.000617228 |
| Q4437_01720 | phosphoenolpyruvate--protein phosphotransferase | -1.973868747 | 0.000282204 | 0.004195049 |
| Q4437_01730 | MltR family transcriptional regulator | -1.717005184 | 0.002863026 | 0.025849203 |
| Q4437_01735 | mannitol-1-phosphate 5-dehydrogenase | -1.254077988 | 0.00609415 | 0.043963516 |
| Q4437_01740 | PTS mannitol transporter subunit IICBA | -1.251837631 | 0.004306111 | 0.034131921 |
| Q4437_01745 | GNAT family N-acyltransferase | -3.21359839 | 0.000154712 | 0.00262713 |
| Q4437_01755 | SIMPL domain-containing protein | 1.249180107 | 0.005527816 | 0.040905872 |
| Q4437_01760 | DUF4382 domain-containing protein | 1.648962881 | 0.000238874 | 0.003702381 |
| Q4437_01780 | DUF4250 domain-containing protein | 1.842907603 | 2.31E-05 | 0.000567336 |
| Q4437_01840 | site-specific DNA-methyltransferase | 1.210667959 | 0.004107163 | 0.033248759 |
| Q4437_01850 | MarR family transcriptional regulator | -1.525161418 | 0.002414359 | 0.022775908 |
| Q4437_01955 | GatB/YqeY domain-containing protein | -1.158070281 | 0.006084426 | 0.043963516 |
| Q4437_02120 | cytochrome bc complex cytochrome b subunit | 1.172100687 | 0.005024679 | 0.038182372 |
| Q4437_02290 | TRIC cation channel family protein | -1.78783392 | 0.003196233 | 0.028050368 |
| Q4437_02325 | glutamate synthase subunit beta | -2.080615828 | 0.000138125 | 0.002382111 |
| Q4437_02330 | glutamate synthase large subunit | -2.394812791 | 1.07E-05 | 0.000308725 |
| Q4437_02350 | hypothetical protein | 1.409325943 | 0.00336081 | 0.029013845 |
| Q4437_02405 | hemerythrin domain-containing protein | 1.438592726 | 0.000641696 | 0.008188118 |
| Q4437_02410 | DUF3545 family protein | 1.252000619 | 0.003364686 | 0.029013845 |
| Q4437_02475 | DUF1127 domain-containing protein | -1.617647784 | 0.000603576 | 0.00775368 |
| Q4437_02490 | DNA mismatch repair endonuclease MutH | -1.584561987 | 0.003962214 | 0.032758755 |
| Q4437_02865 | inositol-1-monophosphatase | -1.506783105 | 0.000712538 | 0.008911776 |
| Q4437_02970 | exodeoxyribonuclease VII large subunit | -1.266162741 | 0.003548021 | 0.030124065 |
| Q4437_02995 | acetate uptake transporter | -9.103842688 | 4.84E-12 | 9.29E-10 |
| Q4437_03015 | LysR substrate-binding domain-containing protein | -1.810424008 | 0.000306115 | 0.004490027 |
| Q4437_03020 | hypothetical protein | 2.05395177 | 1.75E-05 | 0.00045047 |
| Q4437_03025 | lipase family protein | 1.54831988 | 0.000571048 | 0.007437105 |
| Q4437_03030 | amidohydrolase | 1.55601169 | 0.000301109 | 0.004431317 |
| Q4437_03185 | molecular chaperone DnaK | -1.549424408 | 0.000236005 | 0.003681849 |
| Q4437_03190 | molecular chaperone DnaJ | -2.355982069 | 5.10E-07 | 2.37E-05 |
| Q4437_03220 | AbgT family transporter | 1.847620219 | 2.44E-05 | 0.000589299 |
| Q4437_03250 | DNA-directed RNA polymerase subunit beta | -3.619200608 | 8.17E-05 | 0.001555565 |
| Q4437_03375 | alpha/beta fold hydrolase | -1.498329071 | 0.006716517 | 0.047521511 |
| Q4437_03450 | trehalose operon repressor TreR | -1.70663627 | 0.002621569 | 0.024012918 |
| Q4437_03455 | PTS trehalose transporter subunit IIBC | -2.825236715 | 7.43E-09 | 6.83E-07 |
| Q4437_03460 | alpha,alpha-phosphotrehalase | -3.286971387 | 6.00E-10 | 6.62E-08 |
| Q4437_03465 | hypothetical protein | 2.342741644 | 7.00E-08 | 5.33E-06 |
| Q4437_03780 | beta-N-acetylhexosaminidase | -1.917379359 | 0.000436041 | 0.006072941 |
| Q4437_03815 | OmpA family protein | 1.186924819 | 0.00443304 | 0.034847451 |
| Q4437_04045 | sulfite exporter TauE/SafE family protein | -3.658067203 | 5.99E-07 | 2.67E-05 |
| Q4437_04060 | molecular chaperone HtpG | -1.586663989 | 0.000359024 | 0.005197012 |
| Q4437_04075 | peptide MFS transporter | -2.208156851 | 3.57E-05 | 0.000795329 |
| Q4437_04170 | succinate dehydrogenase, hydrophobic membrane anchor protein | 1.193767528 | 0.00554686 | 0.040905872 |
| Q4437_04250 | VOC family protein | -3.770525707 | 2.88E-05 | 0.000673898 |
| Q4437_04325 | NupC/NupG family nucleoside CNT transporter | -7.564539079 | 5.45E-06 | 0.000179409 |
| Q4437_04405 | hypothetical protein | -2.096405615 | 0.002205393 | 0.021305931 |
| Q4437_04485 | TRAP transporter substrate-binding protein | 3.270457008 | 3.74E-12 | 8.26E-10 |
| Q4437_04495 | TRAP transporter large permease | 2.85745189 | 3.33E-08 | 2.77E-06 |
| Q4437_04675 | type II secretion system protein | -4.241896874 | 1.56E-09 | 1.61E-07 |
| Q4437_04690 | PLP-dependent cysteine synthase family protein | -3.037623306 | 0.002364823 | 0.022554288 |
| Q4437_04700 | cadherin repeat domain-containing protein | -2.367159977 | 9.63E-07 | 4.21E-05 |
| Q4437_04740 | ferredoxin family protein | -2.362102203 | 0.000147018 | 0.00252562 |
| Q4437_04755 | zinc/cadmium/mercury/lead-transporting ATPase | -1.890805081 | 1.06E-05 | 0.000308725 |
| Q4437_04775 | purine nucleoside phosphoramidase | -1.240782217 | 0.005076189 | 0.038306552 |
| Q4437_04875 | SpoVR family protein | 2.061142853 | 2.75E-06 | 9.71E-05 |
| Q4437_04880 | YeaH/YhbH family protein | 1.378043197 | 0.001529807 | 0.016004974 |
| Q4437_04885 | PrkA family serine protein kinase | 1.719213693 | 5.32E-05 | 0.001101082 |
| Q4437_04900 | pyruvate formate lyase 1-activating protein | -1.24108111 | 0.004081945 | 0.033189299 |
| Q4437_04915 | DUF3360 family protein | -1.396740785 | 0.002815572 | 0.025490057 |
| Q4437_04920 | flagellar sheath protein A | 1.408280862 | 0.0012415 | 0.013771912 |
| Q4437_04965 | TSUP family transporter | -1.702737202 | 0.001792649 | 0.018227019 |
| Q4437_04995 | cold shock domain-containing protein CspD | 1.312650343 | 0.002500992 | 0.023246065 |
| Q4437_05010 | serine hydrolase | -1.927116691 | 0.005911861 | 0.043070738 |
| Q4437_05050 | glucose-1-phosphate adenylyltransferase | 1.409591257 | 0.00101471 | 0.011758385 |
| Q4437_05280 | site-specific integrase | -2.014242821 | 0.00360343 | 0.030419014 |
| Q4437_05290 | helix-turn-helix transcriptional regulator | -2.479877758 | 0.000162828 | 0.002733398 |
| Q4437_05395 | tryptophan--tRNA ligase | 1.569878588 | 0.001747336 | 0.017857613 |
| Q4437_05405 | sphingomyelin phosphodiesterase | -3.695638337 | 0.002133716 | 0.020795492 |
| Q4437_05410 | tRNA isopentenyl-2-thiomethyl-A-37 hydroxylaseMiaE | -2.254531994 | 0.000115634 | 0.002061067 |
| Q4437_05425 | methyltransferase | -1.90218886 | 0.00195502 | 0.019396432 |
| Q4437_05475 | adenosylmethionine--8-amino-7-oxononanoatetransaminase | -2.814892419 | 2.30E-05 | 0.000567336 |
| Q4437_05480 | biotin synthase BioB | -2.513951534 | 1.08E-06 | 4.64E-05 |
| Q4437_05485 | 8-amino-7-oxononanoate synthase | -2.906415858 | 2.25E-07 | 1.19E-05 |
| Q4437_05490 | malonyl-ACP O-methyltransferase BioC | -2.878540937 | 6.24E-06 | 0.000201215 |
| Q4437_05495 | dethiobiotin synthase | -2.207610992 | 1.13E-05 | 0.000324708 |
| Q4437_05515 | SDR family oxidoreductase | 1.404326181 | 0.004746137 | 0.036633208 |
| Q4437_05520 | NAD(P)/FAD-dependent oxidoreductase | 1.522050894 | 0.001145362 | 0.012966087 |
| Q4437_05525 | DUF1365 family protein | 2.048960241 | 0.000329003 | 0.00479389 |
| Q4437_05530 | cyclopropane-fatty-acyl-phospholipid synthase family protein | 1.570481718 | 0.000830891 | 0.010022902 |
| Q4437_05585 | hypothetical protein | -2.990212926 | 2.27E-06 | 8.34E-05 |
| Q4437_05595 | ATP phosphoribosyltransferase | -1.181145939 | 0.006331801 | 0.045455125 |
| Q4437_05650 | peptidylprolyl isomerase | 1.23462615 | 0.0041271 | 0.033250264 |
| Q4437_05690 | formate transporter FocA | -3.136114842 | 1.36E-06 | 5.61E-05 |
| Q4437_05805 | lipase secretion chaperone | 2.193301656 | 0.000238999 | 0.003702381 |
| Q4437_05820 | cardiolipin synthase | -1.46005599 | 0.001467964 | 0.015579473 |
| Q4437_05830 | methyl-accepting chemotaxis protein | -2.743848267 | 2.36E-07 | 1.21E-05 |
| Q4437_05890 | (Fe-S)-binding protein | -2.876562385 | 9.22E-05 | 0.001724812 |
| Q4437_05895 | FAD-dependent oxidoreductase | -4.177588024 | 0.000164371 | 0.002748848 |
| Q4437_05905 | PhnD/SsuA/transferrin family substrate-binding protein | -1.630187526 | 0.004272904 | 0.033929625 |
| Q4437_05910 | response regulator | -2.160876373 | 0.002409866 | 0.022775908 |
| Q4437_06020 | ABC transporter ATP-binding protein | -2.559671485 | 2.19E-06 | 8.21E-05 |
| Q4437_06040 | TetR/AcrR family transcriptional regulator | -1.651617334 | 0.000472259 | 0.006455186 |
| Q4437_06045 | acyl-CoA dehydrogenase | -1.649724611 | 0.000112723 | 0.002023049 |
| Q4437_06080 | hypothetical protein | 1.750588839 | 4.82E-05 | 0.001033213 |
| Q4437_06200 | phosphoribosylaminoimidazolesuccinocarboxamide synthase | 1.231763294 | 0.00419958 | 0.033589029 |
| Q4437_06205 | SulA-like leucine-rich domain-containing protein | -1.76087119 | 0.000794463 | 0.009609734 |
| Q4437_06215 | J domain-containing protein | -2.192001302 | 6.56E-05 | 0.001263861 |
| Q4437_06265 | imidazolonepropionase | -1.593860349 | 0.000279683 | 0.004178902 |
| Q4437_06270 | histidine utilization repressor | -3.332020919 | 1.29E-10 | 1.78E-08 |
| Q4437_06300 | 50S ribosomal protein L20 | 1.170272426 | 0.004985889 | 0.038018479 |
| Q4437_06315 | TetR/AcrR family transcriptional regulator | -1.849461773 | 2.71E-05 | 0.000636306 |
| Q4437_06320 | MMPL family transporter | -2.143924977 | 0.000506343 | 0.006838872 |
| Q4437_06370 | formate-dependent phosphoribosylglycinamide formyltransferase | 1.290827997 | 0.004106021 | 0.033248759 |
| Q4437_06375 | cytidine deaminase | 1.38228794 | 0.001835247 | 0.018456983 |
| Q4437_06450 | DUF1415 domain-containing protein | -1.928901076 | 0.004060649 | 0.033119591 |
| Q4437_06505 | hypothetical protein | 2.233332178 | 1.53E-05 | 0.000404265 |
| Q4437_06510 | S41 family peptidase | 1.708357795 | 0.000111541 | 0.002018243 |
| Q4437_06535 | FAD-dependent oxidoreductase | -2.558063074 | 1.92E-05 | 0.00048445 |
| Q4437_06540 | polyamine ABC transporter substrate-binding protein | -2.448935681 | 0.000204856 | 0.003276958 |
| Q4437_06570 | hypothetical protein | -2.067418193 | 0.001208671 | 0.013543869 |
| Q4437_06575 | collagenase | 2.060357106 | 1.24E-05 | 0.000343945 |
| Q4437_06800 | TerC family protein | -3.214938546 | 1.83E-06 | 6.94E-05 |
| Q4437_06805 | peptidoglycan DD-metalloendopeptidase family protein | -2.868965709 | 0.005247305 | 0.039265852 |
| Q4437_06830 | sigma-54-dependent Fis family transcriptional regulator | -3.508711461 | 0.000167283 | 0.002787001 |
| Q4437_06965 | hypothetical protein | -1.725271565 | 0.004688294 | 0.036250119 |
| Q4437_06970 | hypothetical protein | -2.086356267 | 0.000759269 | 0.009337534 |
| Q4437_06980 | 2OG-Fe(II) oxygenase | -5.029299253 | 4.78E-07 | 2.26E-05 |
| Q4437_07030 | sodium/glutamate symporter | -7.439700864 | 1.27E-05 | 0.000347889 |
| Q4437_07090 | DmsA/YnfE/YnfF family dimethyl sulfoxide reductase | -2.993130553 | 1.93E-10 | 2.51E-08 |
| Q4437_07095 | dimethylsulfoxide reductase subunit B | -2.437438656 | 0.000404469 | 0.00577907 |
| Q4437_07100 | dimethyl sulfoxide reductase anchor subunit | -2.123392693 | 0.00010106 | 0.001859083 |
| Q4437_07105 | molecular chaperone TorD family protein | -2.179056866 | 0.005286278 | 0.039294532 |
| Q4437_07130 | outer membrane beta-barrel protein | 1.282473158 | 0.003594214 | 0.030419014 |
| Q4437_07195 | sigma-54 dependent transcriptional regulator | 1.362301428 | 0.002954601 | 0.026352656 |
| Q4437_07200 | Hpt domain-containing protein | 2.731326586 | 0.000137515 | 0.0023809 |
| Q4437_07235 | MATE family efflux transporter | -1.668553457 | 0.000940937 | 0.01099004 |
| Q4437_07275 | methyl-accepting chemotaxis protein | -2.283411785 | 0.000660415 | 0.008347409 |
| Q4437_07370 | DUF3305 domain-containing protein | 1.473738543 | 0.003980571 | 0.032787723 |
| Q4437_07395 | formate dehydrogenase subunit alpha | -1.194810207 | 0.004376064 | 0.034500574 |
| Q4437_07400 | formate dehydrogenase FDH3 subunit beta | -1.621168078 | 0.000260052 | 0.003931954 |
| Q4437_07415 | RHS repeat-associated core domain-containing protein | 1.779981971 | 3.81E-05 | 0.000837232 |
| Q4437_07435 | ammonium transporter | -6.792364945 | 0.000517265 | 0.006899466 |
| Q4437_07435 | ammonium transporter | -3.76092639 | 2.27E-07 | 1.19E-05 |
| Q4437_07500 | FNR family transcription factor | -1.21024627 | 0.004267731 | 0.033929625 |
| Q4437_07615 | regulator | -6.312135043 | 0.004174171 | 0.033446394 |
| Q4437_07625 | hypothetical protein | 1.148224073 | 0.006028471 | 0.043757252 |
| Q4437_07705 | hypothetical protein | 1.242264425 | 0.003895224 | 0.032505923 |
| Q4437_07945 | OmpA family protein | 1.405426706 | 0.003909546 | 0.032505923 |
| Q4437_07985 | Cof-type HAD-IIB family hydrolase | -1.853121345 | 0.000773721 | 0.009488834 |
| Q4437_08070 | type III secretion system translocon subunit VopB | 1.354617322 | 0.006369487 | 0.045651435 |
| Q4437_08165 | LysR family transcriptional regulator | -1.987657397 | 0.00031691 | 0.004632971 |
| Q4437_08170 | alpha/beta hydrolase | -1.864587528 | 0.0032271 | 0.028153581 |
| Q4437_08215 | SctL family type III secretion system stator protein VscL | -6.44798703 | 0.002434933 | 0.022775908 |
| Q4437_08295 | aldehyde dehydrogenase family protein | 3.158361531 | 6.31E-12 | 1.11E-09 |
| Q4437_08505 | LpxL/LpxP family Kdo(2)-lipid IV(A) lauroyl/palmitoleoyl acyltransferase | -2.539839487 | 0.000103342 | 0.001885352 |
| Q4437_08540 | response regulator | 1.381921347 | 0.001751714 | 0.017861014 |
| Q4437_08580 | MarR family transcriptional regulator | -1.637417334 | 0.000547968 | 0.007200238 |
| Q4437_08585 | DMT family transporter | -4.757654376 | 3.74E-13 | 9.17E-11 |
| Q4437_08610 | manganese-dependent inorganic pyrophosphatase | -1.981843684 | 0.000558249 | 0.007291918 |
| Q4437_08615 | 4-aminobutyrate--2-oxoglutarate transaminase | -2.347803681 | 1.17E-05 | 0.000330869 |
| Q4437_08620 | NAD-dependent succinate-semialdehyde dehydrogenase | -3.36643738 | 6.54E-08 | 5.07E-06 |
| Q4437_08625 | agmatine deiminase | -4.746866656 | 4.77E-12 | 9.29E-10 |
| Q4437_08635 | IS3-like element ISVpa2 family transposase | -3.376305964 | 5.26E-10 | 5.95E-08 |
| Q4437_08650 | GFA family protein | -2.401814661 | 3.43E-05 | 0.00076836 |
| Q4437_08655 | hypothetical protein | -1.818126281 | 0.002641227 | 0.024142893 |
| Q4437_08660 | hypothetical protein | -1.931503309 | 0.002125384 | 0.020795492 |
| Q4437_08670 | N-acetyltransferase | -2.489569827 | 5.36E-05 | 0.001101082 |
| Q4437_08680 | hypothetical protein | -2.319502456 | 6.83E-05 | 0.00131115 |
| Q4437_08685 | RidA family protein | -1.856571886 | 0.004497715 | 0.03514586 |
| Q4437_08710 | hypothetical protein | -1.766470255 | 0.001512582 | 0.015900115 |
| Q4437_08735 | nuclear transport factor 2 family protein | -1.96584345 | 0.002322339 | 0.022337971 |
| Q4437_08740 | hypothetical protein | -1.662430316 | 0.002358791 | 0.022554288 |
| Q4437_08825 | hypothetical protein | -2.493092612 | 3.34E-05 | 0.000756996 |
| Q4437_08940 | GNAT family N-acetyltransferase | -2.071036627 | 0.00406587 | 0.033119591 |
| Q4437_09145 | DUF2947 domain-containing protein | -1.571587827 | 0.001413261 | 0.015293008 |
| Q4437_09160 | transcriptional regulator TyrR | -1.276110249 | 0.003280459 | 0.028398484 |
| Q4437_09220 | GNAT family N-acetyltransferase | -1.776275443 | 0.001224797 | 0.013655253 |
| Q4437_09225 | 6-carboxytetrahydropterin synthase QueD | -1.639039206 | 0.000646356 | 0.008223811 |
| Q4437_09240 | cold-shock protein | -6.44798703 | 0.002434933 | 0.022775908 |
| Q4437_09260 | methyl-accepting chemotaxis protein | 1.754704674 | 6.13E-05 | 0.001201881 |
| Q4437_09280 | hypothetical protein | 1.52385361 | 0.001117963 | 0.01272115 |
| Q4437_09285 | hypothetical protein | 1.351846611 | 0.001420902 | 0.01532608 |
| Q4437_09345 | TRAP transporter substrate-binding protein | 2.619670865 | 2.31E-07 | 1.20E-05 |
| Q4437_09355 | DUF2982 domain-containing protein | -2.039819321 | 1.33E-05 | 0.000359678 |
| Q4437_09380 | imelysin family protein | 1.723626999 | 0.000396901 | 0.005689338 |
| Q4437_09385 | di-heme oxidoredictase family protein | 2.348913252 | 8.15E-07 | 3.60E-05 |
| Q4437_09390 | imelysin family protein | 2.88472043 | 3.55E-10 | 4.12E-08 |
| Q4437_09645 | septation protein A | -1.800092956 | 0.001004669 | 0.011672665 |
| Q4437_09685 | EAL domain-containing protein | 1.886921085 | 1.36E-05 | 0.000366632 |
| Q4437_09725 | LysR family transcriptional regulator | -1.73494315 | 0.001269216 | 0.014044085 |
| Q4437_09730 | HlyD family secretion protein | -6.572140185 | 0.00143701 | 0.01532608 |
| Q4437_09830 | response regulator | -5.163625243 | 8.61E-11 | 1.23E-08 |
| Q4437_09835 | sensor histidine kinase | -1.935506126 | 3.30E-05 | 0.000750421 |
| Q4437_09840 | 4Fe-4S dicluster domain-containing protein | -12.36881279 | 1.57E-32 | 2.31E-29 |
| Q4437_09845 | polysulfide reductase NrfD | -12.62321559 | 2.34E-34 | 5.16E-31 |
| Q4437_09850 | tetrathionate reductase subunit A | -9.003132572 | 2.45E-44 | 1.08E-40 |
| Q4437_09855 | c-type cytochrome | -10.25851566 | 2.14E-18 | 1.31E-15 |
| Q4437_09860 | c-type cytochrome | -8.459982817 | 3.75E-26 | 4.14E-23 |
| Q4437_09930 | 30S ribosomal protein S1 | 1.198249332 | 0.003972963 | 0.032786228 |
| Q4437_09955 | DNA polymerase Y family protein | -2.071036627 | 0.00406587 | 0.033119591 |
| Q4437_09960 | error-prone DNA polymerase | -1.599712364 | 0.004522761 | 0.035216913 |
| Q4437_09965 | chemotaxis protein CheV | -1.379307474 | 0.001795867 | 0.018227019 |
| Q4437_10005 | hypothetical protein | 1.192766448 | 0.005613957 | 0.041177282 |
| Q4437_10150 | SEC-C metal-binding domain-containing protein | -2.188295705 | 5.96E-05 | 0.001177941 |
| Q4437_10275 | sigma-54 dependent transcriptional regulator | -1.249926747 | 0.003804684 | 0.031874154 |
| Q4437_10290 | electron transport complex subunit RsxA | -1.690572812 | 0.001136297 | 0.012896537 |
| Q4437_10370 | SDR family oxidoreductase | 1.41265299 | 0.00138955 | 0.015147808 |
| Q4437_10460 | hypothetical protein | -3.330313233 | 1.05E-05 | 0.000308725 |
| Q4437_10480 | AAA family ATPase | 1.203858197 | 0.004469242 | 0.035009903 |
| Q4437_10505 | hypothetical protein | 1.906339628 | 2.31E-05 | 0.000567336 |
| Q4437_10560 | methyl-accepting chemotaxis protein | -2.636408486 | 0.000495363 | 0.006750091 |
| Q4437_10585 | ribbon-helix-helix domain-containing protein | -1.818228077 | 0.00561466 | 0.041177282 |
| Q4437_10605 | YcgN family cysteine cluster protein | -2.227245679 | 3.95E-05 | 0.000863984 |
| Q4437_10610 | GlsB/YeaQ/YmgE family stress response membrane protein | 1.69909728 | 0.000192235 | 0.003120293 |
| Q4437_10625 | L-serine ammonia-lyase, iron-sulfur-dependent, subunit alpha | -1.52456017 | 0.003454774 | 0.029559742 |
| Q4437_10705 | acetyl-CoA carboxylase, carboxyltransferase subunit beta | 1.329054843 | 0.00170448 | 0.017541448 |
| Q4437_10810 | outer membrane protein transport protein | 1.229620393 | 0.004373891 | 0.034500574 |
| Q4437_10900 | protein phosphatase CheZ | -1.338262759 | 0.002146378 | 0.02087282 |
| Q4437_11010 | ATP-binding protein | -1.465310227 | 0.001662958 | 0.017154111 |
| Q4437_11075 | helix-turn-helix domain-containing protein | -1.833349877 | 3.38E-05 | 0.000760711 |
| Q4437_11470 | FAD:protein FMN transferase | 1.487858651 | 0.000788213 | 0.009586664 |
| Q4437_11495 | NADH:ubiquinone reductase (Na(+)-transporting) subunit B | 1.129081107 | 0.006889251 | 0.048665669 |
| Q4437_11550 | LON peptidase substrate-binding domain-containing protein | -2.217481989 | 0.00064876 | 0.008230681 |
| Q4437_11555 | outer membrane protein OmpK | 2.159555396 | 4.81E-07 | 2.26E-05 |
| Q4437_11580 | cysteine desulfurase sulfur acceptor subunit CsdE | -1.571867311 | 0.004764664 | 0.036712026 |
| Q4437_11585 | tRNA cyclic N6-threonylcarbamoyladenosine(37) synthase TcdA | -1.445186982 | 0.003239415 | 0.028153581 |
| Q4437_11605 | hypothetical protein | -1.400785182 | 0.003158082 | 0.027774765 |
| Q4437_11625 | DUF3413 domain-containing protein | -3.642679162 | 9.39E-14 | 2.64E-11 |
| Q4437_11630 | TDT family transporter | -1.67221372 | 0.004472386 | 0.035009903 |
| Q4437_11675 | glycerol-3-phosphate dehydrogenase | -3.082379405 | 2.22E-09 | 2.13E-07 |
| Q4437_11710 | cellulase family glycosylhydrolase | -2.917970503 | 2.38E-05 | 0.00057981 |
| Q4437_11775 | tetratricopeptide repeat protein | 1.967092591 | 5.39E-05 | 0.001101082 |
| Q4437_11785 | type II secretion system F family protein | 1.60962183 | 0.005549857 | 0.040905872 |
| Q4437_11790 | CpaF family protein | 1.380722666 | 0.002523983 | 0.023312522 |
| Q4437_11795 | AAA family ATPase | 1.447239582 | 0.006246459 | 0.044915502 |
| Q4437_11825 | Flp pilus assembly protein CpaB | 2.014826937 | 0.001317417 | 0.014468642 |
| Q4437_11890 | purine-nucleoside phosphorylase | 1.301625334 | 0.002557926 | 0.023527588 |
| Q4437_11970 | sodium-coupled multidrug efflux MATE transporter VmrA | -2.159555255 | 1.58E-06 | 6.21E-05 |
| Q4437_12140 | ABC transporter ATP-binding protein | -1.458943193 | 0.005716005 | 0.041712664 |
| Q4437_12185 | Fe(3+) ABC transporter substrate-binding protein | 2.814692589 | 2.57E-10 | 3.24E-08 |
| Q4437_12290 | transcriptional regulator OpaR | 2.17084146 | 5.17E-07 | 2.38E-05 |
| Q4437_12310 | pyruvate dehydrogenase complex transcriptional repressor PdhR | -1.851650363 | 3.60E-05 | 0.000798456 |
| Q4437_12360 | 50S ribosomal protein L19 | 1.311772295 | 0.001740852 | 0.01783263 |
| Q4437_12375 | 30S ribosomal protein S16 | 1.207455924 | 0.004317355 | 0.034159719 |
| Q4437_12500 | recombination regulator RecX | -2.558164524 | 0.004849114 | 0.037103708 |
| Q4437_12615 | signal peptidase I | -1.292350861 | 0.004435848 | 0.034847451 |
| Q4437_12625 | SoxR reducing system RseC family protein | -2.006788662 | 1.91E-05 | 0.000483669 |
| Q4437_12665 | DUF1107 domain-containing protein | 1.289656619 | 0.002013114 | 0.019883437 |
| Q4437_12715 | LysR family transcriptional regulator ArgP | -1.981843684 | 0.000558249 | 0.007291918 |
| Q4437_12720 | LysE/ArgO family amino acid transporter | -2.947033252 | 7.50E-06 | 0.000232577 |
| Q4437_12775 | SprT family zinc-dependent metalloprotease | -2.868965709 | 0.005247305 | 0.039265852 |
| Q4437_12850 | glutaminase B | -1.639472276 | 0.001942548 | 0.019359703 |
| Q4437_12855 | DNA methylase | 1.246464549 | 0.004600298 | 0.035694759 |
| Q4437_13020 | PTS sugar transporter subunit IIB | -1.958433231 | 0.000661742 | 0.008347409 |
| Q4437_13025 | chitin oligosaccharide deacetylase | 1.731905061 | 0.000361485 | 0.00521555 |
| Q4437_13075 | DUF2061 domain-containing protein | -3.956645429 | 0.000575412 | 0.0074719 |
| Q4437_13085 | hypothetical protein | -3.215396981 | 1.38E-07 | 8.49E-06 |
| Q4437_13100 | ornithine carbamoyltransferase | 1.425435232 | 0.003618164 | 0.030485107 |
| Q4437_13195 | ribosome hibernation promoting factor | 1.560384045 | 0.000521577 | 0.006936028 |
| Q4437_13250 | PhoH family protein | 1.495833566 | 0.000460686 | 0.006336233 |
| Q4437_13660 | N-acetyl-gamma-glutamyl-phosphate reductase | 3.745513662 | 8.40E-08 | 5.98E-06 |
| Q4437_13675 | PadR family transcriptional regulator | -2.799864219 | 2.46E-09 | 2.31E-07 |
| Q4437_13730 | 30S ribosomal protein S12 | 1.133150403 | 0.006573406 | 0.046802838 |
| Q4437_13790 | glutathione-regulated potassium-efflux system ancillary protein KefG | -1.452467561 | 0.00468342 | 0.036250119 |
| Q4437_13880 | nitric oxide-sensing transcriptional repressor NsrR | -2.17701927 | 0.000124729 | 0.002185237 |
| Q4437_14040 | DMT family transporter | -2.174140222 | 0.001356148 | 0.014820283 |
| Q4437_14045 | 2,3-bisphosphoglycerate-independent phosphoglycerate mutase | -1.192760517 | 0.004967407 | 0.037943084 |
| Q4437_14115 | SPFH domain-containing protein | -1.386505882 | 0.001528085 | 0.016004974 |
| Q4437_14185 | superoxide dismutase | 1.444943621 | 0.000889162 | 0.010440557 |
| Q4437_14225 | DUF4212 domain-containing protein | 4.250330865 | 6.46E-11 | 9.83E-09 |
| Q4437_14230 | sodium:solute symporter family protein | 2.194771735 | 1.51E-06 | 6.12E-05 |
| Q4437_14245 | class II fumarate hydratase | 2.052042578 | 5.45E-05 | 0.001108081 |
| Q4437_14260 | DUF294 nucleotidyltransferase-like domain-containing protein | 2.527805593 | 1.13E-07 | 7.56E-06 |
| Q4437_14265 | 3'-5' exonuclease | 2.693970797 | 0.000178139 | 0.002902148 |
| Q4437_14270 | acetate--CoA ligase | 2.847493903 | 1.45E-10 | 1.95E-08 |
| Q4437_14280 | acetyl-CoA carboxylase biotin carboxyl carrier protein | 1.181208101 | 0.006510895 | 0.046438772 |
| Q4437_14285 | acetyl-CoA carboxylase biotin carboxylase subunit | 1.262573661 | 0.002688598 | 0.024474555 |
| Q4437_14295 | tRNA dihydrouridine synthase DusB | -1.649221009 | 0.000197803 | 0.00317563 |
| Q4437_14385 | lysine decarboxylase CadA | -3.037623306 | 0.002364823 | 0.022554288 |
| Q4437_14395 | lysine decarboxylation/transport transcriptional activator CadC | -1.446967341 | 0.002696242 | 0.02449364 |
| Q4437_14425 | chromosome partitioning protein ParB | -4.766293735 | 3.63E-17 | 1.60E-14 |
| Q4437_14445 | hypothetical protein | -2.195924903 | 5.20E-05 | 0.001093129 |
| Q4437_14500 | hypothetical protein | 1.931034048 | 9.38E-05 | 0.001740357 |
| Q4437_14505 | hypothetical protein | 1.721689661 | 0.000160708 | 0.002718488 |
| Q4437_14545 | 50S ribosomal protein L11 | 1.221692125 | 0.003407551 | 0.029269142 |
| Q4437_14695 | MATE family efflux transporter DinF | -2.44809435 | 4.87E-05 | 0.001037747 |
| Q4437_14980 | hypothetical protein | -1.371073557 | 0.003387961 | 0.029157602 |
| Q4437_15005 | 7-cyano-7-deazaguanine/7-aminomethyl-7-deazaguanine transporter | -2.36033798 | 1.18E-05 | 0.000330869 |
| Q4437_15075 | LysR family transcriptional regulator | -2.075078688 | 0.000604137 | 0.00775368 |
| Q4437_15105 | thiamine phosphate synthase | -2.080395106 | 0.001608974 | 0.016699787 |
| Q4437_15110 | phosphomethylpyrimidine synthase ThiC | -2.394812791 | 1.07E-05 | 0.000308725 |
| Q4437_15115 | fluoride efflux transporter CrcB | -2.014242821 | 0.00360343 | 0.030419014 |
| Q4437_15245 | hemolysin III family protein | -1.539606616 | 0.002916468 | 0.026118069 |
| Q4437_15355 | F0F1 ATP synthase subunit gamma | 1.195738869 | 0.004119382 | 0.033248759 |
| Q4437_15360 | F0F1 ATP synthase subunit alpha | 1.199453544 | 0.003959883 | 0.032758755 |
| Q4437_15365 | F0F1 ATP synthase subunit delta | 1.173984311 | 0.005048028 | 0.038228203 |
| Q4437_15370 | F0F1 ATP synthase subunit B | 1.396224579 | 0.00088882 | 0.010440557 |
| Q4437_15375 | F0F1 ATP synthase subunit C | 1.169240771 | 0.005704039 | 0.041694258 |
| Q4437_15380 | F0F1 ATP synthase subunit A | 1.399490405 | 0.000855156 | 0.010269373 |
| Q4437_15385 | F0F1 ATP synthase subunit I | 1.133554343 | 0.006602045 | 0.046852512 |
| Q4437_15435 | 50S ribosomal protein L34 | 1.251404904 | 0.003202126 | 0.028050368 |
| Q4437_15450 | amino acid ABC transporter substrate-binding protein | -1.274759829 | 0.003025508 | 0.026876496 |
| Q4437_15545 | DUF3360 domain-containing protein | 1.655745576 | 0.001822716 | 0.018414856 |
| Q4437_15575 | recombinase family protein | -1.708239002 | 0.000252447 | 0.003843283 |
| Q4437_15595 | heme utilization protein HutZ | 1.428119469 | 0.005127009 | 0.038561746 |
| Q4437_15600 | heme utilization cystosolic carrier protein HutX | 1.605835197 | 0.006984897 | 0.049105609 |
| Q4437_15605 | heme anaerobic degradation radical SAM methyltransferase ChuW/HutW | 2.513861403 | 0.000112106 | 0.002020201 |
| Q4437_15610 | energy transducer TonB | 3.973071734 | 1.06E-05 | 0.000308725 |
| Q4437_15625 | hemin ABC transporter substrate-binding protein | 2.405666853 | 0.00288962 | 0.026008334 |
| Q4437_15725 | DUF4382 domain-containing protein | 1.485109378 | 0.001154344 | 0.013034348 |
| Q4437_15745 | DUF4156 domain-containing protein | -2.720980168 | 0.000280171 | 0.004178902 |
| Q4437_15790 | LysR family transcriptional regulator | -2.071036627 | 0.00406587 | 0.033119591 |
| Q4437_15800 | alkene reductase | -6.312135043 | 0.004174171 | 0.033446394 |
| Q4437_15885 | hypothetical protein | -2.043986722 | 0.00202491 | 0.019955309 |
| Q4437_15940 | UTRA domain-containing protein | -3.325284436 | 0.000506526 | 0.006838872 |
| Q4437_15975 | hypothetical protein | 1.780182274 | 6.31E-05 | 0.001233182 |
| Q4437_15980 | hypothetical protein | 1.867560372 | 2.03E-05 | 0.000508583 |
| Q4437_16025 | redox-sensitive transcriptional activator SoxR | -1.445186982 | 0.003239415 | 0.028153581 |
| Q4437_16040 | N-acetyltransferase DgcN | 1.732033159 | 0.005019824 | 0.038182372 |
| Q4437_16055 | hypothetical protein | 1.933354829 | 1.74E-05 | 0.000449513 |
| Q4437_16095 | outer membrane beta-barrel protein | -2.211491261 | 0.001065499 | 0.01228245 |
| Q4437_16100 | LysR substrate-binding domain-containing protein | -1.60013288 | 0.001078545 | 0.012368251 |
| Q4437_16120 | acyl-CoA synthetase | -4.032542524 | 5.52E-06 | 0.00018062 |
| Q4437_16125 | nicotinate phosphoribosyltransferase | -3.686600484 | 2.67E-11 | 4.37E-09 |
| Q4437_16130 | NUDIX domain-containing protein | -3.190846254 | 9.91E-09 | 8.93E-07 |
| Q4437_16135 | nicotinamidase | -1.899081855 | 0.000147997 | 0.002532592 |
| Q4437_16140 | ankyrin repeat domain-containing protein | -6.572140185 | 0.00143701 | 0.01532608 |
| Q4437_16155 | DNA-binding transcriptional regulator YciT | -1.465656505 | 0.003045054 | 0.026995812 |
| Q4437_16190 | nitronate monooxygenase | 1.225263373 | 0.004783589 | 0.036793634 |
| Q4437_16220 | LysR family transcriptional regulator | -2.239993223 | 5.38E-05 | 0.001101082 |
| Q4437_16230 | leukocidin family pore-forming toxin | -1.650939287 | 0.006035825 | 0.043757252 |
| Q4437_16240 | chaperonin GroEL | -2.838021791 | 1.94E-09 | 1.90E-07 |
| Q4437_16245 | co-chaperone GroES | -2.454879894 | 3.10E-05 | 0.000717712 |
| Q4437_16260 | LruC domain-containing protein | -3.874779834 | 0.000884859 | 0.010440557 |
| Q4437_16270 | DUF3012 domain-containing protein | -1.332333489 | 0.004271698 | 0.033929625 |
| Q4437_16380 | hypothetical protein | -2.959438137 | 9.57E-10 | 1.03E-07 |
| Q4437_16405 | LysR family transcriptional regulator | -2.023927809 | 0.005290723 | 0.039294532 |
| Q4437_16420 | outer membrane beta-barrel protein | 1.840525437 | 1.60E-05 | 0.000417086 |
| Q4437_16525 | efflux transporter outer membrane subunit | -1.650939287 | 0.006035825 | 0.043757252 |
| Q4437_16545 | carbonic anhydrase family protein | 2.044078642 | 0.00140429 | 0.015270787 |
| Q4437_16555 | hypothetical protein | 1.549543026 | 0.001830875 | 0.018455048 |
| Q4437_16570 | LysR substrate-binding domain-containing protein | -2.115907246 | 0.001279883 | 0.014091475 |
| Q4437_16585 | hypothetical protein | -2.351916569 | 0.000411667 | 0.005844082 |
| Q4437_16590 | M66 family metalloprotease | -1.33736932 | 0.004812954 | 0.03695512 |
| Q4437_16655 | methyl-accepting chemotaxis protein | 1.215112076 | 0.005047904 | 0.038228203 |
| Q4437_16685 | DUF2913 family protein | -3.008086189 | 0.000419448 | 0.005878934 |
| Q4437_16790 | multidrug efflux MFS transporter EmrD | -3.674857705 | 5.42E-13 | 1.26E-10 |
| Q4437_16800 | porin | 2.072217633 | 1.19E-06 | 5.02E-05 |
| Q4437_16855 | energy transducer TonB | 1.516578116 | 0.004513216 | 0.035204682 |
| Q4437_16870 | MotA/TolQ/ExbB proton channel family protein | 1.656084023 | 0.000175727 | 0.002894901 |
| Q4437_16875 | DUF3450 domain-containing protein | 2.615756423 | 1.41E-07 | 8.53E-06 |
| Q4437_16880 | TonB-dependent siderophore enterobactin receptor PeuA | 1.697035199 | 0.000117997 | 0.002075534 |
| Q4437_16885 | histidine kinase sensor domain-containing protein | 1.780538918 | 0.000440624 | 0.006117473 |
| Q4437_16905 | L,D-transpeptidase family protein | -1.818228077 | 0.00561466 | 0.041177282 |
| Q4437_17070 | PLP-dependent aminotransferase family protein | -1.584561987 | 0.003962214 | 0.032758755 |
| Q4437_17170 | MDR family MFS transporter | -2.793749451 | 2.22E-07 | 1.19E-05 |
| Q4437_17175 | HlyD family secretion protein | -3.09554512 | 1.67E-09 | 1.68E-07 |
| Q4437_17180 | outer membrane protein OmpW | 1.853920083 | 1.32E-05 | 0.000359678 |
| Q4437_17215 | siderophore-interacting protein | 1.473083708 | 0.002614377 | 0.023996824 |
| Q4437_17410 | YitT family protein | -1.860826596 | 0.00023413 | 0.003665547 |
| Q4437_17450 | DUF413 domain-containing protein | -1.410115349 | 0.002953275 | 0.026352656 |
| Q4437_17455 | DUF3081 domain-containing protein | 1.775315163 | 4.93E-05 | 0.001042414 |
| Q4437_17735 | bifunctional proline dehydrogenase/L-glutamate gamma-semialdehyde dehydrogenase PutA | 1.195363385 | 0.004118283 | 0.033248759 |
| Q4437_17740 | 1-pyrroline-5-carboxylate dehydrogenase | 1.260289439 | 0.002650148 | 0.024174387 |
| Q4437_17815 | carboxymuconolactone decarboxylase family protein | -2.792786913 | 6.40E-05 | 0.001245384 |
| Q4437_17860 | FCD domain-containing protein | -1.31947259 | 0.00706253 | 0.049493764 |
| Q4437_17890 | 6-phospho-beta-glucosidase | -2.504205999 | 0.000511792 | 0.00686778 |
| Q4437_17945 | MarR family transcriptional regulator | -3.458274423 | 3.58E-06 | 0.000123401 |
| Q4437_18045 | MFS transporter | 3.298298305 | 0.001084588 | 0.012405327 |
| Q4437_18050 | siderophore biosynthesis protein PsvB | 2.534051613 | 0.000511792 | 0.00686778 |
| Q4437_18060 | TonB-dependent siderophore receptor | 2.417818305 | 1.66E-07 | 9.58E-06 |
| Q4437_18065 | TonB-dependent siderophore vibrioferrin receptor PvuA | 2.483880464 | 1.83E-07 | 1.01E-05 |
| Q4437_18140 | glycosidase | 1.954965408 | 0.000151919 | 0.002589669 |
| Q4437_18150 | endonuclease | 1.339218076 | 0.002511409 | 0.023293845 |
| Q4437_18200 | ribosome small subunit-dependent GTPase A | -1.910222008 | 4.53E-05 | 0.000979411 |
| Q4437_18235 | glycogen/starch/alpha-glucan phosphorylase | -1.505231429 | 0.001954481 | 0.019396432 |
| Q4437_18250 | monofunctional biosynthetic peptidoglycan transglycosylase | -2.252597762 | 0.00022797 | 0.003581803 |
| Q4437_18255 | alpha-amylase | -1.710049652 | 0.000537329 | 0.007089557 |
| Q4437_18300 | LysR family transcriptional regulator | -2.192601783 | 0.000513333 | 0.00686778 |
| Q4437_18310 | polysaccharide biosynthesis tyrosine autokinase | 1.377142971 | 0.001410537 | 0.015293008 |
| Q4437_18340 | patatin family protein | -2.372334947 | 0.000214401 | 0.003392756 |
| Q4437_18365 | D-alanyl-D-alanine carboxypeptidase family protein | 1.521722508 | 0.001102015 | 0.012572086 |
| Q4437_18370 | alpha-amylase family glycosyl hydrolase | -2.951784144 | 0.000589 | 0.00762591 |
| Q4437_18385 | putative quinol monooxygenase | -1.697348606 | 0.002413127 | 0.022775908 |
| Q4437_18390 | LysR family transcriptional regulator | -1.934636623 | 0.000784949 | 0.009573346 |
| Q4437_18405 | ferredoxin--NADP reductase | -2.027418996 | 1.26E-05 | 0.000347889 |
| Q4437_18410 | RDD family protein | -4.107633797 | 0.000248027 | 0.003789058 |
| Q4437_18420 | AI-2E family transporter | 1.51875228 | 0.001508015 | 0.015900115 |
| Q4437_18425 | potassium channel family protein | 1.969059052 | 0.002892433 | 0.026008334 |
| Q4437_18430 | HlyD family secretion protein | 2.135299865 | 0.000242489 | 0.00373028 |
| Q4437_18440 | TolC family protein | 1.309037654 | 0.004271698 | 0.033929625 |
| Q4437_18470 | type I restriction enzyme HsdR N-terminal domain-containing protein | 2.06451907 | 1.66E-06 | 6.43E-05 |
| Q4437_18480 | DUF2799 domain-containing protein | -2.366802956 | 2.53E-05 | 0.000604552 |
| Q4437_18485 | fructose-6-phosphate aldolase | -3.325284436 | 0.000506526 | 0.006838872 |
| Q4437_18490 | glycyl-radical enzyme activating protein | -4.107633797 | 0.000248027 | 0.003789058 |
| Q4437_18660 | flagellar protein export ATPase FliI | -2.023927809 | 0.005290723 | 0.039294532 |
| Q4437_18670 | serine protease | 1.716608603 | 0.000444631 | 0.006153756 |
| Q4437_18695 | cytochrome b562 | 1.299138667 | 0.002544244 | 0.023450598 |
| Q4437_18775 | spore coat U domain-containing protein | 1.833626868 | 0.001512582 | 0.015900115 |
| Q4437_18815 | FMN-dependent L-lactate dehydrogenase LldD | 2.205443393 | 3.57E-07 | 1.71E-05 |
| Q4437_18820 | L-lactate permease | 1.356282005 | 0.001333826 | 0.014612515 |
| Q4437_18850 | methyl-accepting chemotaxis protein | -1.877630006 | 0.000262593 | 0.003956824 |
| Q4437_19060 | LuxR C-terminal-related transcriptional regulator | 2.264464363 | 7.24E-05 | 0.001384158 |
| Q4437_19070 | calcium-binding protein | 2.410896481 | 5.74E-05 | 0.00114389 |
| Q4437_19080 | HlyD family type I secretion periplasmic adaptor subunit | 1.469422264 | 0.002256005 | 0.021747299 |
| Q4437_19120 | TonB-dependent siderophore receptor | 1.59248504 | 0.000455248 | 0.006280993 |
| Q4437_19150 | EAL domain-containing protein | -2.480695989 | 0.006966163 | 0.049052007 |
| Q4437_19180 | AraC family transcriptional regulator | -2.149916519 | 7.17E-06 | 0.000226172 |
| Q4437_19195 | fructose-specific PTS transporter subunit EIIC | -1.37807915 | 0.003531724 | 0.030043472 |
| Q4437_19215 | NADP-dependent oxidoreductase | -2.184362667 | 4.78E-05 | 0.001029823 |
| Q4437_19220 | TetR/AcrR family transcriptional regulator | -2.459476898 | 3.52E-07 | 1.71E-05 |
| Q4437_19240 | glycosyltransferase | 3.116442259 | 1.10E-07 | 7.53E-06 |
| Q4437_19245 | putative capsular polysaccharide synthesis family protein | 2.792725056 | 1.24E-06 | 5.15E-05 |
| Q4437_19250 | O-antigen ligase family protein | 2.459241169 | 5.31E-05 | 0.001101082 |
| Q4437_19255 | glycosyltransferase | 3.019222678 | 1.30E-07 | 8.46E-06 |
| Q4437_19260 | putative capsular polysaccharide synthesis family protein | 3.600467171 | 2.86E-08 | 2.43E-06 |
| Q4437_19270 | polysaccharide biosynthesis/export family protein | 2.19281485 | 0.001019268 | 0.011780287 |
| Q4437_19275 | outer membrane beta-barrel protein | 2.67842903 | 1.38E-07 | 8.49E-06 |
| Q4437_19280 | undecaprenyl-phosphate glucose phosphotransferase | 2.557198205 | 2.63E-07 | 1.32E-05 |
| Q4437_19305 | hypothetical protein | -3.427332345 | 4.66E-06 | 0.00015597 |
| Q4437_19335 | hypothetical protein | -2.237645676 | 0.003904919 | 0.032505923 |
| Q4437_19345 | type I-F CRISPR-associated endoribonuclease Cas6/Csy4 | 2.595121044 | 2.25E-06 | 8.33E-05 |
| Q4437_19355 | type I-F CRISPR-associated protein Csy2 | 1.762234496 | 0.000115775 | 0.002061067 |
| Q4437_19455 | hypothetical protein | -2.832126106 | 0.001173602 | 0.013184363 |
| Q4437_19460 | molecular chaperone | -6.44798703 | 0.002434933 | 0.022775908 |
| Q4437_19475 | ATP-dependent exonuclease | -3.874779834 | 0.000884859 | 0.010440557 |
| Q4437_19500 | FHIPEP family type III secretion protein | -1.928901076 | 0.004060649 | 0.033119591 |
| Q4437_19610 | VPA1331 family putative T3SS effector | -2.400360138 | 0.001591547 | 0.016572364 |
| Q4437_19660 | IS30 family transposase | -3.114006804 | 1.44E-05 | 0.000386194 |
| Q4437_19720 | hypothetical protein | -3.919049078 | 5.57E-07 | 2.53E-05 |
| Q4437_19725 | zinc ABC transporter substrate-binding protein | -5.103058677 | 2.51E-07 | 1.27E-05 |
| Q4437_19730 | metal ABC transporter permease | -3.565013506 | 0.000116677 | 0.002063427 |
| Q4437_19745 | hypothetical protein | -2.055909045 | 0.002817474 | 0.025490057 |
| Q4437_19785 | adenosine deaminase | -1.643270086 | 0.003019951 | 0.026876496 |
| Q4437_19790 | adenosine deaminase | -2.983141871 | 4.11E-05 | 0.000893161 |
| Q4437_19800 | cold-shock protein | -3.754290112 | 4.27E-14 | 1.35E-11 |
| Q4437_19815 | CDF family Co(II)/Ni(II) efflux transporter DmeF | -2.837587732 | 1.08E-06 | 4.64E-05 |
| Q4437_19840 | MFS transporter | -6.312135043 | 0.004174171 | 0.033446394 |
| Q4437_19855 | phosphoethanolamine--lipid A transferase | -1.351354218 | 0.00352744 | 0.030043472 |
| Q4437_19860 | diacylglycerol kinase | -2.179056866 | 0.005286278 | 0.039294532 |
| Q4437_19865 | glycine zipper family protein | -2.46496139 | 6.54E-05 | 0.001263861 |
| Q4437_19880 | SDR family oxidoreductase | -2.43236506 | 9.59E-06 | 0.000289902 |
| Q4437_19890 | hypothetical protein | -1.539969205 | 0.003108397 | 0.027502146 |
| Q4437_20030 | hypothetical protein | -3.189887926 | 3.21E-05 | 0.000737206 |
| Q4437_20090 | glyceraldehyde-3-phosphate dehydrogenase | 1.953772994 | 4.48E-06 | 0.00015083 |
| Q4437_20105 | MATE family efflux transporter | -1.5380857 | 0.002201389 | 0.021305931 |
| Q4437_20115 | hypothetical protein | -1.385070956 | 0.00658314 | 0.046802838 |
| Q4437_20125 | GIY-YIG nuclease family protein | 1.571998488 | 0.00217445 | 0.021099332 |
| Q4437_20140 | MarR family transcriptional regulator | -3.20491061 | 6.75E-06 | 0.000215851 |
| Q4437_20190 | protein translocase subunit SecD | -3.420421495 | 1.24E-09 | 1.30E-07 |
| Q4437_20195 | protein translocase subunit SecF | -1.701448281 | 0.001239941 | 0.013771912 |
| Q4437_20210 | SDR family oxidoreductase | 2.735614184 | 7.43E-08 | 5.56E-06 |
| Q4437_20215 | acetyl-CoA C-acetyltransferase | 2.054572527 | 1.16E-05 | 0.000330589 |
| Q4437_20220 | phasin family protein | 2.943681354 | 2.65E-10 | 3.25E-08 |
| Q4437_20225 | class I poly(R)-hydroxyalkanoic acid synthase | 2.366520884 | 1.38E-07 | 8.49E-06 |
| Q4437_20255 | ferredoxin-type protein NapF | 1.779196772 | 0.00191984 | 0.019176684 |
| Q4437_20290 | multidrug efflux RND transporter periplasmic adaptor subunit VmeY | 2.07697214 | 1.66E-05 | 0.000431601 |
| Q4437_20295 | multidrug efflux RND transporter permease subunit VmeZ | 1.490370831 | 0.000616918 | 0.007894763 |
| Q4437_20315 | OmpA family protein | 2.049081063 | 1.55E-06 | 6.19E-05 |
| Q4437_20345 | sugar-binding transcriptional regulator | -1.518153182 | 0.001217731 | 0.013610836 |
| Q4437_20390 | divalent metal cation transporter | -4.352210134 | 2.68E-17 | 1.32E-14 |
| Q4437_20400 | H(+)/Cl(-) exchange transporter ClcA | -2.401937768 | 1.86E-05 | 0.000474314 |
| Q4437_20465 | long-chain fatty acid--CoA ligase | 2.169889337 | 0.000284245 | 0.004197128 |
| Q4437_20480 | ABC transporter substrate-binding protein | 2.086780349 | 0.000211708 | 0.003362204 |
| Q4437_20550 | sugar O-acetyltransferase | 1.453378033 | 0.003841393 | 0.032120735 |
| Q4437_20555 | mechanosensitive ion channel | -2.036676981 | 0.006127713 | 0.044133527 |
| Q4437_20830 | DUF5666 domain-containing protein | -2.472138076 | 3.30E-06 | 0.000114686 |
| Q4437_20885 | molybdopterin-dependent oxidoreductase | -3.695638337 | 0.002133716 | 0.020795492 |
| Q4437_20945 | glutathione S-transferase | -2.139491054 | 4.90E-05 | 0.00103951 |
| Q4437_20980 | serine/threonine-protein kinase | 1.92670556 | 1.18E-05 | 0.000330869 |
| Q4437_20985 | type VI secretion system-associated FHA domain protein TagH | 2.426102518 | 4.80E-08 | 3.85E-06 |
| Q4437_20990 | type VI secretion system lipoprotein TssJ | 2.121139462 | 1.06E-05 | 0.000308725 |
| Q4437_20995 | type VI secretion system baseplate subunit TssK | 2.454240661 | 1.11E-07 | 7.53E-06 |
| Q4437_21000 | type VI secretion system protein TssL, long form | 2.045479247 | 8.55E-06 | 0.000260433 |
| Q4437_21005 | type VI secretion system membrane subunit TssM | 2.093771094 | 1.45E-06 | 5.93E-05 |
| Q4437_21010 | type VI secretion system-associated protein TagF | 2.135942587 | 1.46E-05 | 0.000387185 |
| Q4437_21015 | protein phosphatase 2C domain-containing protein | 2.078883528 | 2.05E-05 | 0.000511289 |
| Q4437_21020 | type VI secretion system protein TssA | 2.428311995 | 3.52E-07 | 1.71E-05 |
| Q4437_21025 | type VI secretion system contractile sheath small subunit | 1.790749681 | 0.000225103 | 0.003549396 |
| Q4437_21030 | type VI secretion system contractile sheath large subunit | 2.075917319 | 1.78E-06 | 6.84E-05 |
| Q4437_21035 | type VI secretion system contractile sheath large subunit | 1.742765705 | 0.000421672 | 0.0058914 |
| Q4437_21040 | type VI secretion system accessory protein TagJ | 1.960484367 | 0.001496038 | 0.015839343 |
| Q4437_21050 | type VI secretion system baseplate subunit TssF | 2.045037707 | 2.68E-05 | 0.00063327 |
| Q4437_21055 | type VI secretion system baseplate subunit TssG | 1.9986149 | 0.000784949 | 0.009573346 |
| Q4437_21060 | type VI secretion system ATPase TssH | 2.140428493 | 5.58E-06 | 0.000181304 |
| Q4437_21065 | type VI secretion system tube protein Hcp | 3.474338886 | 3.81E-14 | 1.29E-11 |
| Q4437_21070 | type VI secretion system tip protein TssI/VgrG | 2.431622621 | 9.39E-08 | 6.58E-06 |
| Q4437_21075 | PAAR domain-containing protein | 2.59222817 | 0.000103342 | 0.001885352 |
| Q4437_21080 | transhydrogenase beta subunit | 1.884149034 | 0.000175416 | 0.002894901 |
| Q4437_21110 | lipocalin family protein | 1.896738862 | 0.000241346 | 0.003725674 |
| Q4437_21115 | helix-turn-helix transcriptional regulator | -2.924252133 | 4.15E-06 | 0.000140807 |
| Q4437_21150 | DUF3763 domain-containing protein | -1.580069077 | 0.000468691 | 0.006426301 |
| Q4437_21155 | ATPase RavA stimulator ViaA | -1.412905626 | 0.001876502 | 0.018828993 |
| Q4437_21180 | AzlD domain-containing protein | -2.868965709 | 0.005247305 | 0.039265852 |
| Q4437_21195 | methyl-accepting chemotaxis protein | -1.706805895 | 0.000270971 | 0.004069173 |
| Q4437_21260 | hypothetical protein | -2.638474691 | 9.35E-05 | 0.001740357 |
| Q4437_21265 | nucleoside diphosphate kinase regulator | 2.099979446 | 2.67E-06 | 9.57E-05 |
| Q4437_21300 | anaerobic C4-dicarboxylate transporter | 1.35927701 | 0.001973191 | 0.019532818 |
| Q4437_21310 | TonB-dependent receptor | 1.915892379 | 5.98E-05 | 0.001177941 |
| Q4437_21325 | nitrous oxide-stimulated promoter family protein | -2.04766451 | 0.000733197 | 0.009092875 |
| Q4437_21330 | MATE family efflux transporter | -4.244306559 | 0.000109596 | 0.001991213 |
| Q4437_21340 | MFS transporter | -2.623586367 | 1.27E-07 | 8.39E-06 |
| Q4437_21355 | DUF1501 domain-containing protein | 2.132369139 | 0.000604137 | 0.00775368 |
| Q4437_21360 | DUF1800 domain-containing protein | 2.391143069 | 0.000126706 | 0.002211101 |
| Q4437_21395 | formate dehydrogenase accessory sulfurtransferase FdhD | -2.701638583 | 0.002370371 | 0.022554288 |
| Q4437_21400 | LysR family transcriptional regulator | -3.541118343 | 7.78E-08 | 5.72E-06 |
| Q4437_21405 | FdhF/YdeP family oxidoreductase | -1.983318747 | 3.28E-05 | 0.000750179 |
| Q4437_21420 | ABC transporter substrate-binding protein | -2.293830836 | 3.99E-06 | 0.000136414 |
| Q4437_21455 | metalloregulator ArsR/SmtB family transcription factor | -4.303461725 | 1.33E-08 | 1.17E-06 |
| Q4437_21460 | YeeE/YedE family protein | -3.440369176 | 2.14E-07 | 1.17E-05 |
| Q4437_21520 | NirD/YgiW/YdeI family stress tolerance protein | 1.507944119 | 0.00087817 | 0.010440557 |
| Q4437_21615 | hypothetical protein | 1.498248833 | 0.000858519 | 0.010271978 |
| Q4437_21780 | TonB-dependent hemoglobin/transferrin/lactoferrin family receptor | 1.689298409 | 0.000708766 | 0.008889772 |
| Q4437_21855 | 2OG-Fe(II) oxygenase family protein | 1.5199563 | 0.00040853 | 0.005818262 |
| Q4437_21860 | DUF2238 domain-containing protein | -2.462073364 | 7.26E-06 | 0.000227201 |
| Q4437_21875 | SgrR family transcriptional regulator | -1.406963187 | 0.002493709 | 0.023246065 |
| Q4437_21915 | hypothetical protein | -1.697025964 | 0.000161757 | 0.002725793 |
| Q4437_21920 | TerC/Alx family metal homeostasis membrane protein | -2.108025582 | 5.67E-05 | 0.00114389 |
| Q4437_21935 | DUF1097 domain-containing protein | 1.581180342 | 0.000283688 | 0.004197128 |
| Q4437_22010 | inosine/guanosine kinase | -2.402416361 | 1.16E-06 | 4.94E-05 |
| Q4437_22030 | AraC family transcriptional regulator | -3.543612061 | 3.11E-07 | 1.54E-05 |
| Q4437_22040 | lysoplasmalogenase | -1.692942691 | 0.005682197 | 0.041603479 |
| Q4437_22060 | MFS transporter | -6.44798703 | 0.002434933 | 0.022775908 |
| Q4437_22110 | fused PTS fructose transporter subunit IIA/HPr protein | -1.779237028 | 0.001614421 | 0.016699787 |
| Q4437_22115 | 1-phosphofructokinase | -2.558164524 | 0.004849114 | 0.037103708 |
| Q4437_22130 | multidrug efflux RND transporter permease subunit VmeV | -4.57868303 | 5.13E-20 | 3.77E-17 |
| Q4437_22135 | multidrug efflux RND transporter periplasmic adaptor subunit VmeU | -3.646431529 | 4.52E-12 | 9.29E-10 |
| Q4437_22140 | multidrug efflux RND transporter periplasmic adaptor subunit VmeT | -4.242638734 | 3.81E-15 | 1.40E-12 |
| Q4437_22205 | LysR family transcriptional regulator | -1.681257974 | 0.002073218 | 0.020340575 |
| Q4437_22320 | RES family NAD+ phosphorylase | -2.701638583 | 0.002370371 | 0.022554288 |
| Q4437_22340 | YbaK/EbsC family protein | 2.09376199 | 3.28E-06 | 0.000114686 |
| Q4437_22390 | M4 family metallopeptidase | -2.400360138 | 0.001591547 | 0.016572364 |
| Q4437_22420 | type IV pilin MshA | -6.983382137 | 0.000193974 | 0.003125533 |
| Q4437_22470 | transporter substrate-binding domain-containing protein | -2.485263047 | 9.06E-05 | 0.001708664 |
| Q4437_22480 | LysR family transcriptional regulator | -1.77883728 | 0.00701327 | 0.049226687 |
| Q4437_22485 | LysR family transcriptional regulator | -3.671425923 | 5.75E-05 | 0.00114389 |
| Q4437_22530 | CpaF family protein | -2.480695989 | 0.006966163 | 0.049052007 |
| Q4437_22565 | LysR family transcriptional regulator | -2.655700029 | 0.000178139 | 0.002902148 |
| Q4437_22575 | M6 family metalloprotease domain-containing protein | -3.363383193 | 7.98E-06 | 0.000244763 |
| Q4437_22660 | HAD family hydrolase | 2.000799115 | 0.003217043 | 0.028125241 |
| Q4437_22670 | NirD/YgiW/YdeI family stress tolerance protein | -2.04766451 | 0.000733197 | 0.009092875 |
| Q4437_22730 | LysR family transcriptional regulator | -2.116655702 | 0.003128224 | 0.027622221 |
| Q4437_22740 | arylsulfatase | 2.286012435 | 1.61E-06 | 6.29E-05 |
| Q4437_22745 | arylsulfatase | -1.846738281 | 0.00648527 | 0.04633085 |
| Q4437_22760 | hypothetical protein | 1.677386767 | 0.001810629 | 0.018334694 |
| Q4437_22830 | iron chelate ABC transporter ATP-binding protein VctC | 1.504884 | 0.002916468 | 0.026118069 |
| Q4437_22835 | iron chelate uptake ABC transporter permease subunit VctG | 1.881227787 | 0.0014637 | 0.015571652 |
| Q4437_22840 | iron chelate uptake ABC transporter permease subunit VctD | 3.030386935 | 1.71E-07 | 9.69E-06 |
| Q4437_22845 | siderophore ABC transporter substrate-binding protein | 3.696306431 | 9.55E-14 | 2.64E-11 |
| Q4437_22900 | electron transfer flavoprotein-ubiquinone oxidoreductase | 1.6640145 | 0.000855975 | 0.010269373 |
| Q4437_22905 | glutathione S-transferase family protein | 1.460276071 | 0.006056022 | 0.043831704 |
| Q4437_22955 | heme o synthase | -2.554045223 | 1.56E-06 | 6.19E-05 |
| Q4437_22965 | cytochrome o ubiquinol oxidase subunit III | -3.678952083 | 2.95E-11 | 4.65E-09 |
| Q4437_22970 | cytochrome o ubiquinol oxidase subunit I | -3.355867092 | 5.63E-12 | 1.04E-09 |
| Q4437_22975 | ubiquinol oxidase subunit II | -3.340753976 | 3.66E-08 | 2.99E-06 |
| Q4437_22995 | enoyl-CoA hydratase | 1.95912698 | 0.004060649 | 0.033119591 |
| Q4437_23045 | DMT family transporter | -2.115907246 | 0.001279883 | 0.014091475 |
| Q4437_23050 | methyl-accepting chemotaxis protein | -1.525873982 | 0.001615132 | 0.016699787 |
| Q4437_23055 | acetate/propionate family kinase | -1.538211555 | 0.000537284 | 0.007089557 |
| Q4437_23090 | MaoC family dehydratase | -2.475777971 | 8.26E-08 | 5.98E-06 |
| Q4437_23150 | polymer-forming cytoskeletal protein | -2.489569827 | 5.36E-05 | 0.001101082 |
| Q4437_23165 | hypothetical protein | 1.340316842 | 0.00203738 | 0.020033482 |
| Q4437_23195 | DUF3820 family protein | -3.2585659 | 0.000739506 | 0.009145437 |
| Q4437_23210 | choice-of-anchor I family protein | -4.538222731 | 1.58E-05 | 0.000413997 |
| Q4437_23240 | cation diffusion facilitator family transporter | -1.27664002 | 0.002519872 | 0.023312522 |
| Q4437_23270 | isoprenoid biosynthesis glyoxalase ElbB | 1.477213775 | 0.000686338 | 0.008633002 |
| Q4437_23290 | Dps family protein | -1.352500229 | 0.002497303 | 0.023246065 |
| Q4437_23375 | heme o synthase | -3.008086189 | 0.000419448 | 0.005878934 |
| Q4437_23390 | SURF1 family protein | -1.693121771 | 0.00171636 | 0.01762263 |
| Q4437_23400 | cytochrome c oxidase subunit 3 | -2.199284367 | 0.000339042 | 0.004923917 |
| Q4437_23405 | cytochrome c oxidase assembly protein | -2.245448055 | 0.001436097 | 0.01532608 |
| Q4437_23410 | cytochrome c oxidase subunit I | -2.340524954 | 5.74E-05 | 0.00114389 |
| Q4437_23430 | LysE family translocator | -2.54656874 | 0.000883969 | 0.010440557 |
| Q4437_23455 | porin | 2.285592272 | 1.83E-07 | 1.01E-05 |
| Q4437_23460 | porin | -5.234842952 | 2.38E-18 | 1.31E-15 |
| Q4437_23490 | LysR family transcriptional regulator | -2.086356267 | 0.000759269 | 0.009337534 |
| Q4437_23535 | aromatic amino acid transport family protein | -2.069616962 | 1.03E-05 | 0.000308041 |
| Q4437_23545 | helix-turn-helix transcriptional regulator | -6.983382137 | 0.000193974 | 0.003125533 |
| Q4437_23570 | zinc-binding dehydrogenase | -2.898880874 | 2.71E-08 | 2.34E-06 |
| Q4437_23575 | PTS mannitol transporter subunit IICB | -3.746370397 | 1.36E-11 | 2.31E-09 |
| Q4437_23580 | PTS sugar transporter subunit IIA | -8.064796157 | 1.67E-07 | 9.58E-06 |
| Q4437_23605 | YbhB/YbcL family Raf kinase inhibitor-like protein | -1.745903978 | 0.001078269 | 0.012368251 |
| Q4437_23645 | endonuclease/exonuclease/phosphatase family protein | -3.050057652 | 2.42E-05 | 0.000587876 |
| Q4437_23650 | formate dehydrogenase subunit alpha | -1.373657477 | 0.001163594 | 0.013105274 |
| Q4437_23655 | hypothetical protein | -3.076426154 | 5.61E-08 | 4.43E-06 |
| Q4437_23660 | TolC family protein | -7.303028101 | 3.05E-05 | 0.000707781 |
| Q4437_23670 | CusA/CzcA family heavy metal efflux RND transporter | -2.160876373 | 0.002409866 | 0.022775908 |
| Q4437_23715 | efflux RND transporter permease subunit VmeQ | -2.245448055 | 0.001436097 | 0.01532608 |
| Q4437_23735 | ATP-binding cassette domain-containing protein | -2.349067263 | 7.00E-06 | 0.000222283 |
| Q4437_23755 | hypothetical protein | 3.151037272 | 7.29E-11 | 1.07E-08 |
| Q4437_23770 | PAS factor family protein | 1.438149176 | 0.000731806 | 0.009092875 |
| -- | hypothetical protein | 1.572855262 | 0.000931201 | 0.010905175 |
| -- | hypothetical protein | 2.53654271 | 4.79E-06 | 0.00015917 |
| -- | hypothetical protein | 1.185441985 | 0.005084403 | 0.038306552 |
| -- | hypothetical protein | -1.869763023 | 8.57E-05 | 0.00162455 |
| -- | hypothetical protein | -1.75402871 | 5.74E-05 | 0.00114389 |
| -- | hypothetical protein | -2.281431141 | 0.000415 | 0.005853748 |
| -- | hypothetical protein | 1.425685582 | 0.000793118 | 0.009609734 |
| -- | hypothetical protein | 1.34207997 | 0.006611351 | 0.046852512 |
| -- | hypothetical protein | -8.064796157 | 1.67E-07 | 9.58E-06 |
| -- | hypothetical protein | 2.359832924 | 1.38E-07 | 8.49E-06 |
| -- | hypothetical protein | -1.546016063 | 0.000414807 | 0.005853748 |
| -- | hypothetical protein | -1.707613173 | 9.87E-05 | 0.001823439 |
| -- | hypothetical protein | -8.730223875 | 2.96E-10 | 3.53E-08 |
| -- | hypothetical protein | -2.95575771 | 0.003515158 | 0.030018222 |
| -- | hypothetical protein | -2.431313179 | 0.003149424 | 0.027753908 |
| -- | hypothetical protein | 4.677198314 | 1.69E-15 | 6.77E-13 |
| -- | hypothetical protein | 1.696559328 | 9.12E-05 | 0.001712526 |
| -- | hypothetical protein | 2.174793322 | 2.52E-06 | 9.13E-05 |
| -- | hypothetical protein | 2.317173646 | 1.61E-07 | 9.58E-06 |
| -- | hypothetical protein | -2.761476739 | 0.000211448 | 0.003362204 |
| -- | hypothetical protein | 1.756653082 | 0.000392038 | 0.005637939 |
| -- | hypothetical protein | -1.264352367 | 0.004576868 | 0.035575477 |
|  |  |  |  |  |
