# Supplementary material for: The two-component system TtrRS boosts Vibrio parahaemolyticus colonization by exploiting sulfur compounds in host gut
Source: PLoS Pathog. 2024 Jul 22;20(7):e1012410. doi: 10.1371/journal.ppat.1012410 (PMC11293645; doi:10.1371/journal.ppat.1012410)
Supplement: S2 Table — (DOCX) [file ppat.1012410.s010.docx]

**Table S2** **Bacterial strains and plasmids used in this study**

| **Strains or plasmids** | **Description** | **Source** |
| --- | --- | --- |
| **Bacterial strains** |  |  |
| HZ | *V. parahaemolyticus*, WT | Our laboratory |
| ∆*01695* | Deletion mutant of *01695* with WT background | This study |
| ∆*02355* | Deletion mutant of *02355* with WT background | This study |
| ∆*02575* | Deletion mutant of *02575* with WT background | This study |
| ∆*02755* | Deletion mutant of *02755* with WT background | This study |
| ∆*04505* | Deletion mutant of *04505* with WT background | This study |
| ∆*05090* | Deletion mutant of *05090* with WT background | This study |
| ∆*05910* | Deletion mutant of *05910* with WT background | This study |
| ∆*07555* | Deletion mutant of *07555* with WT background | This study |
| ∆*08335* | Deletion mutant of *08335* with WT background | This study |
| ∆*08450* | Deletion mutant of *08450* with WT background | This study |
| ∆*09700* | Deletion mutant of *09700* with WT background | This study |
| ∆*09830* (∆*ttrR*) | Deletion mutant of *09830* with WT background | This study |
| ∆*10675* | Deletion mutant of *10675* with WT background | This study |
| ∆*11005* | Deletion mutant of *11005* with WT background | This study |
| ∆*12120* | Deletion mutant of *12120* with WT background | This study |
| ∆*17525* | Deletion mutant of *17525* with WT background | This study |
| ∆*16890* | Deletion mutant of *16890* with WT background | This study |
| ∆*16725* | Deletion mutant of *16725* with WT background | This study |
| ∆*22465* | Deletion mutant of *22465* with WT background | This study |
| ∆*22435* | Deletion mutant of *22435* with WT background | This study |
| ∆*22345* | Deletion mutant of *22345* with WT background | This study |
| ∆*21590* | Deletion mutant of *21590* with WT background | This study |
| ∆*21550* | Deletion mutant of *21550* with WT background | This study |
| ∆*20835* | Deletion mutant of *20835* with WT background | This study |
| ∆*19875* | Deletion mutant of *19875* with WT background | This study |
| ∆*19135* | Deletion mutant of *19135* with WT background | This study |
| ∆*18635* | Deletion mutant of *18635* with WT background | This study |
| ∆*17710* | Deletion mutant of *17710* with WT background | This study |
| ∆*09850* (∆*ttrA*) | Deletion mutant of *09850* with WT background | This study |
| C∆*09850* (C∆*ttrA*) | ∆*09850* (∆*ttrA*) with the plasmid pBAD24-*ttrA* | This study |
| C∆*ttrR*^WT^ | Complement of *ttrR* with ∆*ttrR* background | This study |
| C∆*ttrR* | ∆*ttrR* with the plasmid pBAD24-*ttrR* | This study |
| C∆*ttrR*^D58A^ | Asp58 of TtrR was changed to alanine on WT background | This study |
| ∆*ttrS* | Deletion mutant of *09835* with WT background | This study |
| C∆*ttrS*^WT^ | Complement of *ttrS* with ∆*ttrS* background | This study |
| C∆*ttrS*^H397A^ | His397 of TtrS was changed to alanine on WT background | This study |
| ∆*09855-09860* (∆*tsdBA*) | Deletion mutant of *09855-09860* with WT background | This study |
| C∆*09855-09860* (C∆*tsdBA*) | ∆*09855-09860* with the plasmid pBAD24-*tsdB-tsdA* | This study |
| ∆*05910* | Deletion mutant of 05910 with WT background | This study |
| ∆*ttrR*∆*05910* | Deletion mutant of *09830* and 05910 with WT background | This study |
| WT::P*_ttrB_*_-Δbox_ | The *ttrR* box was deleted from the *ttrBCA* promoter on WT background | This study |
| WT::P*_tsdB_*_-Δbox_ | The *ttrR* box was deleted from the *tsdBA* promoter on WT background | This study |
| Δ*ttrR*::P*_ttrB-cm_* | *ttrBCA* promoter was changed to a constitutively expressed promoter P*_ttrB-cm_* on Δ*ttrR* background | This study |
| Δ*ttrR*::P*_tsdB-km_* | *tsdBA* promoter was changed to a constitutively expressed promoter P*_tsdB-km_* on Δ*ttrR* background | This study |
| Δ*ttrR*::P*_ttrB-cm_* & P*_tsdB-km_* | Both *ttrBCA* or *tsdBA* promoters were changed on Δ*ttrR* background | This study |
| *E. coli* DH5a | Cloning host for maintaining the recombinant plasmids | Our laboratory |
| *E. coli* CC118λpir | Mobilization of plasmids into *V. parahaemolyticus* | Our laboratory |
| *E. coli* BTH101 | Two-hybrid system detected strain | Our laboratory |
| *E. coli* BL21 (DE3) | Host for expressing the recombinant proteins | Our laboratory |
| RIMD 2210633 | A pandemic *V. parahaemolyticus* strain | Our laboratory |
| Δ*ttrR* (Δ*vp2009*) | Deletion mutant of *vp2009* with RIMD 2210633 background | This study |
| Δ*ttrA* (Δ*vp2014*) | Deletion mutant of *vp2014* with RIMD 2210633 background | This study |
| Δ*tsdBA* (Δ*vp2015-2016*) | Deletion mutant of *vp2015-2016* with RIMD 2210633 background | This study |
| **Plasmids** |  |  |
| pDS132 | Suicide vector for *V. parahaemolyticus* mutagenesis | Our laboratory |
| pDS132-*01695* | Derived from pDS132 used to knock out *01695* | This study |
| pDS132-*02355* | Derived from pDS132 used to knock out *02355* | This study |
| pDS132-*02575* | Derived from pDS132 used to knock out *02575* | This study |
| pDS132-*02755* | Derived from pDS132 used to knock out *02755* | This study |
| pDS132-*04505* | Derived from pDS132 used to knock out *04505* | This study |
| pDS132-*05090* | Derived from pDS132 used to knock out *05090* | This study |
| pDS132-*05910* | Derived from pDS132 used to knock out *05910* | This study |
| pDS132-*07555* | Derived from pDS132 used to knock out *07555* | This study |
| pDS132-*08335* | Derived from pDS132 used to knock out *08335* | This study |
| pDS132-*08450* | Derived from pDS132 used to knock out *08450* | This study |
| pDS132-*09700* | Derived from pDS132 used to knock out *09700* | This study |
| pDS132-*09830* (*ttrR*) (*vp2009*) | Derived from pDS132 used to knock out *09830* (*ttrR*) (*vp2009*) | This study |
| pDS132-*10675* | Derived from pDS132 used to knock out *10675* | This study |
| pDS132-*11005* | Derived from pDS132 used to knock out *11005* | This study |
| pDS132-*12120* | Derived from pDS132 used to knock out *12120* | This study |
| pDS132-*17525* | Derived from pDS132 used to knock out *17525* | This study |
| pDS132-*16890* | Derived from pDS132 used to knock out *16890* | This study |
| pDS132-*16725* | Derived from pDS132 used to knock out *16725* | This study |
| pDS132-*22465* | Derived from pDS132 used to knock out *22465* | This study |
| pDS132-*22435* | Derived from pDS132 used to knock out *22435* | This study |
| pDS132-*22345* | Derived from pDS132 used to knock out *22345* | This study |
| pDS132-*21590* | Derived from pDS132 used to knock out *21590* | This study |
| pDS132-*21550* | Derived from pDS132 used to knock out *21550* | This study |
| pDS132-*20835* | Derived from pDS132 used to knock out *20835* | This study |
| pDS132-*19875* | Derived from pDS132 used to knock out *19875* | This study |
| pDS132-*19135* | Derived from pDS132 used to knock out *19135* | This study |
| pDS132-*18635* | Derived from pDS132 used to knock out *18635* | This study |
| pDS132-*17710* | Derived from pDS132 used to knock out *17710* | This study |
| pDS132-*09850* (*ttrA*) (*vp2014*) | Derived from pDS132 used to knock out *09850* (*ttrA*) (*vp2014*) | This study |
| pDS132-*ttrR*^WT^ | Derived from pDS132 for the complement of *ttrR* with ∆*ttrR* background | This study |
| pDS132-*ttrR*^D58A^ | Derived from pDS132 used to make the *ttrR*^D58A^ variant | This study |
| pDS132-*ttrS* | Derived from pDS132 used to knock out *09835* | This study |
| pDS132-*ttrS*^WT^ | Derived from pDS132 for the complement of *ttrS* with ∆*ttrS* background | This study |
| pDS132-*ttrS*^H397A^ | Derived from pDS132 used to make the *ttrS*^H397A^ variant | This study |
| pDS132-*09855-09860* (*tsdBA*) (*vp2015-2016*) | Derived from pDS132 used to knock out *09855-09860* (*tsdBA*) (*vp2015-2016*) | This study |
| pDS132-*05910* | Derived from pDS132 used to knock out *05910* | This study |
| pDS132-P*_ttrB_*_-Δbox_ | Derived from pDS132 used to knock out *ttrR* box on *ttrBCA* promoter | This study |
| pDS132-P*_tsdB_*_-Δbox_ | Derived from pDS132 used to knock out *ttrR* box on *tsdBA* promoter | This study |
| pDS132-P*_ttrB-cm_* | Derived from pDS132 used to replace *ttrBCA* promoter with P*_ttrB-cm_* | This study |
| pDS132-P*_tsdB-km_* | Derived from pDS132 used to replace *tsdBA* promoter with P*_tsdB-km_* | This study |
| pBAD24 | Arabinose-induced expression plasmid | Our laboratory |
| pBAD24-*ttrA* | Derived from pBAD24 used to overexpress TtrA protein | This study |
| pBAD24-*ttrS*-*ttrR* | Derived from pBAD24 used to overexpress TtrS and TtrR proteins | This study |
| pBAD24-*ttrR* | Derived from pBAD24 used to overexpress TtrR protein | This study |
| pBAD24-*ttrR*^D58E^ | Derived from pBAD24-*ttrR* used to overexpress TtrR^D58E^ variant protein | This study |
| pBAD24-*ttrS*^H397A^*-ttrR* | Derived from pBAD24-ttrS-ttrR to overexpress TtrR and TtrS^H397A^ variant proteins | This study |
| pBAD24-*tsdB-tsdA* | Derived from pBAD24 used to overexpress TsdB and TsdA proteins | This study |
| pKNT25 | The plasmid expressing the T25 fusion used for two-hybrid system | Our laboratory |
| pUT18 | The plasmid expressing the T18 fusion used for two-hybrid system | Our laboratory |
| pKNT25-*ttrR*-*ttrS* | Derived from pKNT25 used for two-hybrid system | This study |
| pUT18-*ttrR*-*ttrS* | Derived from pUT18 used for two-hybrid system | This study |
| pKNT25-*ttrR*-*ttrS*^ΔN^ | Derived from pKNT25 used for two-hybrid system, the N-terminal domain of TtrS was deleted | This study |
| pUT18-*ttrR*-*ttrS*^ΔN^ | Derived from pUT18 used for two-hybrid system, the N-terminal domain of TtrS was deleted | This study |
| pKNT25-*ttrR*^WT^ | Derived from pKNT25 used for two-hybrid system | This study |
| pUT18-*ttrR*^WT^ | Derived from pUT18 used for two-hybrid system | This study |
| pKNT25-*ttrR*^D58E^ | Derived from pKNT25 used for two-hybrid system | This study |
| pUT18-*ttrR*^D58E^ | Derived from pUT18 used for two-hybrid system | This study |
| pKNT25-*ttrR*-*ttrS* ^H397A^ | Derived from pKNT25 used for two-hybrid system | This study |
| pUT18-*ttrR*-*ttrS* ^H397A^ | Derived from pUT18 used for two-hybrid system | This study |
| pBBR-*lux* | The bioluminescence reporter containing *luxCDABE* operon of *Vibrio harveyi* | Our laboratory |
| P*_ttrS_*-*lux* | pBBR-lux containing the promoter of *ttrS* | This study |
| P*_ttrB_*-*lux* | pBBR-lux containing the promoter of *ttrB* | This study |
| P*_ttrB-_*_Δbox_-*lux* | pBBR-lux containing the promoter of *ttrB* with *ttrR* box deleted | This study |
| P*_09855_* -*lux* (P*_tsdB_*-*lux*) | pBBR-lux containing the promoter of *09855* | This study |
| P*_tsdB-_*_Δbox_-*lux* | pBBR-lux containing the promoter of *09855* with *ttrR* box deleted | This study |
| P*_05905_-lux* | pBBR-lux containing the promoter of *05905* | This study |
