# Supplementary material for: The two-component system TtrRS boosts Vibrio parahaemolyticus colonization by exploiting sulfur compounds in host gut
Source: PLoS Pathog. 2024 Jul 22;20(7):e1012410. doi: 10.1371/journal.ppat.1012410 (PMC11293645; doi:10.1371/journal.ppat.1012410)
Supplement: S3 Table — (DOCX) [file ppat.1012410.s011.docx]

**Table S3 Primers used in this study**

| **Primer** | **Primer sequence (5'–3')** |
| --- | --- |
| pDS132-*01695* | P1: GCTCTAGAAAGCTAAAATCAAACGTTGAAGAAGTGCGT |
|  | P2: GTGAATCAGTGGCAAACCTTTTTGCAGATA |
|  | P3: CAAAAAGGTTTGCCACTGATTCACACTGTC |
|  | P4: ACATGCATGCCGGTCAATTTCCTCCTGCACCGCAAACACT |
| pDS132-*02355* | P1: CGAGCTCTGTTGATCGAGTCGATAAACCCGCGGTGAT |
|  | P2: CTTATCGTAGAATACCGCTTCTGTGGTGAC |
|  | P3: ACAGAAGCGGTATTCTACGATAAGAATCTGCGG |
|  | P4: GCTCTAGATAGAATTATCGTGATAGCAACGCACTGTGA |
| pDS132-*02575* | P1: GCTCTAGAATGTTCGCCATGAAGTGGTACATAACCGTT |
|  | P2: CTGGCAGAGCTCATTCCAGTAAGCCGTCGT |
|  | P3: GCTTACTGGAATGAGCTCTGCCAGCTCTTC |
|  | P4: ACATGCATGCGCATTGGCGATGACCACCATAGACCGAATA |
| pDS132-*02755* | P1: ACATGCATGCGCATGCCAGTTAAGCGCAAGTTTCGTCACGACTTTG |
|  | P2: CAGTTTGTCGTGGAAGCAAAGCATTTCACG |
|  | P3: ATGCTTTGCTTCCACGACAAACTGATTCAAA |
|  | P4: AACTGCAGAACTTGAGTTTATGTTGGTCATCACCACTT |
| pDS132-*04505* | P1: GCTCTAGAGTGTACTCATTCTTGATCGCGAACTTTATA |
|  | P2: ATAGAACAAACGCGAAAAACGCTGTATGAC |
|  | P3: CAGCGTTTTTCGCGTTTGTTCTATTGCAAATCG |
|  | P4: ACATGCATGCATATCGTTGTGCTTACGTTGGCCTTTACGG |
| pDS132-*05090* | P1: GCTCTAGACTAAAAATAGGCGGTGATTATTCACCAAGA |
|  | P2: TTGTGGGTTCTTTCCCGCAAGCTTACTACG |
|  | P3: AAGCTTGCGGGAAAGAACCCACAAATTTTTGT |
|  | P4: CGAGCTCGCACAAGGAAAGCAGGAATAAAGAGGGTCACCCTCATTTTTATTATCAATTGATGTTTTT |
| pDS132-*05910* | P1: GCGGAGCTCTTGCTGCCAACAATGTGGGT |
|  | P2: TTTATGAATCAAAACAGGCACTTCATCGTCAAC |
|  | P3: TGAAGTGCCTGTTTTGATTCATAAAGTAGGGCA |
|  | P4: GCGGAGCTCGTCCGCACCTTAATTAGAA |
| pDS132-*07555* | P1: CGAGCTCCTATGGCTATCTATGACTCAGGCCCACATG |
|  | P2: TATTAATGATTGTATAGCTTGGTATGTGTCTAAATATTTATCTTT |
|  | P3: TATTTAGACACATACCAAGCTATACAATCATTAATAGAAACGG |
|  | P4: ACATGCATGCCTCATCGAAACTTTTAAGGATGTTAGTTGC |
| pDS132-*08335* | P1: GCTCTAGATGGTCTAAACGTGTTGCCAGCTTGCTGTAA |
|  | P2: ACGCGAGTGATGATCATTGAACGTTGTTATAAAAAAGTGATA |
|  | P3: TTTTTTATAACAACGTTCAATGATCATCACTCGCGT |
|  | P4: CGAGCTCGAGTACTATCAAAGAAGGTGTACTCAGCAT |
| pDS132-*08450* | P1: GCTCTAGAGCTCGACAGAGAGCTGTTCGCAACCGGAAT |
|  | P2: AATGTACCCCACACCACGTTTGTCATCTTCTACGAGTAA |
|  | P3: CTCGTAGAAGATGACAAACGTGGTGTGGGGTAC |
|  | P4: ACATGCATGCAAGACGCGTTCAGATAAACTGTCTACTTTA |
| pDS132-*09700* | P1: GCTCTAGAGGCTTTTGTCTTGAATCGTTGGCTCAACCC |
|  | P2: CTCCAAGCGGAAGCCGACATTATCTTCTACCAAAAGTACACG |
|  | P3:CTTTTGGTAGAAGATAATGTCGGCTTCCGCTTG |
|  | P4: CGAGCTCTAAATTCTAACGACTTTTCATCGGCTTGCG |
| pDS132-*09830* (*ttrR*) (*vp2009*) | P1: GCGTCTAGATTGTGATAACCTTAAACCCA |
|  | P2: AAAAGCAGCTGTACATTTATGGCAAGTTGGA |
|  | P3: CCATAAATGTACAGCTGCTTTTCACTGCTGA |
|  | P4: GCGTCTAGAGTCAGGCTGGACATCGCCAG |
| pDS132-*10675* | P1: GCTCTAGATAATACCGATTTACTTAGATTGGCTCATCA |
|  | P2: CACAGTCAAACGGCAATGAATATTACTCGAGTTAATAGACTT |
|  | P3: ATTAACTCGAGTAATATTCATTGCCGTTTGACTGT |
|  | P4: ACATGCATGCTCTGAACAAGCACTGCGAACAACATTGAGT |
| pDS132-*11005* | P1: CGAGCTCGGCTGAGCTTAAGACGTTCCTGCTCTAGGC |
|  | P2: AGCCCTCGTGCGTACTCTGCGCTACAAACTTGCCA |
|  | P3: GCAGAGTACGCACGAGGGCTTCACGAAGACCTTCG |
|  | P4: AACTGCAGAACCCCGAGGCACACCGAATTCTAGAAATC |
| pDS132-*12120* | P1: GCTCTAGATTGTGGTTGTCGGTGAAAACCGTTCCGGCG |
|  | P2: CAGTCTTTGCAATTTATCAAATTTCTGTTTTTTGTAGAATTTAAA |
|  | P3: TACAAAAAACAGAAATTTGATAAATTGCAAAGACTGCTC |
|  | P4: ACATGCATGCTTTTTTTAATATTGGCAAGCATAGCTGTTC |
| pDS132-*17525* | P1: GCTCTAGAGCGGTCCGTACTACGTGCATGTGCTGGTGA |
|  | P2: AGTTTCGCCAACGAGATGATGCCGCAATAA |
|  | P3: CGGCATCATCTCGTTGGCGAAACTAAGTTG |
|  | P4: CGAGCTCAGCGGGCATGGATGTGGTTAATCGCATCGC |
| pDS132-*16890* | P1: GCTCTAGACTCAGATGATTACCTCGATTTGTTAGGTGC |
|  | P2: TTTGATGTGCTGAACTACTTCTAAAAGCTC |
|  | P3: TTAGAAGTAGTTCAGCACATCAAAACGTTT |
|  | P4: CGAGCTCCTTCAAGATCGCTACGCTTGGTCTCTTTGT |
| pDS132-*16725* | P1: GCTCTAGATCTACTGCATGGTTTGGCTCATTGAACCAG |
|  | P2: CTGATTTAGGGTCTGACGAACAATGTCATCA |
|  | P3: ATTGTTCGTCAGACCCTAAATCAGAAAATGCA |
|  | P4: ACATGCATGCGTATTCAGTGGCCTTTTTTATTACTTCACA |
| pDS132-*22465* | P1: GCTCTAGATGCCACTCAGCATTCGTCAGTGGTGTGACG |
|  | P2: TTTGTCGATGTAAAGTATTGTGGTTTGTAGTGG |
|  | P3: ACCACAATACTTTACATCGACAAACCCATG |
|  | P4: CGAGCTCAAATACGCTTCGGCTTGATGCAGCGATTTG |
| pDS132-*22435* | P1: ACATGCATGCGCATGCGTGCAAATGAAGCAAAGTCATCGGCATGAT |
|  | P2: AGACTGGTTACCATTAAATACAACAACATG |
|  | P3: GTTGTATTTAATGGTAACCAGTCTGGAATC |
|  | P4: AACTGCAGTAAAGAACTTCTTCCTACGGTAAAAAATCC |
| pDS132-*22345* | P1: GCTCTAGATTAAAAACGCATCAAGGCGT |
|  | P2: GACTAACTTATTGCGATAATCATCTTCCACTATGACTAC |
|  | P3: GTCATAGTGGAAGATGATTATCGCAATAAGTTAGTCTCA |
|  | P4: CGAGCTCCTCGGCGGTAAATCGTTTAT |
| pDS132-*21590* | P1: GCTCTAGACTTACCGCTAAAACATATCTATCGTTTGGT |
|  | P2: CTGCTTCTCGTTGAGGACCTGAGTTTGATTTGCTTCTGTT |
|  | P3: GAAGCAAATCAAACTCAGGTCCTCAACGAGAAGCA |
|  | P4: CGAGCTCCGTGACAACGAAGAAGGTGACGAGTTACTA |
| pDS132-*21550* | P1: CGAGCTCCCATTTACCCAATACCAACCTTCCGGCTTT |
|  | P2: TGGCTATTCGTCAGTTTGTGGTTTTACTCAGTA |
|  | P3: AAACCACAAACTGACGAATAGCCAACTGCA |
|  | P4: ACATGCATGCATTGCCTGCCGAAGTTCATTCGATAGAAGC |
| pDS132-*20835* | P1: CGAGCTCGAAGCTTGCTGGCGCATACACTTCGCCGCT |
|  | P2: ATTGTTGATGATAACCACCATGGACAAGGCTACCG |
|  | P3: TCGGTAGCCTTGTCCATGGTGGTTATCATCAACAATTAAAAT |
|  | P4: GCTCTAGACATTTCTCAAGACAGAAGAAACGTCGACTT |
| pDS132-*19875* | P1: ACATGCATGCTATTTCCTTAGTATGAGTTCATCG |
|  | P2: GTTTAAACACAACACGCCGTAAGTGATGTGTGCTGT |
|  | P3: ACAGCACACATCACTTACGGCGTGTTGTGTTTA |
|  | P4: AACTGCAGTGGGCTTCTTTGCAATCCGTATCTTT |
| pDS132-*19135* | P1: GCTCTAGACTGTGGTGGCAATCGGCAGTTTGAGTAAGA |
|  | P2: GTTTTAGTCGTGGACGATTTAGTGCTTGAGACGTCC |
|  | P3: GGACGTCTCAAGCACTAAATCGTCCACGACTAAAAC |
|  | P4: CGAGCTCATGGCTGTTAGACCAACTGTTTGACTCTAC |
| pDS132-*18635* | P1: GCTCTAGATGAGCTGTCTTCTTTCAGCG |
|  | P2: ACGAATATTTTGTTGGTTCAAAGTCCAGCTTTGGT |
|  | P3: GACCAAAGCTGGACTTTGAACCAACAAAATATTCGTTTTCG |
|  | P4: CGAGCTCATGGAAATGTAAGATATACTTTT |
| pDS132-*17710* | P1: GCTCTAGAATGTAGTCTATTTGATTTTATACGCGCTGA |
|  | P2: AAACTTCGATTTGTCCAAGTCATCAACAAAGATAACTGC |
|  | P3: GTTATCTTTGTTGATGACTTGGACAAATCGAAGTTTAAG |
|  | P4: CGAGCTCACTGGGATTTTCAGTTCGCTAGATGTCATT |
| pDS132-*09850* (*ttrA*) (*vp2014*) | P1: GCTCTAGACTGGCGACCTTCACCAGCCGGGACGAGC |
|  | P2: CCACCCAATCTGCGCCAAGCCCACCGAC |
|  | P3: GCTTGGCGCAGATTGGGTGGTAGGAGCC |
|  | P4: CGAGCTCTTTGGAATTTTACTCAGATCGCGAGGCT |
| pDS132-*ttrR*^WT^ | P1:CGGAATTCATGATCAGCAGTGAAAAGCAGCTGCCTGTT |
|  | P2:AACTGCAGCTAGATGGGATTCTCCAACTTGCCATAAAT |
| pDS132-*ttrR*^D58A^ | P1:CGGAATTCATGATCAGCAGTGAAAAGCAGCTGCCTGTT |
|  | P2:TCCTTGCCAGCCGAATGCCTAACCTTCGTG |
|  | P3:AGGCATTCGGCTGGCAAGGATCACGCATCC |
|  | P4:AACTGCAGCTAGATGGGATTCTCCAACTTGCCATAAAT |
| pDS132-*ttrS* | P1: GCGTCTAGAACCGAAACGACTGCGAACTT |
|  | P2: CCGCAAAACTCGAACGCCATTGATGCGTGTGCTT |
|  | P3: ATCAATGGCGTTCGAGTTTTGCGGTGCGCGCCCC |
|  | P4: GCGTCTAGACTACGCAGCGACTGTTATCC |
| pDS132-*ttrS*^WT^ | P1: GCGTCTAGAACCGAAACGACTGCGAACTT |
|  | P2: GCGTCTAGACTACGCAGCGACTGTTATCC |
| pDS132-*ttrS*^H397A^ | P1:GCGTCTAGAACCGAAACGACTGCGAACTT |
|  | P2:CTGGCTGCCGAATTAAATCAACCGCTGGCAGCTA |
|  | P3:TGATTTAATTCGGCAGCCAGACTGCTGCCA |
|  | P4:GCGTCTAGACTACGCAGCGACTGTTATCC |
| pDS132-*09855-09860* (*tsdBA*) (*vp2015-2016*) | P1: GCTCTAGAAGGCGGCGTGCCCGAAGTTTCGGCAGAAGA |
|  | P2: GTTTAGTGTTTTGCTTGCGCCCATTGTGGT |
|  | P3: ATGGGCGCAAGCAAAACACTAAACGGTAAG |
|  | P4: CGAGCTCCACAGCCAGCTTTTATCGACCATCGAAATG |
| pDS132-*05910* | P1: GCGGAGCTCTTGCTGCCAACAATGTGGGT |
|  | P2: TTTATGAATCAAAACAGGCACTTCATCGTCAAC |
|  | P3: TGAAGTGCCTGTTTTGATTCATAAAGTAGGGCA |
|  | P4: GCGGAGCTCGTCCGCACCTTAATTAGAA |
| pDS132-P*_ttrB_*_-Δbox_ | P1: GCTCTAGAACGAGCCGCGTTCTGTTTTGGCAAACGACC |
|  | P2: GACGTCGATATAATACCCAGATCAAAGTTGATC |
|  | P3: TGATCTGGGTATTATATCGACGTCGCAGCG |
|  | P4: CGAGCTCTACTGCACCGCCCACGGGAGCCAAGCCATC |
| pDS132-P*_tsdB_*_-Δbox_ | P1: GCTCTAGATGCGCAAGGGAATTGGCACGCTCTCCACAG |
|  | P2: ACTAAAGGTATTATATTTAGAGGATGTGAAGAGCTGCTAAAT |
|  | P3: TCCTCTAAATATAATACCTTTAGTTATTGACAAAGC |
|  | P4: CGAGCTCCACGTTTTACTTGATCACCAAATGCGGTGT |
| pDS132-P*_ttrB-cm_* | P1: GCTCTAGATTTGGATATAACGGCGTAGAAACGGCACAC |
|  | P2: GAGAAAGCGGCCGCTTTTTTCCTTCGACGCGTTGCGCTGCGACGTCGATAT |
|  | P3: TCGACGTCGCAGCGCAACGCGTCGAAGGAAAAAAGCGGCCGCTTTCTCACAATGGAATCAGTC |
|  | P4: CGAGCTCCAGAACAGATGTAAAAAAAGTACTGCACCG |
|  | Pcm-Fwd-MluI: GCGACGCGTCGCACATATTCTGCTGACGCAC |
|  | Pcm-Rev-NotI: CGATAAGAATGCGGCCGCTAAAGCTTCCTTAGCTCCTGAAAATCTCG |
| pDS132-P*_tsdB-km_* | P1: GCTCTAGAAGGCGGCGTGCCCGAAGTTTCGGCAGAAGA |
|  | P2: ATATCTGCGGCCGCTTTTTTCCTTCGACGCGTCCAAGTGTCTACTGTGATA |
|  | P3: CACAGTAGACACTTGGACGCGTCGAAGGAAAAAAGCGGCCGCAGATATTAATAACAATTAAAGTGAGTC |
|  | P4: CGAGCTCCACAGTTTTGCTCATTGCCAACGTATTTAC |
|  | Pkm-Fwd-MluI: CGCGACGCGTCGTGAGGTCTGCCTCGTGAAGAAGGTGTTGCT |
|  | Pkm-Rev-NotI: CGATAAGAATGCGGCCGCTAAACCGGGTACCATGGAACACCCCTTGTATTACTGT |
| pBAD24-*ttrA* | P1:GGGGTACCGATGGATAACAAACGCCGTCAGTTTTTGAAA |
|  | P2:ACATGCATGCCTAAGCTTTGCGGATTTTGGCTGGTAATGA |
| pBAD24-*ttrS*-*ttrR* | P1:GCGGGTACCGCAGCAGCAAAAGACGGTGCAG |
|  | P2:GCGTCTAGACTAGATGGGATTCTCCAACT |
| pBAD24-*ttrR* | P1:GCGGGTACCGATCAGCAGTGAAAAGCAG |
|  | P2: GCGAAGCTTCTAGATGGGATTCTCCAACT |
| pBAD24-*ttrR*^D58E^ | P1:GCGGGTACCGATCAGCAGTGAAAAGCAG |
|  | P2: AGGCATTCGGCTTTCAAGGATCACGCATCCGG |
|  | P3: CGTGATCCTTGAAAGCCGAATGCCTAACCTTCG |
|  | P4: GCGAAGCTTCTAGATGGGATTCTCCAACT |
| pBAD24-*ttrS*^H397A^*-ttrR* | P1: CGGAATTCATGCAGCAGCAAAAGACGGTGCAGCCAGAG |
|  | P2:CTGGCTGCCGAATTAAATCAACCGCTGGCAGCTA |
|  | P3:TGATTTAATTCGGCAGCCAGACTGCTGCCA |
|  | P4: AACTGCAGCTAGATGGGATTCTCCAACTTGCCATAAAT |
| pBAD24-*tsdB-tsdA* | P1: GGGGTACCGATGTCAAAAATACTACCTGTGGTGACGGCC |
|  | P2: ACATGCATGCCTACTCAACCGTTCCGCTTGCAAATGCGTT |
| pKNT25-*ttrR*-*ttrS* | P1:CGGAATTCATGGGATTCTCCAACTTGCCATAAATGTAG |
|  | P2: CGGGATCCCATGAACAAAACGCCACGGTTGCTTCAGTT |
| pUT18-*ttrR*-*ttrS* | P1:CGGAATTCATGGGATTCTCCAACTTGCCATAAATGTAG |
|  | P2: CGGGATCCCATGAACAAAACGCCACGGTTGCTTCAGTT |
| pKNT25-*ttrR*-*ttrS*^ΔN^ | P1: CAGGTCGACTCTAGAGGATCCACCAGAGCACCCTGCCATTA |
|  | P2: TGCTGCATGGTCATTGAATTCGAGATGGGATTCTCCAACTTGCC |
| pUT18-*ttrR*-*ttrS*^ΔN^ | P1: CAGGTCGACTCTAGAGGATCCACCAGAGCACCCTGCCATTA |
|  | P2: GCCTCGCTGGCGGCTGAATTCGAGATGGGATTCTCCAACTTGCC |
| pKNT25-*ttrR*^WT^ | P1: GCGGGATCCAATGATCAGCAGTGAAAAGC |
|  | P2: GCGGGTACCCTAGATGGGATTCTCCAACT |
| pUT18-*ttrR*^WT^ | P1: GCGGGATCCAATGATCAGCAGTGAAAAGC |
|  | P2: GCGGGTACCCTAGATGGGATTCTCCAACT |
| pKNT25-*ttrR*^D58E^ | P1: GCGGGATCCAATGATCAGCAGTGAAAAGC |
|  | P2: AGGCATTCGGCTTTCAAGGATCACGCATCCGG |
|  | P3: CGTGATCCTTGAAAGCCGAATGCCTAACCTTCG |
|  | P4: GCGGGTACCCTAGATGGGATTCTCCAACT |
| pUT18-*ttrR*^D58E^ | P1: GCGGGATCCAATGATCAGCAGTGAAAAGC |
|  | P2: AGGCATTCGGCTTTCAAGGATCACGCATCCGG |
|  | P3: CGTGATCCTTGAAAGCCGAATGCCTAACCTTCG |
|  | P4: GCGGGTACCCTAGATGGGATTCTCCAACT |
| pKNT25-*ttrR*-*ttrS* ^H397A^ | P1: CGGAATTCATGGGATTCTCCAACTTGCCATAAATGTAG |
|  | P2: CTGGCTGCCGAATTAAATCAACCGCTGGCAGCTA |
|  | P3: TGATTTAATTCGGCAGCCAGACTGCTGCCA |
|  | P4: CGGGATCCCATGAACAAAACGCCACGGTTGCTTCAGTT |
| pUT18-*ttrR*-*ttrS* ^H397A^ | P1: CGGAATTCATGGGATTCTCCAACTTGCCATAAATGTAG |
|  | P2: CTGGCTGCCGAATTAAATCAACCGCTGGCAGCTA |
|  | P3: TGATTTAATTCGGCAGCCAGACTGCTGCCA |
|  | P4: CGGGATCCCATGAACAAAACGCCACGGTTGCTTCAGTT |
| P*_ttrS_*-*lux* | P1:GCGGAGCTCAATACCTGCAATGGGGACCA |
|  | P2: GCGGGATCCGATGAGTCGAATCGTTCCTTC |
| P*_ttrB_*-*lux* | P1:GCGGAGCTCGCCCTGAGCTAAAGATGCAA |
|  | P2: GCGGGATCCAACTCACCTGCTGTTAGTTG |
| P*_ttrB-_*_Δbox_-*lux* | P1: GCGGAGCTCGCCCTGAGCTAAAGATGCAA |
|  | P2: ATACCCAGATCA AAGTTGATCTA |
|  | P3: TGATCTGGGTAT TATATCGACGTCGCAGCGCATT |
|  | P4: GCGGGATCCAACTCACCTGCTGTTAGTTG |
| P*_09855_* -*lux* (P*_tsdB_*-*lux*) | P1:GCGGAGCTCCCCGAGAAGGGAAAGGCGTG |
|  | P2: GCGGGATCCGCTGACTCACTTTAATTGTT |
| P*_tsdB-_*_Δbox_-*lux* | P1: GCGGAGCTCC CCGAGAAGGGAAAGGCGTG |
|  | P2: ATATTTAGAGGA TGTGAAGAGCTGCTAAATG |
|  | P3: TCCTCTAAATAT AATACCTTTAGTTATTGACAA |
|  | P4: GCGGGATCC GCTGACTCACTTTAATTGTT |
| P*_05905_-lux* | P1:CGCTCTAGAACTAGTGGATCCGAGTTGCTTCCGTATTTTAATTTAAAGT |
|  | P2:GCGGCCGCAACTAGAGGATCCACCTACATCAAATGTTGTCCTATCAGG |
| *09850* (*ttrA*)-qRT-PCR | P1:GAAGGCCGAGACGACATATT |
|  | P2:ATGCATAGCCGCAGTAAGAG |
| *04485*-qRT-PCR | P1:CCCTACTATCTGTCTCTGCTCT |
|  | P2: GTTAGTCACGTGGCTGAATTTG |
| *06270*-qRT-PCR | P1:CGGACGATAGCATCGCAATAA |
|  | P2: CTGTTTACCCAACGCAGTTCTA |
| *09835*-qRT-PCR | P1:TGCTTGTCTACTGGAACAGATG |
|  | P2: AGAAACGGCACACGAAGAA |
| *09845* (*ttrC*)-qRT-PCR | P1:CTTGACCATCGCCCTTTATACT |
|  | P2: CCCATAAATGCAGTGACAAACC |
| *09860* (*tsdA*)-qRT-PCR | P1:GCTTACGACTCACCCGAAAT |
|  | P2: ACCGCTTCATCGTTCATACC |
| *opaR*-qRT-PCR | P1:CTCGCGAAGATTTGGTTGATG |
|  | P2: GCGTGTATGTCTAGGTCGATATT |
| *22140*-qRT-PCR | P1:CGTTGCTGACAGGGTGTAATA |
|  | P2: GTCAGAGTCGTCAACCGTAAG |
| *21065*-qRT-PCR | P1:AGCGACAGCGGAACAATATAA |
|  | P2: CTTCACGGTCCATTGCTGTA |
| *21010*-qRT-PCR | P1:GGGTGACACTTGGAACGATTA |
|  | P2: TGGAATCATGCTGCCGATATAC |
| *19280*-qRT-PCR | P1:GATGCCAATGCATGCTAAAGAG |
|  | P2: GAGAATGGAGCAGGTTGTAAGT |
| 16s rRNA-qRT-PCR | P1:ACCGCCTGGGGAGTACGGTC |
|  | P2: TTGCGCTCGTTGCGGGACTT |
| P*_ttrS_*-EMSA | P1: CAATACCTGCAATGGGGACCA |
|  | P2: GATGAGTCGAATCGTTCCTTC |
| P*_ttrB_*-EMSA | P1: CGCCCTGAGCTAAAGATGCAA |
|  | P2: AACTCACCTGCTGTTAGTTG |
| P*_ttrB_*_-Δbox_-EMSA | P1: CGCCCTGAGCTAAAGATGCAA |
|  | P2: ATACCCAGATCA AAGTTGATCTA |
|  | P3: TGATCTGGGTAT TATATCGACGTCGCAGCGCATT |
|  | P4: AACTCACCTGCTGTTAGTTG |
| P*_09855_*-EMSA | P1: CCGAGAAGGGAAAGGCGTG |
|  | P2: GCTGACTCACTTTAATTGTT |
| P*_tsdB_*_-Δbox_-EMSA | P1: CCGAGAAGGGAAAGGCGTG |
|  | P2: ATATTTAGAGGA TGTGAAGAGCTGCTAAATG |
|  | P3: TCCTCTAAATAT AATACCTTTAGTTATTGACAA |
|  | P4: GCTGACTCACTTTAATTGTT |
| P*_05905_*-EMSA | P1: ATTCTGAGGAAATCAATAGTT |
|  | P2: ACACCTACATCAAATGTTGTC |
| 16s rRNA-EMSA | P1: AATGCCTAGGAAATTGCCCTG |
|  | P2: CGCCCATTGTGCAATATTCCC |
